# *e*_g_ occupancy as an effective descriptor for the catalytic activity of perovskite oxide-based peroxidase mimics

**DOI:** 10.1038/s41467-019-08657-5

**Published:** 2019-02-11

**Authors:** Xiaoyu Wang, Xuejiao J. Gao, Li Qin, Changda Wang, Li Song, Yong-Ning Zhou, Guoyin Zhu, Wen Cao, Shichao Lin, Liqi Zhou, Kang Wang, Huigang Zhang, Zhong Jin, Peng Wang, Xingfa Gao, Hui Wei

**Affiliations:** 1 College of Engineering and Applied Sciences, Nanjing National Laboratory of Microstructures, Jiangsu Key Laboratory of Artificial Functional Materials, Nanjing University, Nanjing, Jiangsu 210093 China; 2 College of Chemistry and Chemical Engineering, Jiangxi Normal University, Nanchang, 330022 China; 3 National Synchrotron Radiation Laboratory, CAS Center for Excellence in Nanoscience, University of Science and Technology of China, Hefei, 230029 China; 4 Department of Materials Science, Fudan University, Shanghai, 200433 China; 5 Key Laboratory of Mesoscopic Chemistry of MOE, School of Chemistry and Chemical Engineering, Nanjing University, Nanjing, Jiangsu 210023 China; 6 State Key Laboratory of Analytical Chemistry for Life Science, School of Chemistry and Chemical Engineering, Collaborative Innovation Center of Chemistry for Life Sciences, Nanjing University, Nanjing, Jiangsu 210023 China

## Abstract

A peroxidase catalyzes the oxidation of a substrate with a peroxide. The search for peroxidase-like and other enzyme-like nanomaterials (called nanozymes) mainly relies on trial-and-error strategies, due to the lack of predictive descriptors. To fill this gap, here we investigate the occupancy of *e*_g_ orbitals as a possible descriptor for the peroxidase-like activity of transition metal oxide (including perovskite oxide) nanozymes. Both experimental measurements and density functional theory calculations reveal a volcano relationship between the *e*_g_ occupancy and nanozymes’ activity, with the highest peroxidase-like activities corresponding to *e*_g_ occupancies of ~1.2. LaNiO_3-*δ*_, optimized based on the *e*_g_ occupancy, exhibits an activity one to two orders of magnitude higher than that of other representative peroxidase-like nanozymes. This study shows that the *e*_g_ occupancy is a predictive descriptor to guide the design of peroxidase-like nanozymes; in addition, it provides detailed insight into the catalytic mechanism of peroxidase-like nanozymes.

## Abstract (web summary)

The search for peroxidase-like as well as other enzyme-like nanozymes mainly relies on trial-and-error strategies, due to the lack of predictive descriptors. Here, the authors fill this gap by investigating the occupancy of *e*_g_ orbitals as a possible descriptor for the peroxidase-like activity of transition metal oxide nanozymes

## Introduction

Artificial enzymes aim to imitate the unique catalytic activities of natural enzymes using alternative materials. Recently, functional nanomaterials with enzyme-like catalytic activities, called nanozymes, have emerged as promising alternatives that could overcome the low stability and high cost of natural enzymes^1–13^. Intriguingly, nanozymes are superior to molecular and polymeric enzyme mimics in several ways, such as their tunable catalytic activities, large surface areas for bioconjugation, and multiple functionalities in addition to catalysis^14^. Among different nanozymes, enormous efforts have been devoted to developing nanomaterials with peroxidase-like activity (i.e., ${\mathrm{AH}}_{\mathrm{2}} + {\mathrm{H}}_{\mathrm{2}}{\mathrm{O}}_{\mathrm{2}}\frac{{{\mathrm{Peroxidase - like}}}}{{{\mathrm{nanozyme}}}}{\mathrm{A}} + {\mathrm{2H}}_{\mathrm{2}}{\mathrm{O}}$) because of their broad applications, which range from biomedical diagnosis and bioimaging to antibacterial agents and antibiofouling coatings for medical devices^1, 2, 4, 7, 10, 14–18^.

Many nanomaterials, including systems based on various transition metal oxides (TMOs), have been explored as possible peroxidase mimics^14^. For example, Yan and colleagues^1, 2, 19^ discovered the unexpected peroxidase-like activity of iron oxide nanoparticles, which were then applied to Ebola detection and tumor immunostaining. We have recently developed Ni oxide-based peroxidase mimics for glucose detection in serum^20^. However, these peroxidase-like nanozymes are generally developed using trial-and-error strategies^14^. The prevalence of empirical approaches is due to the lack of predictive descriptors—structural characteristics of the nanomaterials that can be used as proxies for their peroxidase-like activities. This lack of predictive descriptors significantly hampers the identification of more active nanozymes.

Recently, several studies have demonstrated that for electrocatalysis and photocatalysis, the *d*-band center of metals, O 2*p*-band center of TMOs, and *e*_g_ occupancy of TMOs serve as suitable activity descriptors to design efficient electro- and photo-catalysts, respectively^21–38^. However, the activity descriptors of the enzyme-mimicking nanocatalysts (e.g., peroxidase-like nanozymes) remain largely unknown^39^.

**Fig. 1:**
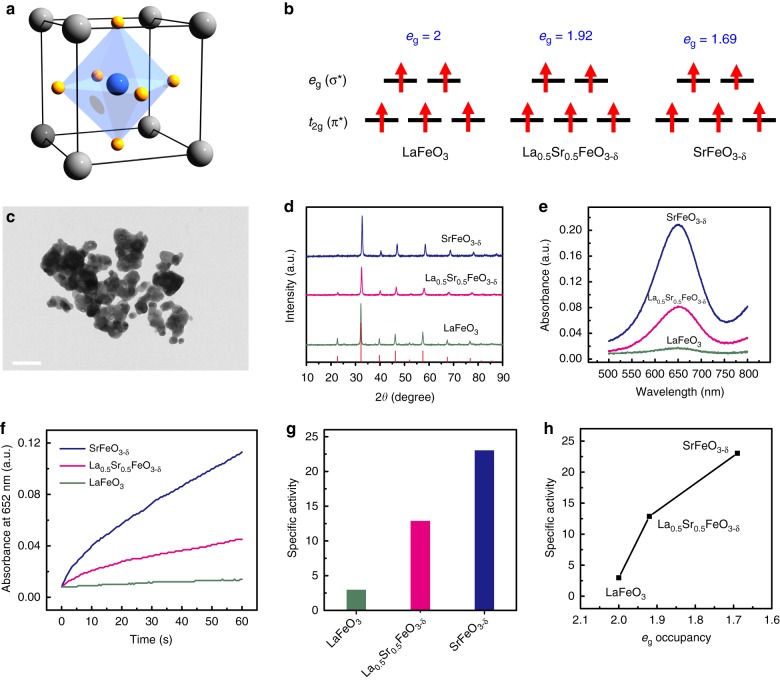
Fe-based perovskite TMOs as peroxidase mimics. **a** Schematic of ABO_3_ perovskite structure. A (rare earth or alkaline-earth metal), B (transition metal), and O are shown in gray, blue, and yellow, respectively. **b** 3*d* electron occupancy of *t*_2g_ (π*) and *e*_g_ (σ*) antibonding orbitals associated with the transition metal, for LaFeO_3_, La_0.5_Sr_0.5_FeO_3-*δ*_, and SrFeO_3-*δ*_. **c** TEM image of LaFeO_3_. Scale bar: 200 nm. **d** PXRD patterns of LaFeO_3_, La_0.5_Sr_0.5_FeO_3-*δ*_, and SrFeO_3-*δ*_ (the red lines at the bottom mark the reference pattern of LaFeO_3_ from the JCPDS database, card number 75-0541). **e** Typical absorption spectra of 0.8 mM TMB after catalytic oxidation with 50 mM H_2_O_2_ in pH 4.5 acetate buffer at 40 °C, in the presence of 10 μg mL^−1^ of LaFeO_3_, La_0.5_Sr_0.5_FeO_3-*δ*_, and SrFeO_3-*δ*_ nanozymes. **f** Time evolution of absorbance at 652 nm (A_652_) for monitoring the catalytic oxidation of 1 mM TMB with 100 mM H_2_O_2_ in the presence of 10 μg mL^−1^ of LaFeO_3_, La_0.5_Sr_0.5_FeO_3-*δ*_, and SrFeO_3-*δ*_ nanozymes. **g** Specific peroxidase-like activities of LaFeO_3_, La_0.5_Sr_0.5_FeO_3-*δ*_, and SrFeO_3-*δ*_. **h** Specific peroxidase-like activity of the Fe-based perovskite TMOs as a function of *e*_g_ occupancy. Source data are provided as a Source Data file

In this study, we aim to identify a predictive descriptor for TMO-based peroxidase mimics. We reason that the *e*_g_ occupancy (i.e., the *d*-electron population of the *e*_g_ (σ*) antibonding orbitals associated with the transition metal sites) may control the peroxidase-like activity of perovskite TMOs because of the central role of oxygen species in these biomimetic catalytic reactions. We choose ABO_3_-type perovskite TMOs with BO_6_ octahedral subunits (where A is a rare earth or alkaline-earth metal and B is a transition metal) as a model system due not only to their low cost and ease of preparation, but, more importantly, also to their diverse and controllable structural and catalytic properties (Fig. 1a)^24^, which may facilitate the tuning of the *e*_g_ occupancy by adjusting the ABO_3_ composition. We show that the peroxidase-like activity of ABO_3_-type perovskite TMOs is primarily governed by their *e*_g_ occupancy. In particular, we identify a volcano relationship between the *e*_g_ occupancy and the specific catalytic activity of perovskite TMO-based peroxidase mimics: namely, perovskite TMOs with an *e*_g_ occupancy of ~1.2 and 0 (or 2) exhibit the highest and the lowest peroxidase-like activity, respectively. These conclusions are further rationalized by density functional theory (DFT) calculations. The identified descriptor successfully predicts the peroxidase-like activity of binary TMOs with octahedral coordination geometries.

## Results

### Identification of nanozyme activity descriptor

The perovskite samples were prepared by a sol-gel method followed by annealing at the desired temperatures. The as-prepared perovskites were fully characterized by scanning electron microscope (SEM), transmission electron microscope (TEM), powder X-ray diffraction (PXRD), inductively coupled plasma-optical emission spectroscopy (ICP-OES) and Brunauer–Emmett–Teller (BET) surface area measurements (see Supplementary Figs 1–13 and Supplementary Tables 1–2 for details). The amount of oxygen vacancies in the perovskites was quantified by iodometric titrations (Supplementary Table 3). To identify a suitable descriptor for the peroxidase-like activity of perovskite TMOs, we initially examined La_1-*x*_Sr_*x*_FeO_3-*δ*_ compositions (*x* = 0–1), because the *e*_g_ occupancy of Fe in this series of perovskites could be gradually tuned by substituting La^3+^ with Sr^2+^ cation (Fig. 1b and Supplementary Table 4). Such a substitution would shift the oxidation state of Fe from + 3 in LaFeO_3_ to + 3.31 in SrFeO_3-*δ*_, resulting in the corresponding change of the *e*_g_ occupancy of Fe from 2 to 1.69 (note: as there are no fractional electrons occupying these orbitals, the *e*_g_ occupancies presented in the current study are averages between integer occupations). A representative TEM image of LaFeO_3_ (Fig. 1c) reveals the typical irregular morphology of perovskites with nanoscale features. The formation of phase-pure La_1-*x*_Sr_*x*_FeO_3-*δ*_ (*x* = 0, 0.5, and 1) perovskite structures was confirmed by matching their PXRD data to the standard pattern of LaFeO_3_ (JCPDS card number 75-0541) (Fig. 1d).

The peroxidase-like activity of the perovskite-based nanozymes was assessed by using absorption spectroscopy to monitor the catalytic oxidation of 3,3′,5,5′-tetramethylbenzidine (TMB, a typical peroxidase substrate) with H_2_O_2_ in the presence of the nanozymes. The oxidation of TMB generates an oxidized product (_ox_TMB) with a characteristic absorption peak at 652 nm. The intensity of this absorption peak (A_652_) increased with increasing Sr content and the highest absorption was obtained for SrFeO_3-*δ*_ (Fig. 1e). The time evolution of the A_652_ value (Fig. 1f) shows that SrFeO_3-*δ*_ also exhibited the fastest reaction kinetics, demonstrating that the Sr substitution effectively enhanced the peroxidase-like activity of La_1-*x*_Sr_*x*_FeO_3-*δ*_. The mass-based peroxidase-like activities of nanozymes were measured by steady-state kinetics assays (see Methods section). To separate the effect of surface area from the intrinsic peroxidase-like activity of perovskites (including the Fe-based perovskites discussed in this section), their specific activity (i.e., the mass activity normalized to the surface area) was also calculated, based on the BET surface areas obtained by nitrogen desorption measurements (Supplementary Figs 3 and 6, and Supplementary Table 1). As shown in Fig. 1g, the specific activity of SrFeO_3-*δ*_ was 7.76 and 1.79 times higher than that of LaFeO_3_ and La_0.5_Sr_0.5_FeO_3-*δ*_, respectively. The dependence of the specific activity on the Sr content of La_1-*x*_Sr_*x*_FeO_3-*δ*_ and the *e*_g_ occupancy of Fe is plotted in Fig. 1h. A substantial improvement in the peroxidase-like activity of La_1-*x*_Sr_*x*_FeO_3-*δ*_ was observed as the Sr content increased from 0 to 1 and the *e*_g_ occupancy of Fe decreased from 2 to 1.69.

To study the effect of *e*_g_ occupancy lower than 1 on the peroxidase-like activity of perovskites, we investigated three Mn-based perovskites with the *e*_g_ occupancy of Mn varying from 0.68 to ~0.08 (i.e., *e*_g_ = 0.68, 0.53, and 0.08 for LaMnO_3-*δ*_, La_0.5_Sr_0.5_MnO_3-*δ*_, and CaMnO_3-*δ*_, respectively). The SEM and TEM images shown in Supplementary Figs 4 and 5, and the PXRD patterns in Supplementary Fig. 7a demonstrate the successful synthesis of the Mn-based perovskites. As shown in Supplementary Fig. 7c, the specific activity of LaMnO_3-*δ*_ was 1.42 and 43.29 times higher than that of La_0.5_Sr_0.5_MnO_3-*δ*_ and CaMnO_3-*δ*_, respectively. Supplementary Fig. 7e shows the effect of the *e*_g_ occupancy on the specific peroxidase-like activity of the Mn-based perovskites. Different from the trend observed for the Fe-based perovskites (i.e., the peroxidase-like activity increased as the *e*_g_ occupancy decreased), the catalytic activity of the Mn-based perovskites decreased as the *e*_g_ occupancy further decreased from 0.68 to ~0.08. Taken together, the above results show a strong but non-monotonic correlation between *e*_g_ occupancy and peroxidase-like activity of perovskites, suggesting the *e*_g_ occupancy as a potential activity descriptor.

### Evaluation of *e*_g_ occupancy as nanozyme activity descriptor

**Fig. 2:**
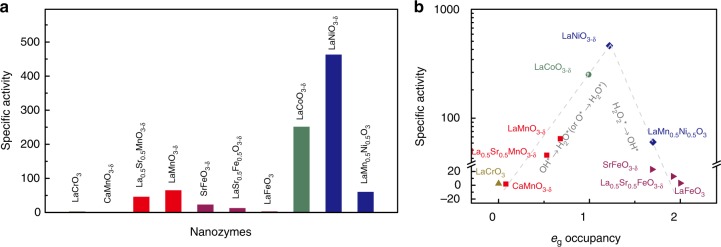
Evaluation of *e*_g_ occupancy as an effective descriptor for catalytic activity of perovskite TMO-based peroxidase mimics. **a** Specific peroxidase-like activities of perovskite TMOs. **b** Specific peroxidase-like activities of perovskite TMOs plotted as a function of *e*_g_ occupancy, in which equations shown in gray are the rate-limiting reaction steps (note: the rate-limiting steps of the catalytic reaction would be discussed in DFT calculations section). The two lines are shown for eye-guiding only. Source data are provided as a Source Data file

To further evaluate the correlation between the *e*_g_ occupancy and their peroxidase-like activity, overall ten perovskite TMOs covering *e*_g_ occupancies of 0–2, six as described above and four others (i.e., LaCrO_3_, LaCoO_3-*δ*_, LaNiO_3-*δ*_, and LaMn_0.5_Ni_0.5_O_3_) were investigated (Supplementary Figs 8–14, Supplementary Table 6, and Supplementary Notes 1–2). As shown in the summary in Fig. 2a, the perovskite TMOs exhibited significantly different specific peroxidase-like activities. Some of them (such as LaNiO_3-*δ*_) exhibited a high activity, whereas the activity of others (such as LaCrO_3_) was negligible. This behavior can be understood by plotting the activities of the ten perovskite TMOs as a function of the corresponding *e*_g_ occupancy associated with the B cations: a definitive volcano relationship is obtained (Fig. 2b). The mass-based peroxidase-like activities (i.e., mass activities) of the perovskite TMOs also show a volcano dependence on the corresponding *e*_g_ occupancies (Supplementary Fig. 15), confirming that the catalytic activity of the perovskite TMO-based peroxidase mimics is primarily governed by the *e*_g_ occupancy of the B cations. In particular, perovskite TMOs with *e*_g_ occupancy of ~1.2 exhibit the highest peroxidase-like activity (Fig. 2).

### Evaluation of other parameters as potential descriptors

As several other potential descriptors (i.e., oxidation state of transition metal, 3*d* electron number of B-site ions, O 2*p*-band center, and B-O covalency) have been studied to predict the electrocatalytic and photocatalytic activities of perovskites, we also investigated the relationship between the peroxidase-like activity and these parameters. As shown in Supplementary Fig. 16, although the oxidation state of B sites affects the peroxidase-like activity of perovskites, there is no apparent relationship between them. These results indicated that the oxidation state of B sites is not an effective descriptor and cannot provide guidance for the rational design of peroxidase-like nanozymes. We then studied the relationship between the peroxidase-like activity and the 3*d* electron number of B-site ions. As shown in Supplementary Fig. 17, an “M-shaped” relationship with the maximum peroxidase-like activities around *d*^4^ and *d*^7^ was obtained. Clearly, although the 3*d* electron number is indicative, it is not a straightforward descriptor, as two maxima are associated with it. Several recent studies suggested that the O 2*p*-band center could be a better activity descriptor than the *e*_g_ occupancy to design catalysts for oxygen reduction reaction and oxygen evolution reaction^33, 34, 40^; therefore, we also evaluated it as a potential descriptor for the peroxidase-like activity of perovskites (Supplementary Note 6). As shown in Supplementary Fig. 18, the O 2*p*-band center was not well correlated with the peroxidase-like activity of perovskites, suggesting that it is not an effective descriptor for the perovskite-based peroxidase mimics. Last, we studied the relationship between the peroxidase-like activity and B-O covalency. The B-O covalency was approximately quantified by the normalized O 1*s* → B 3*d* – O 2*p* absorbance from O K-edge X-ray absorption spectra^21^. As shown in Supplementary Fig. 19, there is no apparent relationship between the B-O covalency and the peroxidase-like activity of the six representative perovskites. Interestingly, for the perovskites with *e*_g_ occupancy close to 1 (i.e., LaMnO_3-*δ*_, LaCoO_3-*δ*_, and LaNiO_3-*δ*_), their peroxidase-like activity increases with the increasing of covalency strength of B-O. These results suggested that the B-O covalency may act as a secondary descriptor for peroxidase-like activity when the *e*_g_ occupancy of B-site is close to 1 (Supplementary Note 3).

In short, in contrast to the *e*_g_ occupancy, none of the four parameters discussed in this section showed a volcano relationship with the peroxidase-like activity of perovskites. These results further validated that the *e*_g_ occupancy as an effective activity descriptor to predict the peroxidase-like activity of perovskites.

### DFT calculations

**Fig. 3:**
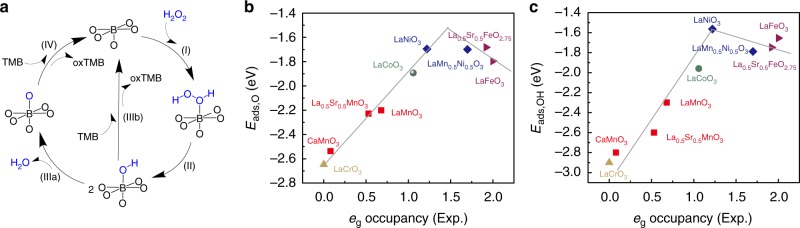
Computational analysis of the peroxidase-mimicking activity of ABO_3_ perovskite TMOs. **a** Proposed sub-processes responsible for the oxidation of TMB to _ox_TMB with the (001) facet of ABO_3_ as peroxidase mimics. **b**, **c** Adsorption energies of O (*E*_ads,O_) and OH (*E*_ads,OH_) plotted as a function of *e*_g_ occupancy, where *E*_ads_ and *e*_g_ occupancy were obtained by calculations and experiments, respectively. Source data are provided as a Source Data file

To theoretically explain the effect of *e*_g_ occupancy on the peroxidase-like activity, we performed DFT calculations for 11 ABO_3_ perovskites (i.e., LaCrO_3_, CaMnO_3_, La_0.5_Sr_0.5_MnO_3_, LaMnO_3_, LaCoO_3_, LaNiO_3_, SrFeO_3_, LaMn_0.5_Ni_0.5_O_3_, La_0.5_Sr_0.5_FeO_2.75_, LaFeO_3_, and La_0.5_Sr_0.5_FeO_3_) and proposed molecular mechanisms for the activities. The calculated geometric parameters and *e*_g_ occupancy values for these bulk structures generally agreed with the experimental ones (Supplementary Table 9 and Supplementary Figs 20–22). We proposed that these perovskites mimicked peroxidases via mechanisms of Fig. 3a: (1) the adsorption (I) and dissociation (II) of H_2_O_2_ molecules on ABO_3_ surfaces to generate the OH adsorption species; (2) the conversion of these OH adsorption species to O adsorption species, which subsequently oxidize TMB substrates (IIIa and IV); (3) or alternatively, the direct oxidization of TMB by the OH adsorption species (IIIb). Perovskite ABO_3_ (001) surfaces with the BO_2_ termination were selected as the surfaces of reactions, because transition metal B in these surfaces are all five coordinated and each has one open coordination site. The five coordinated BO_2_ termination is analogous to metals in metalloporphyrins, the active centers of many natural enzymes. The adsorption of H_2_O_2_ on perovskite (001) surfaces has no energy barriers (Supplementary Fig. 23), suggesting step I does not determine the overall reaction rate. The variations of absorption energies (*E*_ads_) for O (*E*_ads,O_) and OH (*E*_ads,OH_) with respect to *e*_g_ occupancy are shown in Fig. 3b, c, and that for H_2_O_2_ ($E_{{\mathrm{ads}},\, {\mathrm{H}}_{2}{\mathrm{O}}_{2}}$) in Supplementary Fig. 24. Volcano-like relationships were found for *E*_ads,O_ and *E*_ads,OH_ with *e*_g_ occupancy (Fig. 3b and c and Supplementary Fig. 24). Further analysis shows that the five perovskites with *e*_g_ occupancy < 1.2 (i.e., LaCrO_3_, CaMnO_3_, La_0.5_Sr_0.5_MnO_3_, LaMnO_3_, and LaCoO_3_) have strong OH* and O* adsorption energies; the other five perovskites with *e*_g_ occupancy > 1.2 (i.e., LaMn_0.5_Ni_0.5_O_3_, SrFeO_3_, La_0.5_Sr_0.5_FeO_2.5_, La_0.5_Sr_0.5_FeO_3_, and LaFeO_3_) as well as LaNiO_3_ have weak OH* and O* adsorption energies. Perovskites with *e*_g_ occupancy of ~1.2 have the weakest O and OH adsorption, in which the transfer of these oxygen species to TMB substrates is the easiest, in agreement with their highest peroxidase-mimicking activities. However, LaFeO_3_ with negligible peroxidase-like activity also possesses weak O and OH adsorption. Therefore, we reasoned that the oxidation of the substrate (i.e., IIIb and IV of Fig. 3a) is not the only rate-determining step. Reportedly, when a kinetic profile goes through a maximum as a function of a given parameter, it means that there is a change of the rate-determining step governing the reaction mechanism^32^. To identify all the rate-determining steps and to validate the mechanisms of Fig. 3a, we further calculated the energies for species involved in the proposed reaction pathways (Supplementary Figs 23–25). Supplementary Fig. 25 plots the energies of species involved in the proposed reaction pathways. It reveals that for the five perovskites with *e*_g_ occupancy < 1.2, which are all located on the left side of Fig. 2b’s volcano-like plots, the rate-determining step should be the oxidation of the substrate (i.e., IIIb and IV of Fig. 3a); for the other five with *e*_g_ occupancy > 1.2, which are all located on the right side of the volcano-like plots, the rate-determining step should be the O–O bond splitting of the adsorbed H_2_O_2_* (II of Fig. 3a); LaNiO_3_ is the maximum point where the rate-determining step changes. Taking these results together, *e*_g_ occupancy influences the peroxidase-mimicking activities of perovskites by altering the *E*_ads_ of reaction intermediates and the rate-determining step governing the catalytic reactions. Perovskites with *e*_g_ occupancy of ~1 possess optimal *E*_ads_ and can facilitate these rate-determining steps efficiently, which further lead to the high peroxidase-like activity.

### General applicability of the *e*_g_ occupancy

**Fig. 4:**
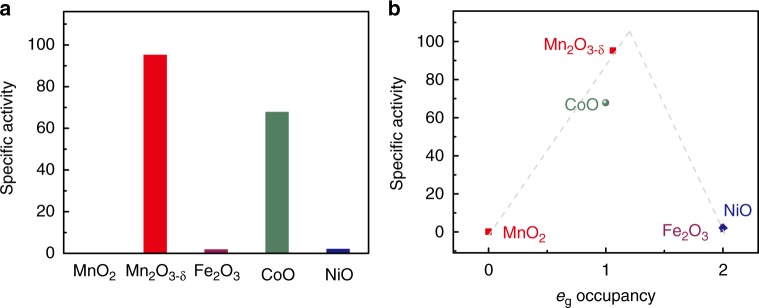
Binary TMOs as peroxidase mimics. **a** Specific peroxidase-like activities of MnO_2_, CoO, Mn_2_O_3-δ_, NiO, and Fe_2_O_3_. **b** Specific peroxidase-like activities of the binary metal oxides as a function of *e*_g_ occupancy. The two lines are shown for eye-guiding only. Source data are provided as a Source Data file

To test whether *e*_g_ occupancy could also predict the activity of non-perovskites TMOs with the same metal-oxygen octahedral coordination geometry as the perovskites described above, we investigated the peroxidase-like activity of five binary metal oxide nanoparticles (Supplementary Figs 26–28, Supplementary Table 7, and Supplementary Note 4). First, to demonstrate the predictive power of the descriptor, CoO and Mn_2_O_3-δ_ nanoparticles with unit *e*_g_ occupancy were tested, as their peroxidase-like activities are unknown. If the *e*_g_ occupancy descriptor was also applicable to the binary metal oxides, these nanoparticles would be expected to exhibit high peroxidase-like activities. As shown in Supplementary Figs 29, 30a and Fig. 4a, both nanoparticles exhibited excellent activities, in agreement with the prediction based on the *e*_g_ occupancy descriptor. By contrast, the measured peroxidase-like activities of MnO_2_ (*e*_g_ = 0), Fe_2_O_3_ (*e*_g_ = 2), and NiO (*e*_g_ = 2) nanoparticles were nearly negligible (Supplementary Fig. 29 and Fig. 4a), again in agreement with the prediction. These results clearly demonstrate that the peroxidase-like activity of binary metal oxides with octahedral coordination geometry is similarly associated with the *e*_g_ occupancy, with a similar volcano dependence to that obtained for the perovskite TMO-based peroxidase mimics (Supplementary Fig. 30b and Fig. 4b).

### Comparison with other peroxidase mimics

**Fig. 5:**
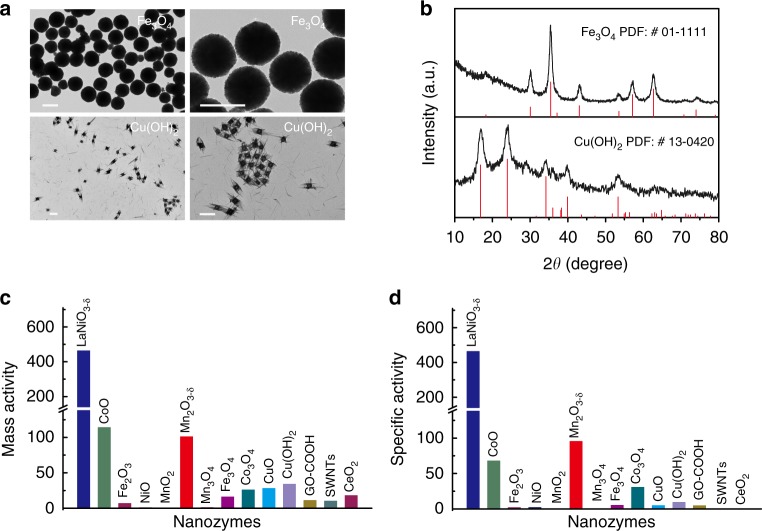
Comparison of peroxidase-like activity of LaNiO_3-*δ*_ and other nanozymes. **a** Representative TEM images of Fe_3_O_4_ and Cu(OH)_2_ at different magnifications. Scale bars: 500 nm. **b** PXRD patterns of Fe_3_O_4_ and Cu(OH)_2_ (the red lines mark the reference patterns of Fe_3_O_4_ (JCPDS card number 01-1111) and Cu(OH)_2_ (JCPDS card number 13-0420)). **c** Mass-normalized peroxidase-like activities of LaNiO_3-*δ*_ and other nanozymes. **d** Specific peroxidase-like activities of LaNiO_3-*δ*_ and other nanozymes. Source data are provided as a Source Data file

Among the 16 TMOs studied in this work (Figs 2, 4 and Supplementary Fig. 10), LaNiO_3-*δ*_ was identified as the most active peroxidase mimic, in terms of both specific and mass activities (Supplementary Note 5). Over the last decade, dozens of nanomaterials have been proposed as peroxidase mimics^41^. A comparison between the nanozymes developed in this work and those reported in the literature may be useful for searching for new nanozymes. However, a direct comparison between data produced by different studies is difficult, because the applied protocols or even the specific test conditions, such as temperature and H_2_O_2_ concentration, could significantly influence the peroxidase-like activity of the nanomaterials. To allow for a reliable and rigorous comparison, we synthesized several peroxidase mimics reported in previous studies (Fig. 5, Supplementary Methods, Supplementary Figs 26–28, 31–32, and Supplementary Table 5) and compared their peroxidase-like activities with that of LaNiO_3-*δ*_ under the same conditions. Fe_3_O_4_ nanoparticles and Cu(OH)_2_ supercages were chosen as representative peroxidase mimics for comparison: Fe_3_O_4_ nanoparticles were the first reported peroxidase mimics, whereas Cu(OH)_2_ supercages are the state-of-the-art representatives of these systems, with *K*_cat_ (catalytic constant) values comparable to those of natural peroxidase^1, 42^. Fig. 5a,b confirm the successful preparation of Fe_3_O_4_ nanoparticles and Cu(OH)_2_ supercages. The time evolution of the A_652_ parameter (Supplementary Fig. 33 and Fig. 5c) shows that the mass activity of LaNiO_3-*δ*_ is 28.9 and 13.6 times higher than that of the Fe_3_O_4_ nanoparticles and Cu(OH)_2_ supercages, respectively. Moreover, Fig. 5d shows that the specific activity of LaNiO_3-*δ*_ was 91.4 and 49.0 times higher than that of the Fe_3_O_4_ nanoparticles and Cu(OH)_2_ supercages, respectively, because of the smaller surface area of the LaNiO_3-*δ*_ nanoparticles prepared by the sol-gel method. Other representative nanozymes (such as CeO_2_, CuO, single-walled carbon nanotubes, and graphene oxide (GO-COOH)) were also investigated. The results in Fig. 5c,d confirm the superior performance of LaNiO_3-*δ*_, in terms of both specific and mass activity, further demonstrating the power of the *e*_g_ occupancy descriptor for identifying nanozymes of particularly high activity.

## Discussion

Using experimental measurements and DFT calculations, we have identified the *e*_g_ occupancy as a predictive and effective descriptor for the peroxidase-like activity of TMO (including perovskite TMO) nanomaterials. The catalytic activity of peroxidase-like nanozymes with metal-oxygen octahedral coordination geometry shows a volcano dependence on the *e*_g_ occupancy. Namely, nanozymes with *e*_g_ occupancy of ~1.2 had the highest catalytic activity, whereas *e*_g_ occupancies of 0 or 2 corresponded to negligible activities. The systematic comparison of more than 20 representative peroxidase-like nanozymes revealed that LaNiO_3-*δ*_ had the highest catalytic activity. Besides supporting an approach to the design of highly active peroxidase mimics based on the *e*_g_ occupancy, the present study also provided deep insight into the catalytic mechanism of the peroxidase-like activity of the nanozymes. Taking into account the adaptable structures and catalytic activities of TMO-based nanozymes, the current study has prompted us to further explore the application of the *e*_g_ occupancy descriptor to predict the enzyme-like activities of other metal oxides.

## Methods

### Synthesis of perovskite TMOs

The perovskite TMOs were synthesized via a sol-gel method^43^. Briefly, the respective metal nitrate salts in appropriate stoichiometric ratios (3 mmol in total) and citric acid (12 mmol) were dissolved in 100 mL of H_2_O, followed by the addition of 1.5 mL of ethylene glycol. The resulting transparent solutions were treated at 90 °C under stirring to condense them into gel, which were then decomposed at 180 °C for 5 h to form the solid precursors. The latter were decomposed at 400 °C for 2 h to remove the organic components and obtain foam precursors, which were further annealed at 700 °C (850 °C in the case of CaMnO_3-*δ*_) for 5 h with a ramp rate of 5 °C min^−1^, to obtain the final perovskite TMOs.

### Structure characterization

PXRD data were collected at room temperature using a Rigaku Ultima diffractometer using Cu Kα radiation. The diffractometer was operated at 40 kV and 40 mA, with a scan rate of 5° min^−1^ and a step size of 0.02°. TEM images were recorded on a JEOL JEM-2100 or FEI Tecnai F20 microscope at an acceleration voltage of 200 kV. SEM measurements were performed on a Hitachi S-4800 microscope operated at 5 kV. UV-visible absorption spectra were collected using a spectrophotometer (TU-1900, Beijing Purkinje General Instrument Co. Ltd, China). Nitrogen adsorption–desorption isotherms were measured at 77 K using a Quantachrome Autosorb-IQ-2C-TCD-VP analyzer and were used to calculate the surface areas of the nanozymes with the BET method. The temperature-dependent magnetization was measured on a MPMS SQUID magnetometer (MPMS-3, Quantum Design) with a magnetic field of *H* = 1 kOe under field-cooling procedures. O K-edge X-ray absorption spectroscopy (XAS) measurements were performed at the beamline BL12B-a (CMD) in Hefei Synchrotron Radiation Facility, National Synchrotron Radiation Laboratory.

### Peroxidase-like activity measurements

Steady-state kinetics assays were conducted at 37 °C in 1.0 mL cuvettes with a path length of 0.2 cm. A 0.2 M NaOAc buffer solution (pH 4.5) was used as the reaction buffer and 10 μg mL^−1^ of nanozymes were used for their kinetics assays. The kinetics data were obtained by varying the concentration of H_2_O_2_ while keeping the TMB’s concentration constant (Supplementary Table 8). The kinetics constants (i.e., *v*_max_ and *K*_m_) were calculated by fitting the reaction velocity values and the substrate concentrations to the Michaelis–Menten equation as follows:

1 $$v = \frac{{v_{{\mathrm{max}}} \times [S]}}{{K_{\mathrm{m}} + [S]}}$$

where *v* is the initial reaction velocity and *v*_max_ is maximal reaction velocity. *v*_max_ is obtained under saturating substrate conditions. [*S*] is the substrate concentration. *K*_m_, the Michaelis constant, equals to the concentration of substrate when the initial reaction velocity reaches half of its maximal reaction rate. As for TMOs with negligible activity (i.e., LaCrO_3_, LaFeO_3_, CaMnO_3-*δ*_, NiO, MnO_2_, and Mn_3_O_4_), we assumed the initial reaction velocity in the presence of 10 μg mL^−1^ of nanozymes, 1 mM TMB, and 100 mM H_2_O_2_ as the *v*_max_, because the kinetics measurements for them were difficult and not reliable.

The mass activities of the nanozymes were defined as follows:

2 $${\mathrm{Mass}}\,{\mathrm{activity}} = v_{{\mathrm{max}}}$$

The specific activities of the nanozymes were calculated from Eqs (3) and (4):

3 $${\mathrm{Specific}}\,{\mathrm{activity}} = \frac{{{\mathrm{Mass}}\,{\mathrm{activity}}}}{{{\mathrm{Normalized}}\,{\mathrm{BET}}\,{\mathrm{area}}}}$$

4 $${\mathrm{Normalized}}\,{\mathrm{BET}}\,{\mathrm{area}} = \frac{{{\mathrm{BET}}\,{\mathrm{area}}\,{\mathrm{of}}\,{\mathrm{nanozyme}}}}{{{\mathrm{BET}}\,{\mathrm{area}}\,{\mathrm{of}}\,{\mathrm{LaNiO}}_{3 - {\mathrm{\delta }}}}}$$

### DFT calculations

The bulk structure of each defect-free perovskite was modeled using the A_8_B_8_O_24_ unit cell, which was sufficiently large to consider all possible G-type antiferromagnetic (G-AFM), A-type antiferromagnetic (A-AFM), and paramagnetic (PM) magnetic orderings previously reported for perovskites (Supplementary Fig. 20). Geometrically relaxed ground-state bulk structures were then used to build the (001) slabs, each of which contained six layers: three AO and three BO_2_ (Supplementary Fig. 21). For geometry optimizations of bulks, their space group symmetries (Supplementary Table 9) were used to constrain the geometries. For geometry optimizations using slab models, atoms in the bottom two layers (i.e., one AO and one BO_2_ layer) were frozen and those in the above layers were allowed to move; lattice parameters were frozen for calculations with slab models. The generalized gradient approximation with the Perdew–Burke–Ernzerhof functional^44^ was used for all geometry optimizations and energy calculations, in a planewave basis set with an energy cut-off of 500 eV and Gaussian smearing of 0.05 eV. The Hubbard *U* correction, where *U* is defined as *U*_eff_, was applied for B metals of perovskites to treat the strong on-site Coulomb interaction of their localized *d* electrons (Supplementary Table 10)^45–47^. For calculations of bulks and slabs, the (3 × 3 × 3) and (3 × 3 × 1) Monkhorst−Pack^48^ meshes were used for the *k*-point samplings, respectively. The convergence thresholds for the electronic structure and forces were set to be 10^−5^ eV and 0.02 eV Å^−1^, respectively. All calculations were performed using the VASP code^49^. More details of the computations can be found in Supplementary Note 6.

## Supplementary information

Supplementary Information

Peer Review File

Source Data

## Data Availability

The data that support the findings of this study are available from the corresponding author upon reasonable request.
