# Supplementary material for: eg occupancy as an effective descriptor for the catalytic activity of perovskite oxide-based peroxidase mimics
Source: Nat Commun. 2019 Feb 11;10:704. doi: 10.1038/s41467-019-08657-5 (PMC6370761; doi:10.1038/s41467-019-08657-5)
Supplement: Supplementary file 1 — Supplementary Information [file 41467_2019_8657_MOESM1_ESM.pdf]

Supporting Information

e_g occupancy as an effective descriptor for the catalytic activity of perovskite oxide-based peroxidase mimics

Wang *et al*.

Table of contents

Supplementary Figure 1. Representative SEM images of $LaFeO_3$, $La_{0.5}Sr_{0.5}FeO_{3-\delta}$, and $SrFeO_{3-\delta}$.

Supplementary Figure 2. Representative TEM images of $LaFeO_3$, $La_{0.5}Sr_{0.5}FeO_{3-\delta}$, and $SrFeO_{3-\delta}$.

Supplementary Figure 3. Nitrogen adsorption-desorption isotherm curves for $LaFeO_3$, $La_{0.5}Sr_{0.5}FeO_{3-\delta}$, and $SrFeO_{3-\delta}$.

Supplementary Figure 4. Representative SEM images of $LaMnO_{3-\delta}$, $La_{0.5}Sr_{0.5}MnO_{3-\delta}$, and $CaMnO_{3-\delta}$**.**

Supplementary Figure 5. Representative TEM images of $LaMnO_{3-\delta}$, $La_{0.5}Sr_{0.5}MnO_{3-\delta}$, and $CaMnO_{3-\delta}$**.**

Supplementary Figure 6. Nitrogen adsorption-desorption isotherm curves for $LaMnO_{3-\delta}$, $La_{0.5}Sr_{0.5}MnO_{3-\delta}$, and $CaMnO_{3-\delta}$.

Supplementary Figure 7. Mn-based perovskite TMOs as peroxidase mimics.

Supplementary Figure 8. Representative SEM images of nickel contained perovskite TMOs.

Supplementary Figure 9. Representative TEM images of nickel contained perovskite TMOs.

Supplementary Figure 10. Ni-based perovskite TMOs as peroxidase mimics.

Supplementary Figure 11. Representative SEM images of $LaCoO_{3-\delta}$ and $LaCrO_3$.

Supplementary Figure 12. Representative TEM images of $LaCoO_{3-\delta}$ and $LaCrO_3$.

Supplementary Figure 13. PXRD patterns and peroxidase-like activity of $LaCoO_{3-\delta}$ and $LaCrO_3$.

Supplementary Figure 14. Magnetic measurements for $LaCoO_{3-\delta}$ perovskite oxides.

Supplementary Figure 15. (**a**) Mass normalized peroxidase-like activities of perovskite TMOs. (**b**) Mass normalized perovskite TMOs' peroxidase-like activities as a function of e_g occupancy.

Supplementary Figure 16. Evaluation of relationship between oxidation state and peroxidase-like activity of perovskites.

Supplementary Figure 17. Evaluation of relationship between 3*d*-electron number of B-site ions and peroxidase-like activity of perovskites.

Supplementary Figure 18. Evaluation of relationship between O 2*p*-band center and peroxidase-like activity of perovskites.

Supplementary Figure 19. Role of B-O covalency on the peroxidase-like activities of perovskite TMOs.

Supplementary Figure 20. Structural models for perovskite bulks.

Supplementary Figure 21. Structural models for perovskite slabs.

Supplementary Figure 22. Comparison between calculated and experimental e_g values for perovskites.

Supplementary Figure 23. Energy profile for the adsorption of H_2O_2 to the $LaNiO_3$ (001) surface.

Supplementary Figure 24. The adsorption energies of H_2O_2 plotted as a function of e_g occupancy.

Supplementary Figure 25. Relative energies for intermediates involved in the catalytic reactions proposed in Figure 3a.

Supplementary Figure 26. Representative SEM images of binary TMOs. (**a**, **b**) MnO_2, (**c**, **d**) $Mn_2O_{3-\delta}$, (**e**, **f**) CoO, (**g**, **h**) NiO, and (**i**, **j**) Fe_2O_3.

Supplementary Figure 27. Representative TEM images of binary TMOs. (**a**, **b**) MnO_2, (**c**, **d**) $Mn_2O_{3-\delta}$, (**e**, **f**) CoO, (**g**, **h**) NiO, and (**i**, **j**) Fe_2O_3.

Supplementary Figure 28. PXRD patterns of (**a**) MnO_2, (**b**) $Mn_2O_{3-\delta}$, (**c**) CoO, (**d**) NiO, and (**e**) Fe_2O_3.

Supplementary Figure 29. Kinetic curves of A_{652} for monitoring the catalytic oxidation of 1 mM TMB with 100 mM H_2O_2 in the presence of 10 $\mu g \cdot mL^{-1}$ MnO_2, CoO, $Mn_2O_{3-\delta}$, NiO, and Fe_2O_3, respectively.

Supplementary Figure 30. Mass normalized peroxidase-like activities of binary TMOs.

Supplementary Figure 31. Representative TEM images of other peroxidase mimics reported by references. (**a**, **b**) Mn_3O_4, (**c**, **d**) Fe_3O_4, (**e**, **f**) Co_3O_4, and (**g**, **h**) $Cu(OH)_2$.

Supplementary Figure 32. Representative TEM images of other peroxidase mimics reported by references. (**a**, **b**) CuO, (**c**, **d**) GO-COOH, (**e**, **f**) SWNT, and (**g**, **h**) CeO_2.

Supplementary Figure 33. Kinetic curves of A_{652} for monitoring the catalytic oxidation of 1 mM TMB with 100 mM H_2O_2 in the presence of 10 $\mu g \cdot mL^{-1}$ $LaNiO_{3-\delta}$ and other typical nanozymes reported by references.

Supplementary Table 1. BET surface area of the TMOs (including perovskite TMOs) studied in this work.

Supplementary Table 2. ICP-OES analysis of $LaMn_{0.5}Ni_{0.5}O_3$, $La_{0.5}Sr_{0.5}MnO_3$, and $La_{0.5}Sr_{0.5}FeO_3$.

Supplementary Table 3. Oxygen vacancy (δ), oxidation state of B site, and nominal e_g occupancy.

Supplementary Table 4. Transition metal's valence, spin state, and assignment of e_g electron occupancy for the TMOs (including perovskite TMOs).

Supplementary Table 5. BET surface area of other nanomaterials studied in this work.

Supplementary Table 6. Effective magnetic moment (μ_{eff}) and e_g occupancy for different spin states of cobalt ion in $LaCoO_{3-\delta}$.

Supplementary Table 7. Comparison of literature results and ours on the peroxidase-like activities of several binary TMOs.

Supplementary Table 8. Kinetics parameters of TMOs as well as other representative nanozymes.

Supplementary Table 9. The ground state magnetic orderings, space groups, and lattice parameters for ABO_3 perovskites obtained by calculations with the corresponding references.

Supplementary Table 10. Collection of the U_{eff} values for *d* orbitals of transition metals.

Supplementary Notes.

Supplementary Methods.

Supplementary References.

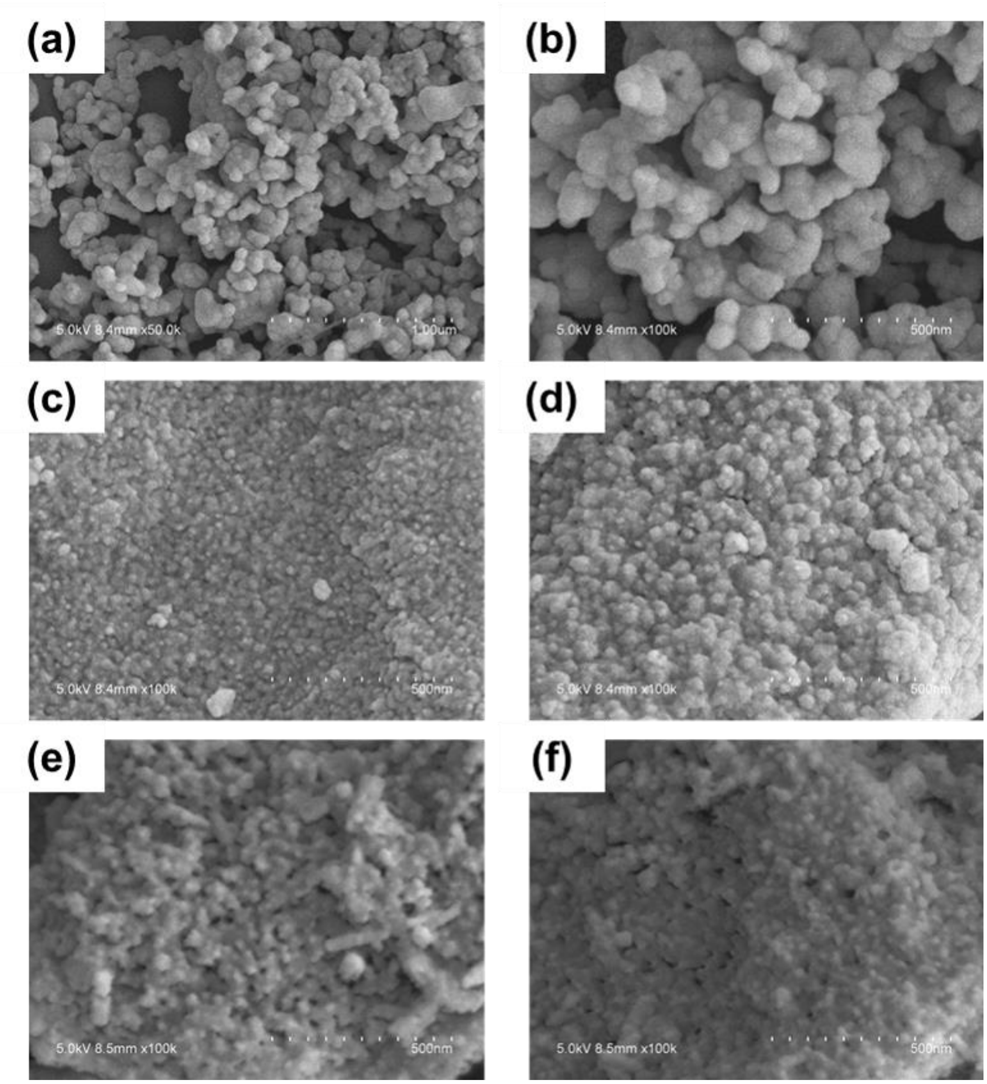

Supplementary Figure 1. Representative SEM images of (**a**, **b**) $LaFeO_3$, (**c**, **d**) $La_{0.5}Sr_{0.5}FeO_{3-\delta}$, and (**e**, **f**) $SrFeO_{3-\delta}$. Source data are provided as a Source Data file.

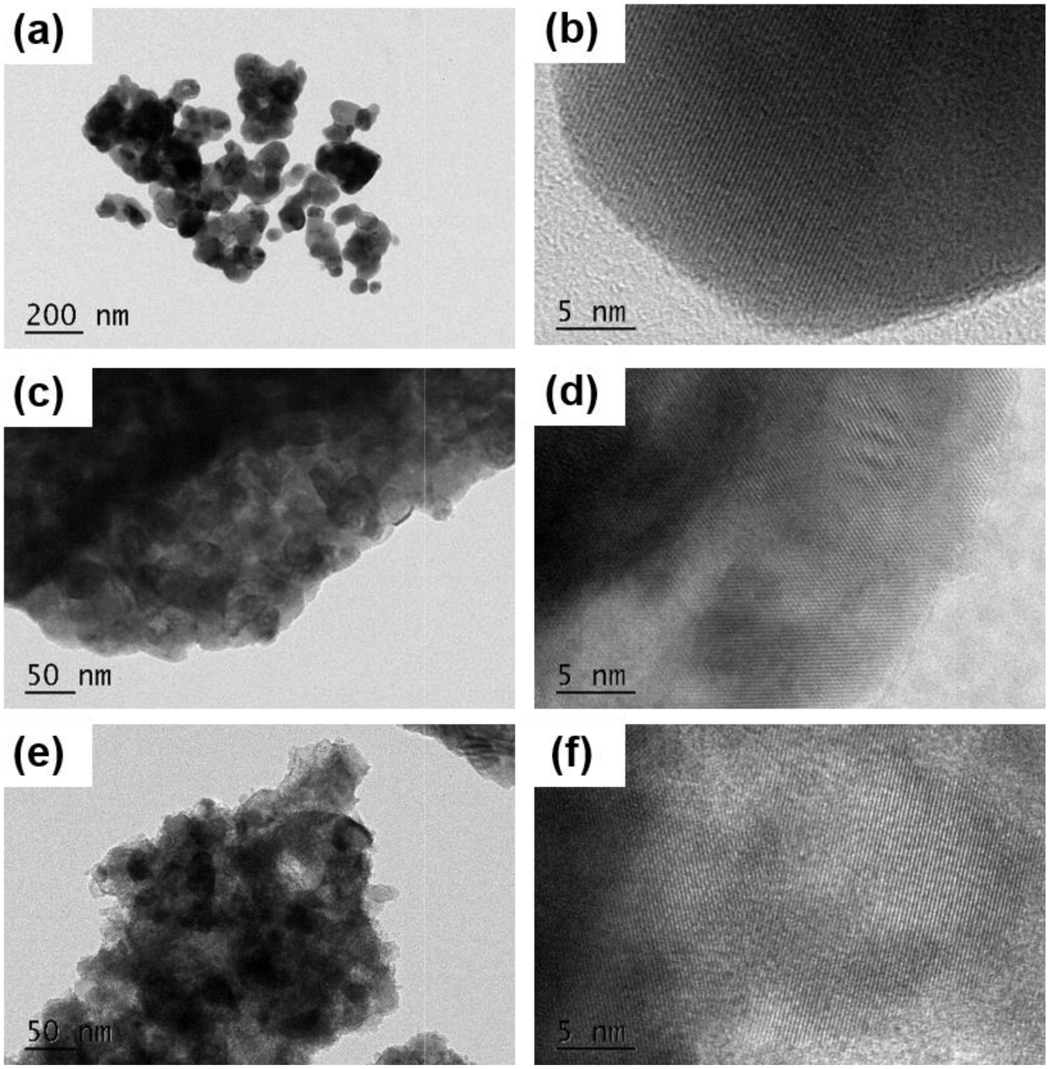

Supplementary Figure 2. Representative TEM images of (**a**, **b**) $LaFeO_3$, (**c**, **d**) $La_{0.5}Sr_{0.5}FeO_{3-\delta}$, and (**e**, **f**) $SrFeO_{3-\delta}$. Source data are provided as a Source Data file.

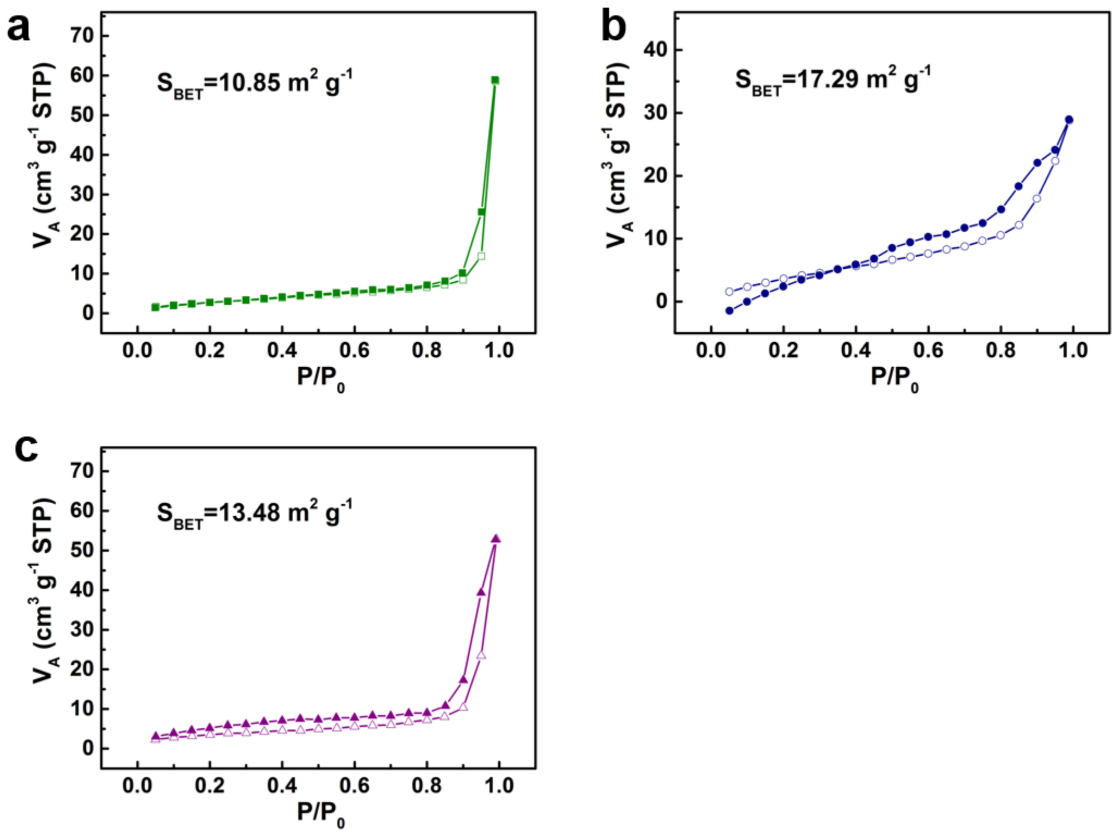

Supplementary Figure 3. Nitrogen adsorption-desorption isotherm curves for (**a**) $LaFeO_3$, (**b**) $La_{0.5}Sr_{0.5}FeO_{3-\delta}$, and (**c**) $SrFeO_{3-\delta}$. Source data are provided as a Source Data file.

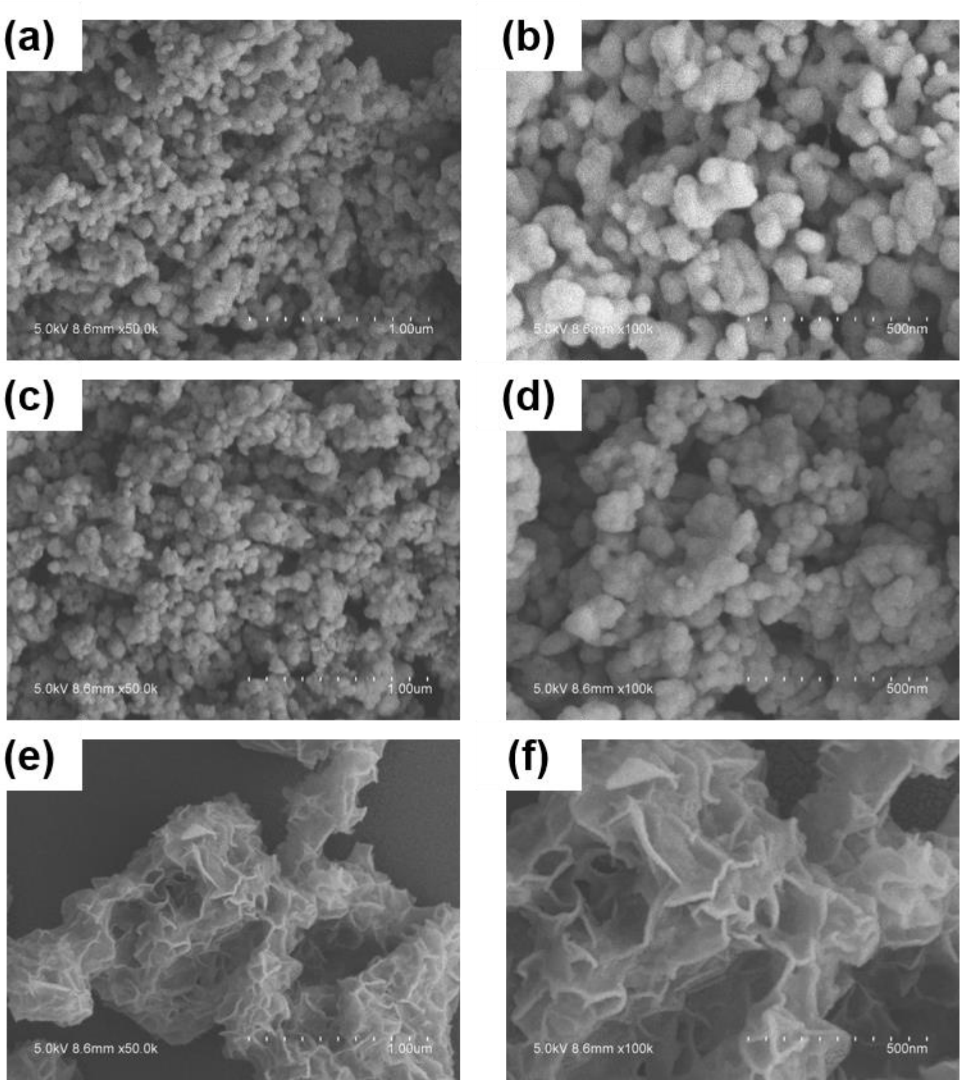

Supplementary Figure 4. Representative SEM images of (**a**, **b**) $LaMnO_{3-\delta}$, (**c**, **d**) $La_{0.5}Sr_{0.5}MnO_{3-\delta}$, and (**e**, **f**) $CaMnO_{3-\delta}$**.** Source data are provided as a Source Data file.

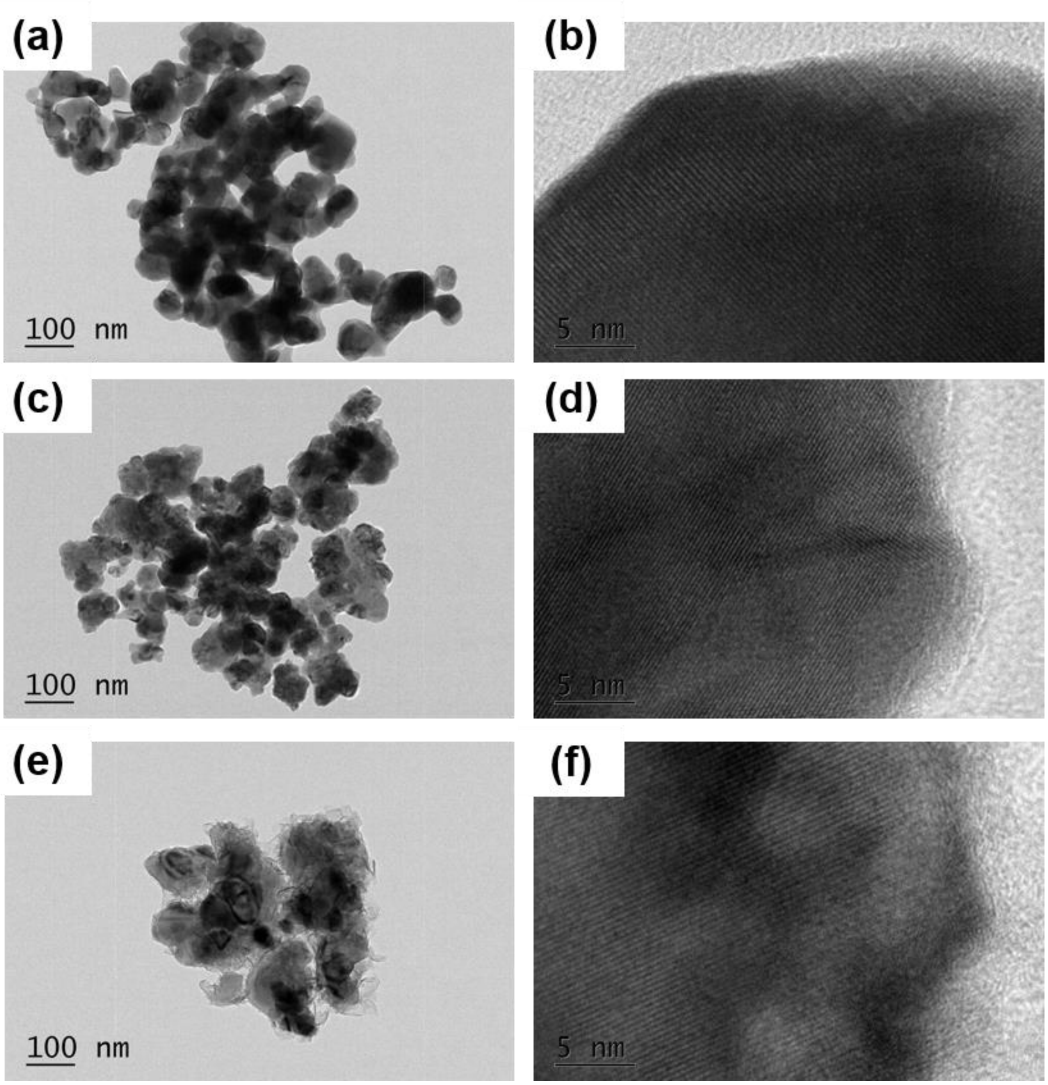

Supplementary Figure 5. Representative TEM images of (**a**, **b**) $LaMnO_{3-\delta}$, (**c**, **d**) $La_{0.5}Sr_{0.5}MnO_{3-\delta}$, and (**e**, **f**) $CaMnO_{3-\delta}$**.** Source data are provided as a Source Data file.

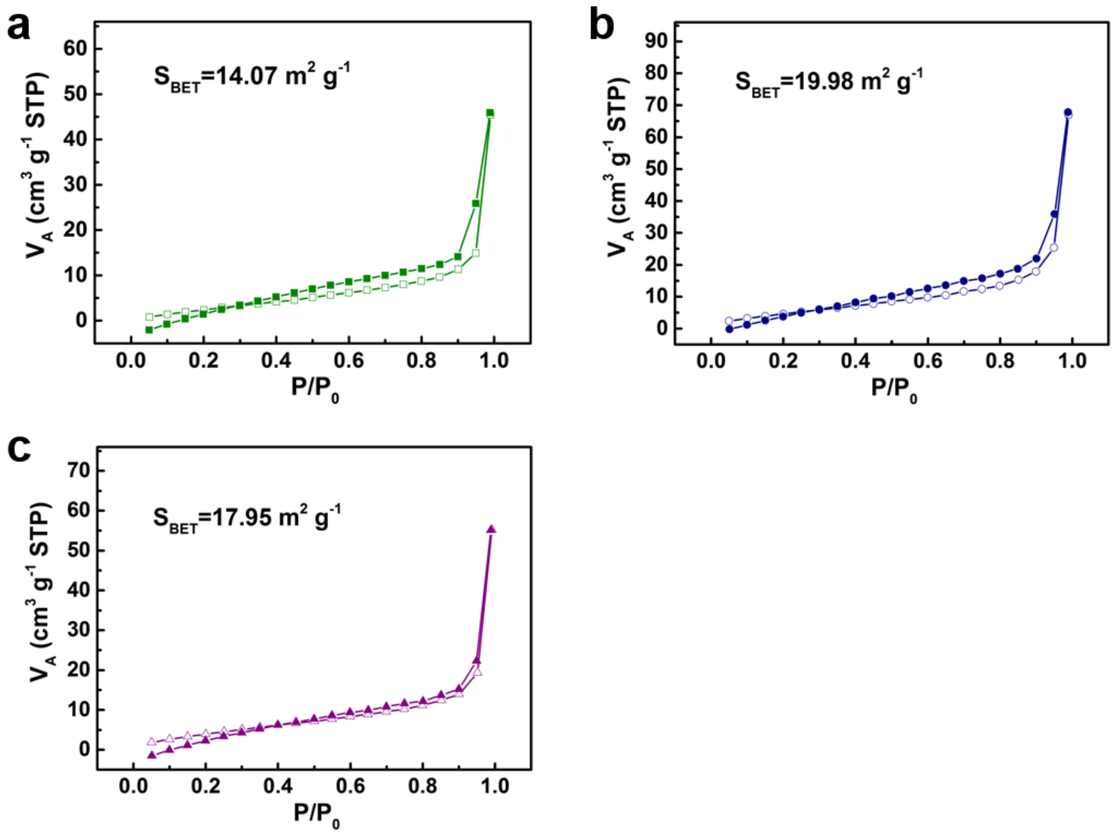

Supplementary Figure 6. Nitrogen adsorption-desorption isotherm curves for (**a**) $LaMnO_{3-\delta}$, (**b**) $La_{0.5}Sr_{0.5}MnO_{3-\delta}$, and (**c**) $CaMnO_{3-\delta}$. Source data are provided as a Source Data file.

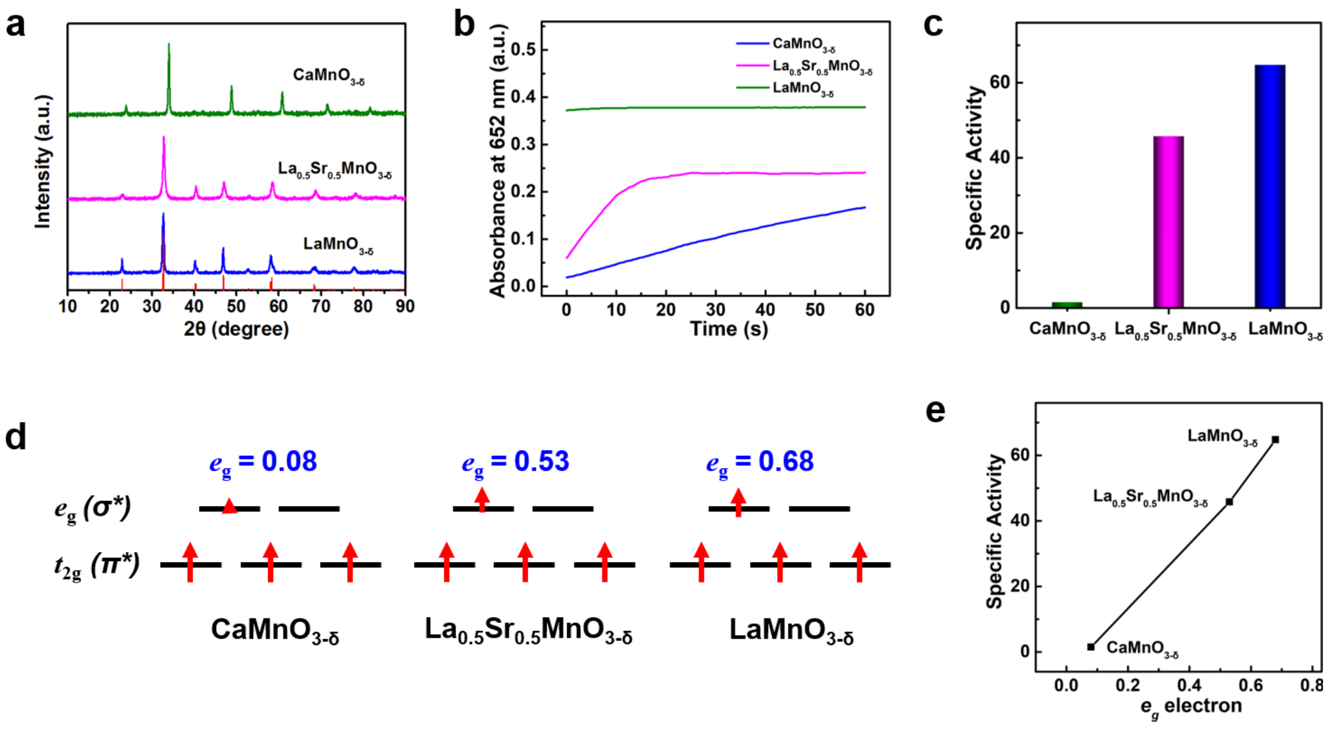

Supplementary Figure 7. Mn-based perovskite TMOs as peroxidase mimics. (**a**) PXRD patterns of $CaMnO_{3-\delta}$, $La_{0.5}Sr_{0.5}MnO_{3-\delta}$, and $LaMnO_{3-\delta}$ (the red lines at the bottom mark the reference pattern of $LaMnO_3$ from the JCPDS database, card No. 89-2470). (**b**) Kinetic curves of A_{652} for monitoring the catalytic oxidation of 1 mM TMB with 100 mM H_2O_2 in the presence of 10 μg·mL^{-1} $CaMnO_{3-\delta}$, $La_{0.5}Sr_{0.5}MnO_{3-\delta}$, and $LaMnO_{3-\delta}$, respectively. (**c**) Surface area normalized peroxidase-like activities of $CaMnO_{3-\delta}$, $La_{0.5}Sr_{0.5}MnO_{3-\delta}$, and $LaMnO_{3-\delta}$. (**d**) Transition metal's 3*d* electron occupancy of $CaMnO_{3-\delta}$, $La_{0.5}Sr_{0.5}MnO_{3-\delta}$, and $LaMnO_{3-\delta}$ in split t_{2g} (π*) and e_g (σ*) antibonding orbitals. (**e**) Surface area normalized peroxidase-like activities of Mn contained perovskite TMOs as a function of e_g occupancy. Source data are provided as a Source Data file.

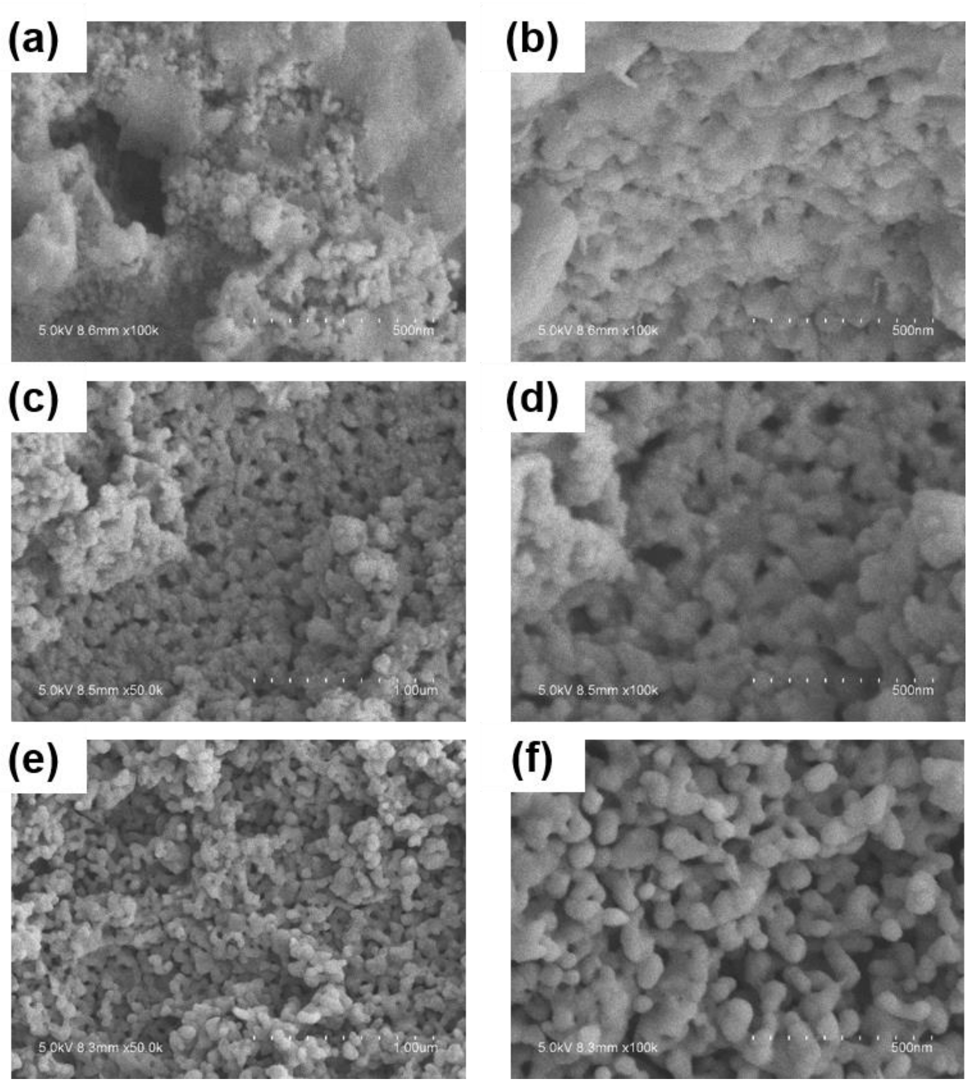

Supplementary Figure 8. Representative SEM images of nickel contained perovskite TMOs. (**a**, **b**) $LaNiO_{3-\delta}$, (**c**, **d**) $LaNiO_{3-\delta}$-H_2, and (**e**, **f**) $LaMn_{0.5}Ni_{0.5}O_3$. Source data are provided as a Source Data file.

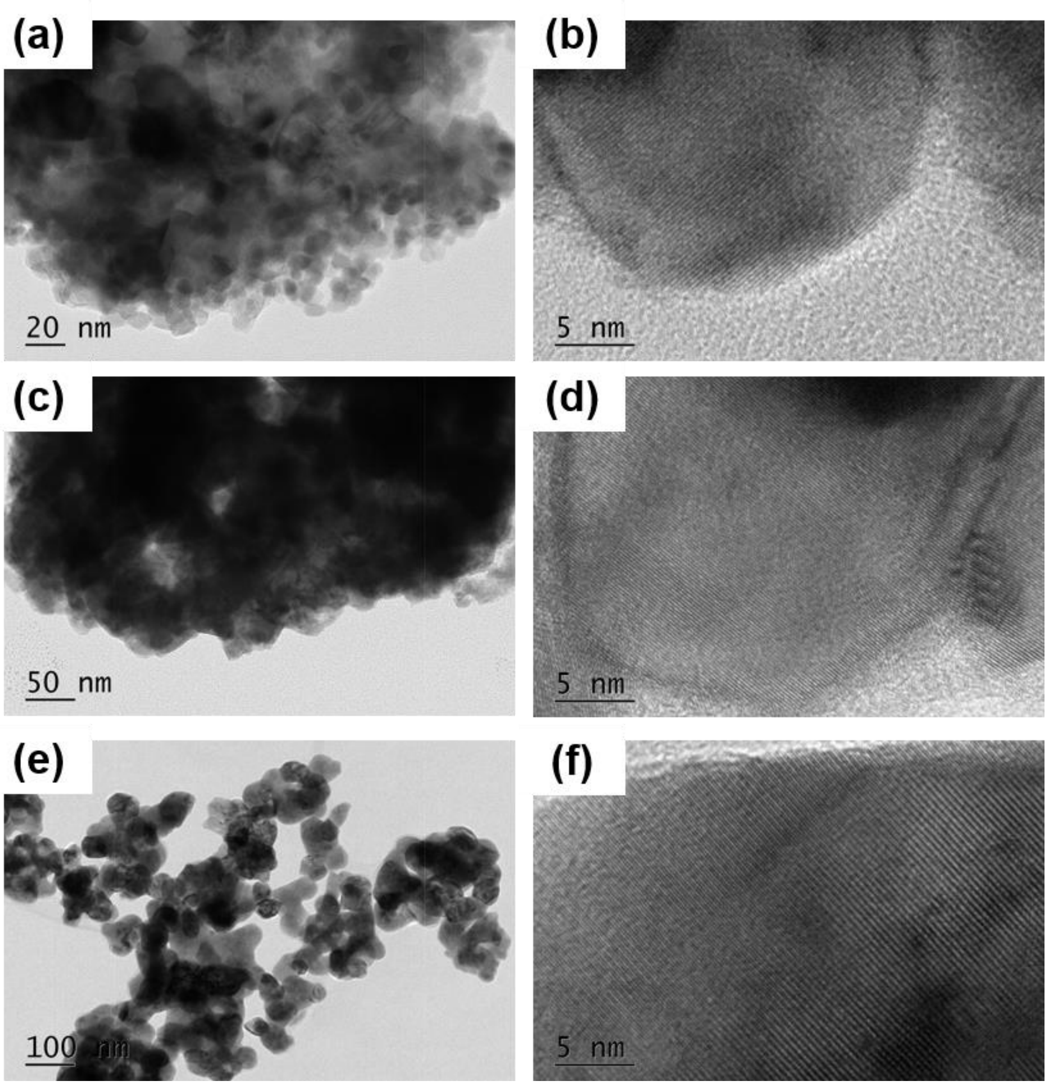

Supplementary Figure 9. Representative TEM images of nickel contained perovskite TMOs. (**a**, **b**) $LaNiO_{3-\delta}$, (**c**, **d**) $LaNiO_{3-\delta}$-H_2, and (**e**, **f**) $LaMn_{0.5}Ni_{0.5}O_3$. Source data are provided as a Source Data file.

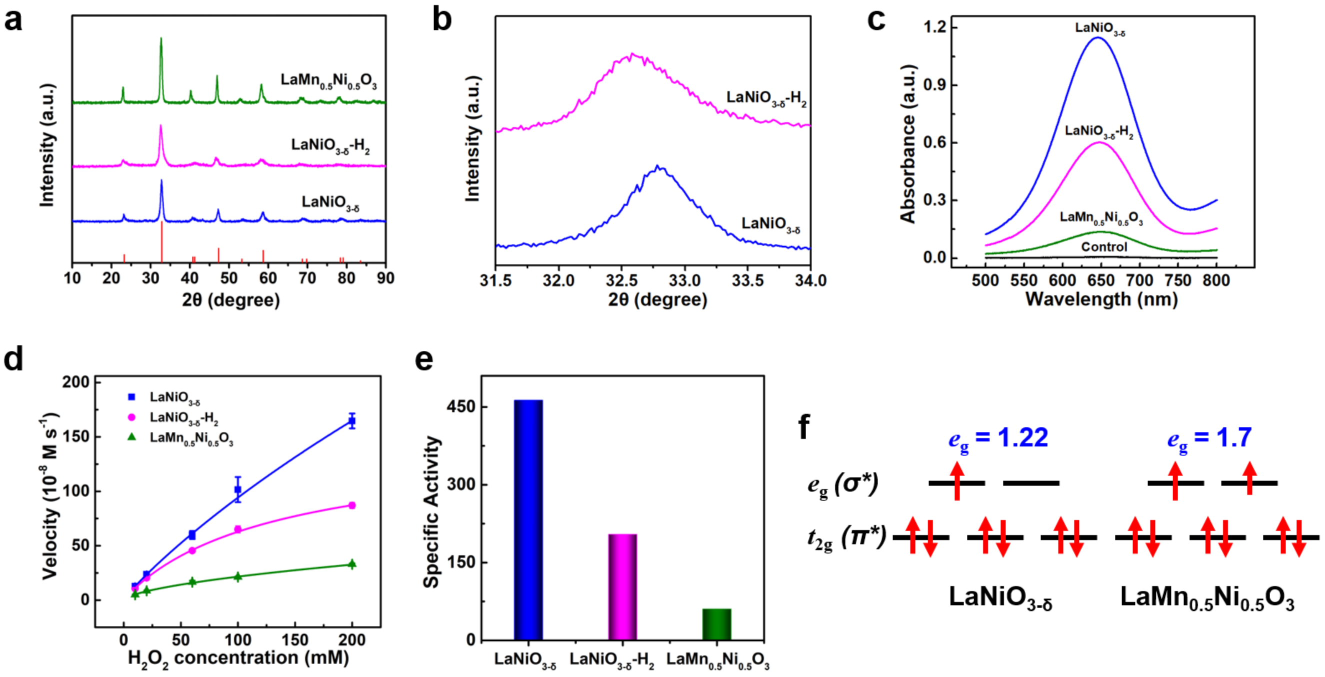

Supplementary Figure 10. Ni-based perovskite TMOs as peroxidase mimics. (**a**) Powder X-ray diffraction (PXRD) patterns of $LaNiO_{3-\delta}$, $LaNiO_{3-\delta}$-H_2, and $LaMn_{0.5}Ni_{0.5}O_3$ (the red lines at the bottom mark the reference pattern of $LaNiO_3$ from the JCPDS database, card No. 34-1181). (**b**) PXRD patterns of $LaNiO_{3-\delta}$ and $LaNiO_{3-\delta}$-H_2 in the region of 2θ = 31.5-34°. (**c**) Typicial absorption spectra of 0.8 mM TMB catalytically oxidized by 10 μg·mL^{-1} nanozymes (*i.e.*, Control, $LaNiO_{3-\delta}$, $LaNiO_{3-\delta}$-H_2, and $LaMn_{0.5}Ni_{0.5}O_3$) in the presence of 50 mM H_2O_2 in pH 4.5 acetate buffer at 40 ℃. (**d**) Michaelis-Menten plots with varying concentration of H_2O_2 for $LaNiO_{3-\delta}$, $LaNiO_{3-\delta}$-H_2, and $LaMn_{0.5}Ni_{0.5}O_3$, respectively. (**e**) Surface area normalized peroxidase-like activities of $LaNiO_{3-\delta}$, $LaNiO_{3-\delta}$-H_2, and $LaMn_{0.5}Ni_{0.5}O_3$. (**f**) Transition metal's 3*d* electron occupancy of $LaNiO_{3-\delta}$ and $LaMn_{0.5}Ni_{0.5}O_3$ in split t_{2g} (π*) and e_g (σ*) antibonding orbitals. Source data are provided as a Source Data file.

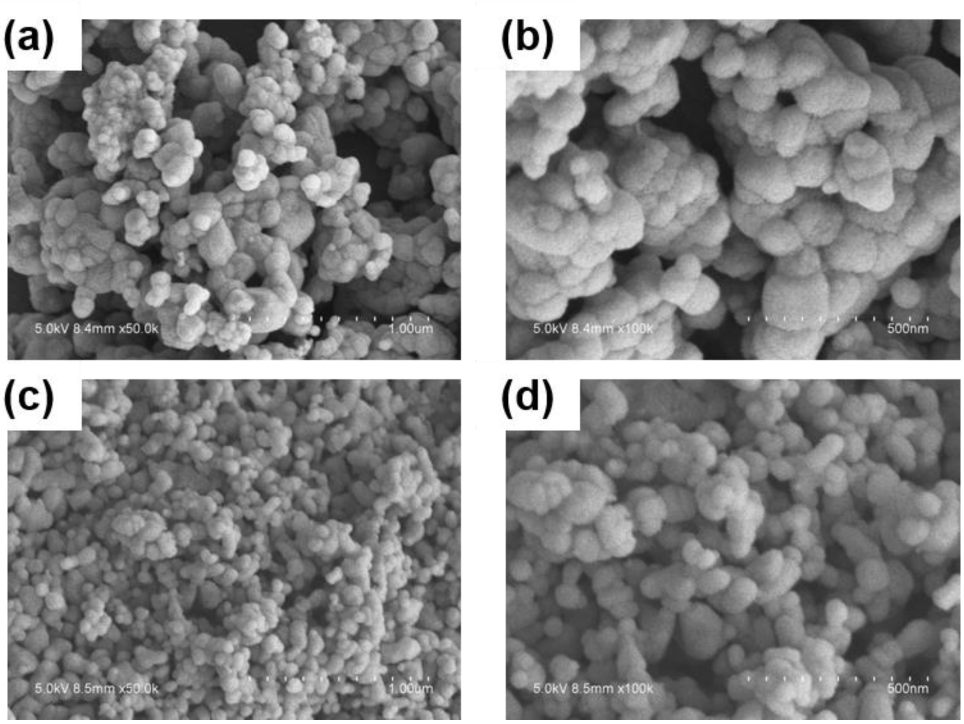

Supplementary Figure 11. Representative SEM images of (**a**, **b**) $LaCoO_{3-\delta}$ and (**c**, **d**) $LaCrO_3$. Source data are provided as a Source Data file.

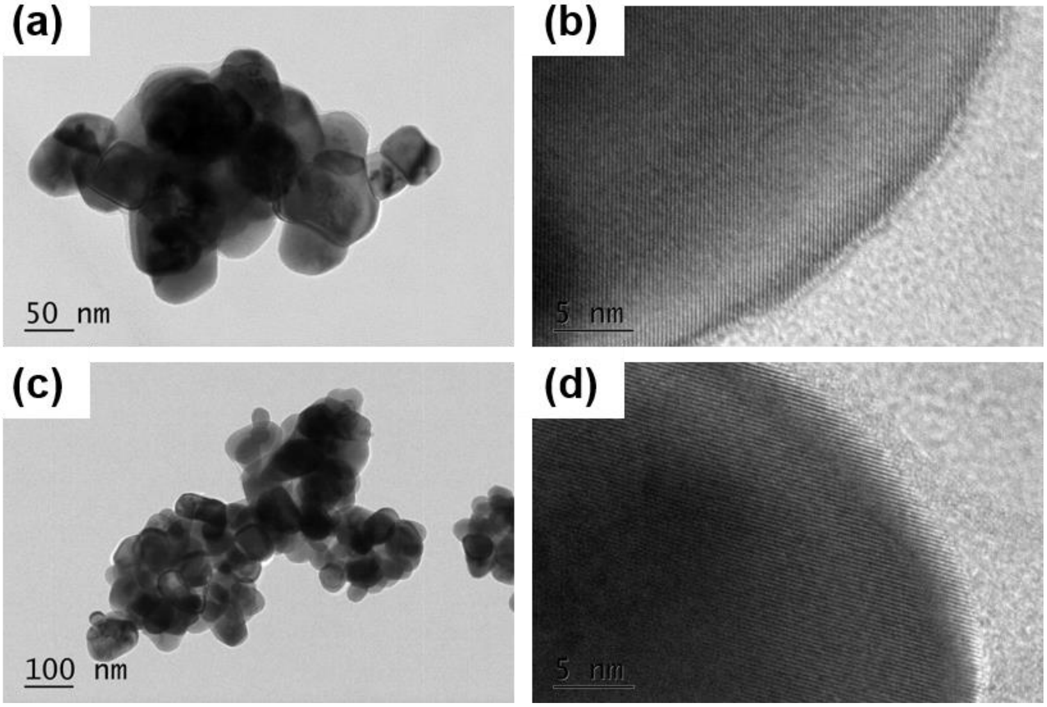

Supplementary Figure 12. Representative TEM images of (**a**, **b**) $LaCoO_{3-\delta}$ and (**c**, **d**) $LaCrO_3$. Source data are provided as a Source Data file.

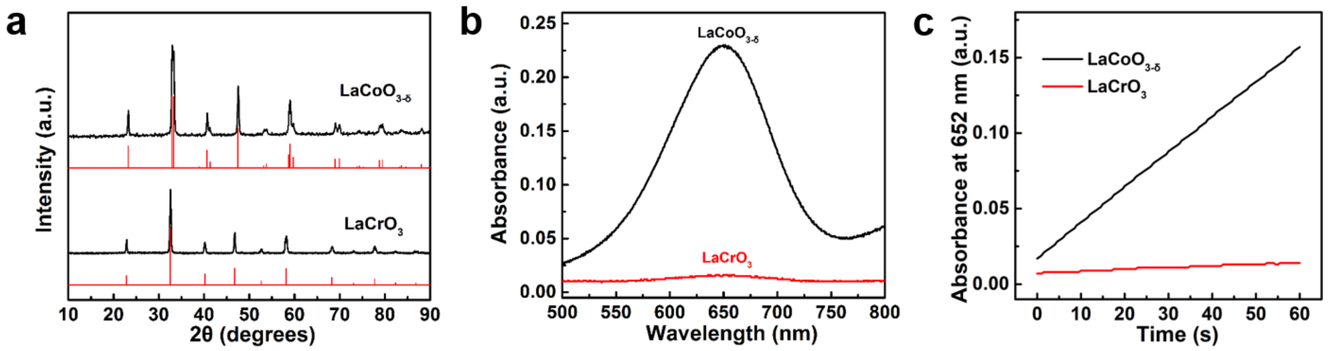

Supplementary Figure 13. (**a**) PXRD patterns of $LaCoO_{3-\delta}$ and $LaCrO_3$ (Red curve: reference patterns of $LaCoO_3$ and $LaCrO_3$ from the JCPDS database, card No. 84-0848 and 75-0441). (**b**) Typicial absorption spectra of 0.8 mM TMB catalytically oxidized by 10 μg·mL^{-1} nanozymes (*i.e.*, $LaCoO_{3-\delta}$ and $LaCrO_3$) in the presence of 50 mM H_2O_2 in pH 4.5 acetate buffer at 40 ℃. (**c**) Kinetic curves of A_{652} for monitoring the catalytic oxidation of 1 mM TMB with 100 mM H_2O_2 in the presence of 10 μg·mL^{-1} $LaCoO_{3-\delta}$ and $LaCrO_3$. Source data are provided as a Source Data file.

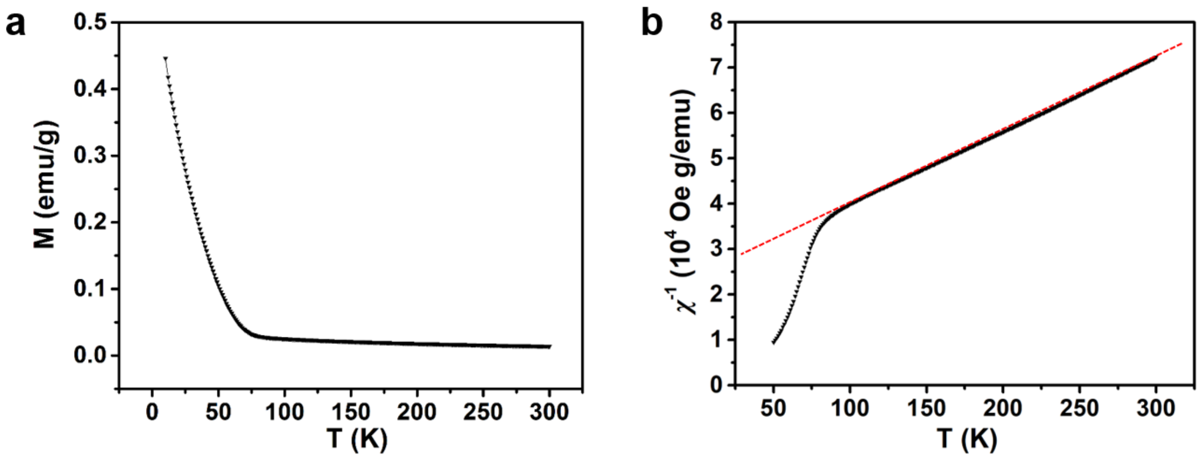

Supplementary Figure 14. Magnetic measurements for $LaCoO_{3-\delta}$ perovskite oxides. The temperature dependent magnetization (**a**) and inverse susceptibilities (**b**) under H = 1 kOe for $LaCoO_{3-\delta}$ nanoparticles. The dotted lines showed the fitting result by a Curie-Weiss law. Source data are provided as a Source Data file.

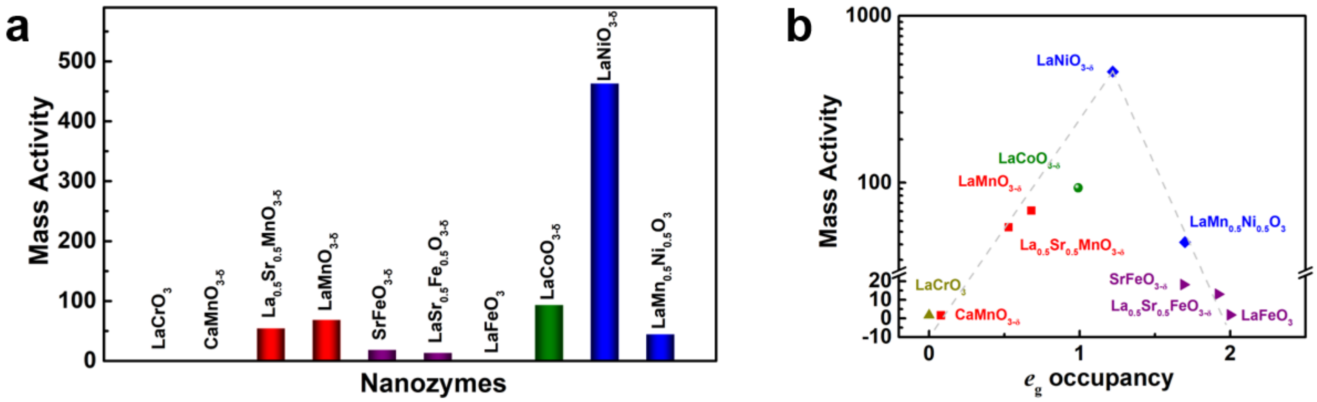

Supplementary Figure 15. (**a**) Mass normalized peroxidase-like activities of perovskite TMOs. (**b**) Mass normalized perovskite TMOs' peroxidase-like activities as a function of e_g occupancy. Source data are provided as a Source Data file.

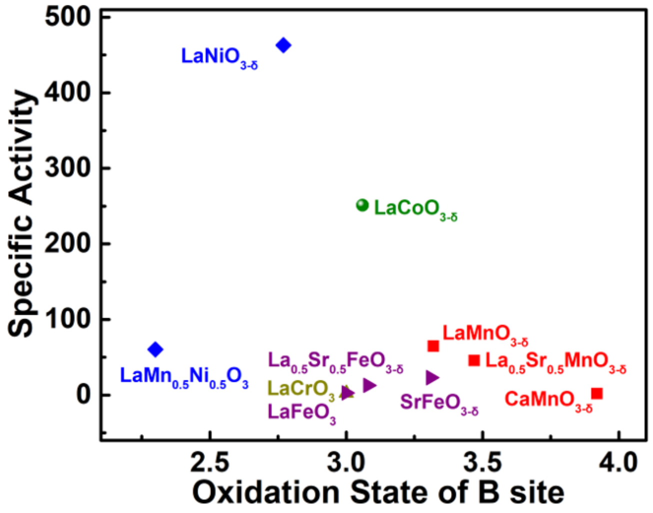

Supplementary Figure 16. Evaluation of relationship between oxidation state and peroxidase-like activity of perovskites. Specific peroxidase-like activities of perovskite TMOs versus oxidation station of B site metal ions. Source data are provided as a Source Data file.

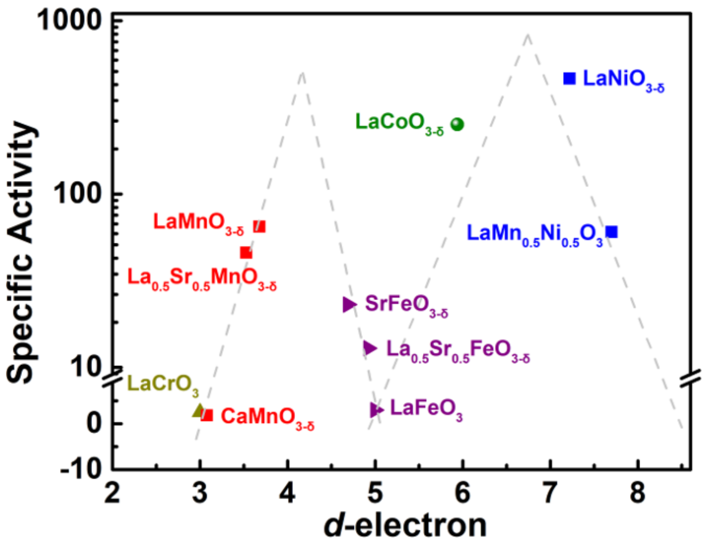

Supplementary Figure 17. Evaluation of relationship between 3*d*-electron number of B-site ions and peroxidase-like activity of perovskites. Specific peroxidase-like activities of perovskite TMOs versus 3*d*-electron number of B-site ions. Source data are provided as a Source Data file.

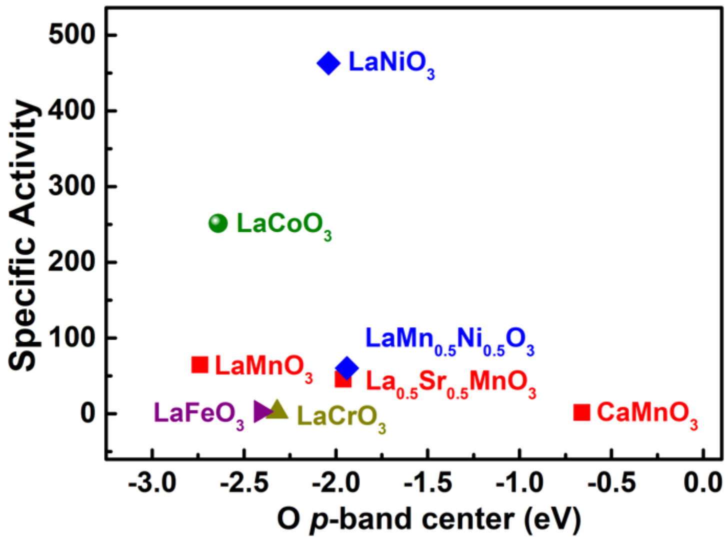

Supplementary Figure 18. Evaluation of relationship between O 2*p*-band center and peroxidase-like activity of perovskites. Experimentally specific peroxidase-like activities of perovskite TMOs plotted as a function of the calculated bulk O 2*p*-band center. Source data are provided as a Source Data file.

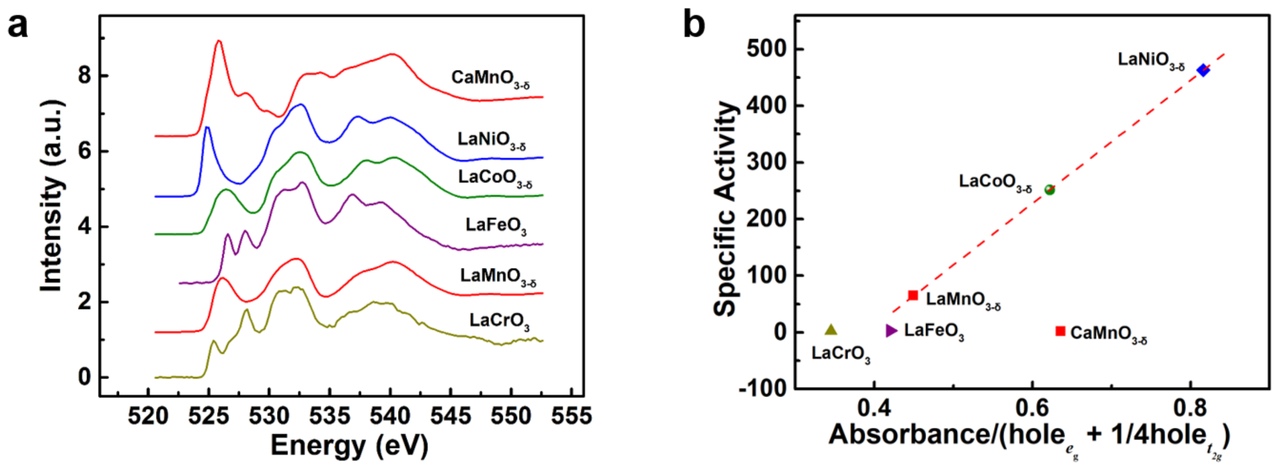

Supplementary Figure 19. Role of B-O covalency on the peroxidase-like activities of perovskite TMOs. (**a**) O K-edge XAS of $LaCrO_3$, $LaMnO_{3-\delta}$, $LaFeO_3$, $LaCoO_{3-\delta}$, $LaNiO_{3-\delta}$, and $CaMnO_{3-\delta}$. (**b**) Specific peroxidase-like activities of perovskite TMOs plotted as a function of B-O covalency. The line is shown for eye-guiding only. Source data are provided as a Source Data file.

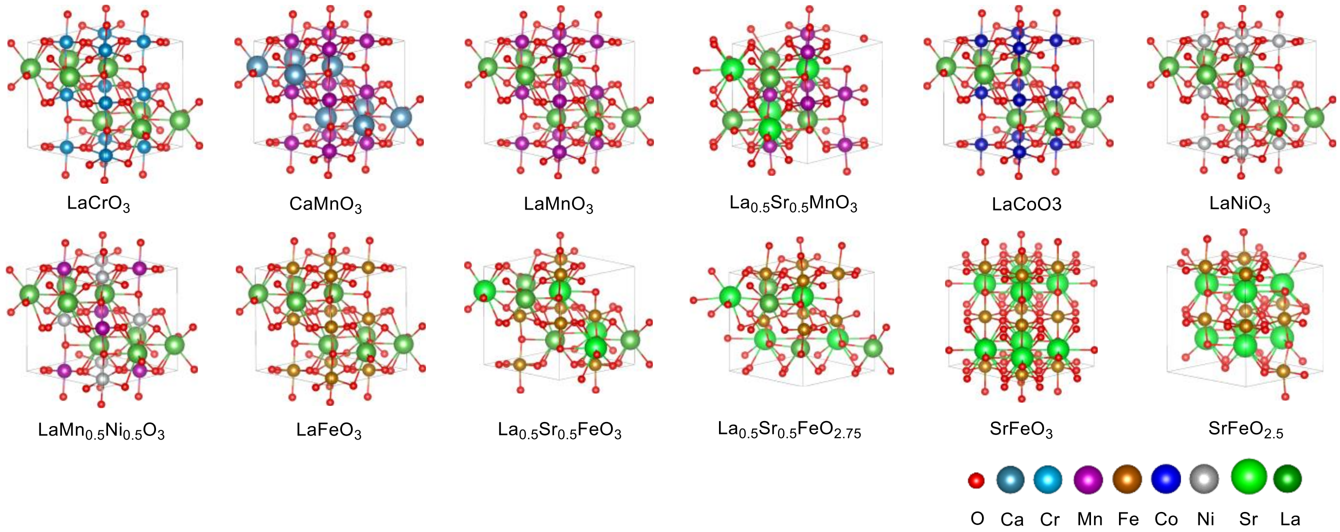

Supplementary Figure 20. Structural models for perovskite bulks.

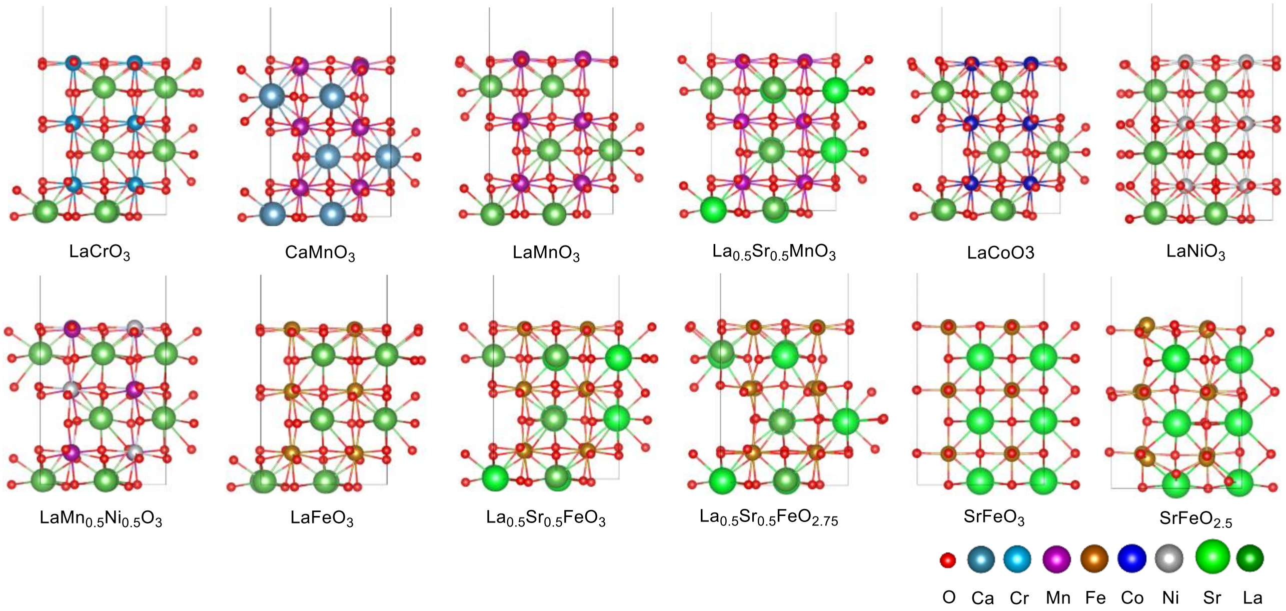

Supplementary Figure 21. Structural models for perovskite slabs.

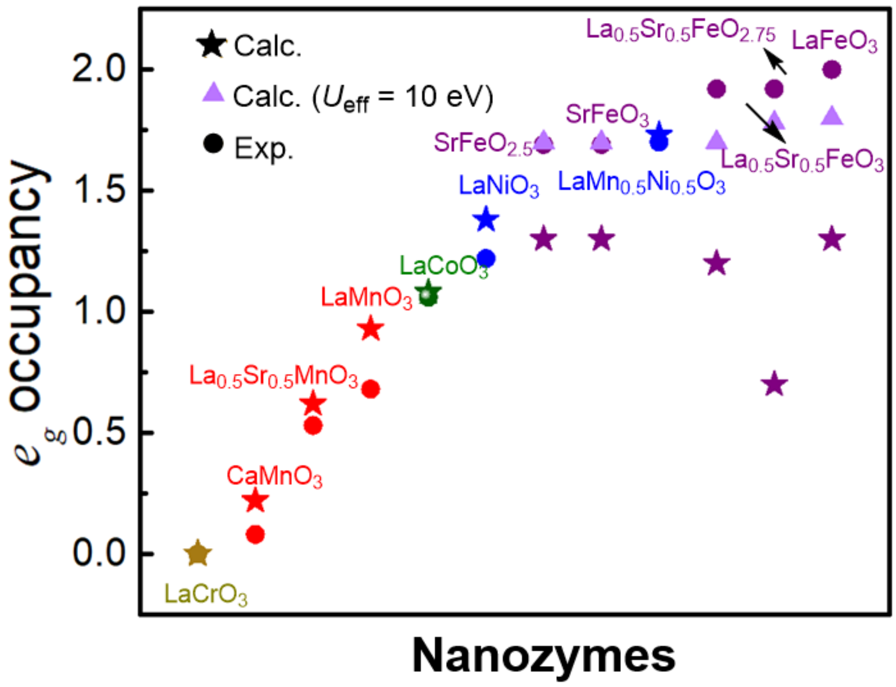

Supplementary Figure 22. Comparison between calculated and experimental e_g values for perovskites. Both sets of e_g values agree well with each other, except for perovskites containing irons. Calculations with U_{eff} = 10.0 eV for Fe 3d orbitals make the calculated and experimental e_g values for iron containing perovskites more consistent. Source data are provided as a Source Data file.

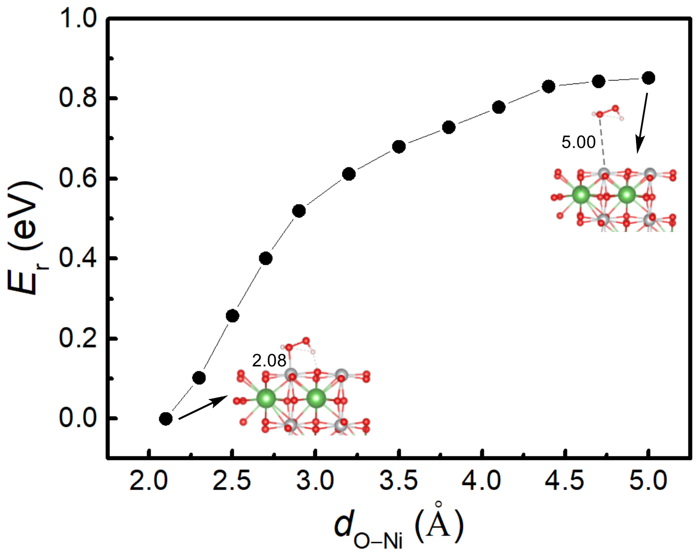

Supplementary Figure 23. Energy profile for the adsorption of H_2O_2 to the $LaNiO_3$ (001) surface. The energy of each point (adsorption strcuture) was caclulated by geomery optimization with some geometric paramters constrained. Source data are provided as a Source Data file.

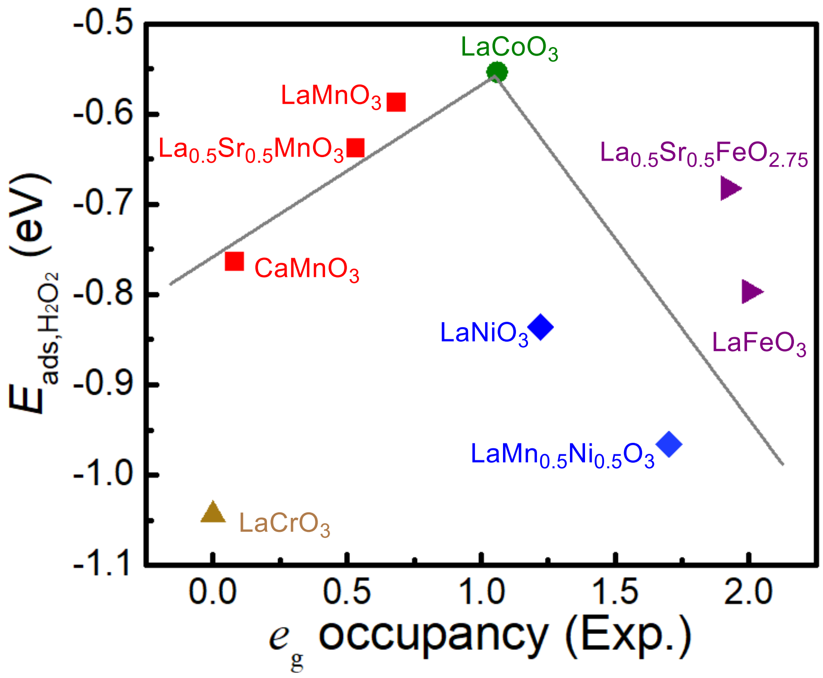

Supplementary Figure 24. The adsorption energies of H_2O_2 plotted as a function of e_g occupancy. Source data are provided as a Source Data file.

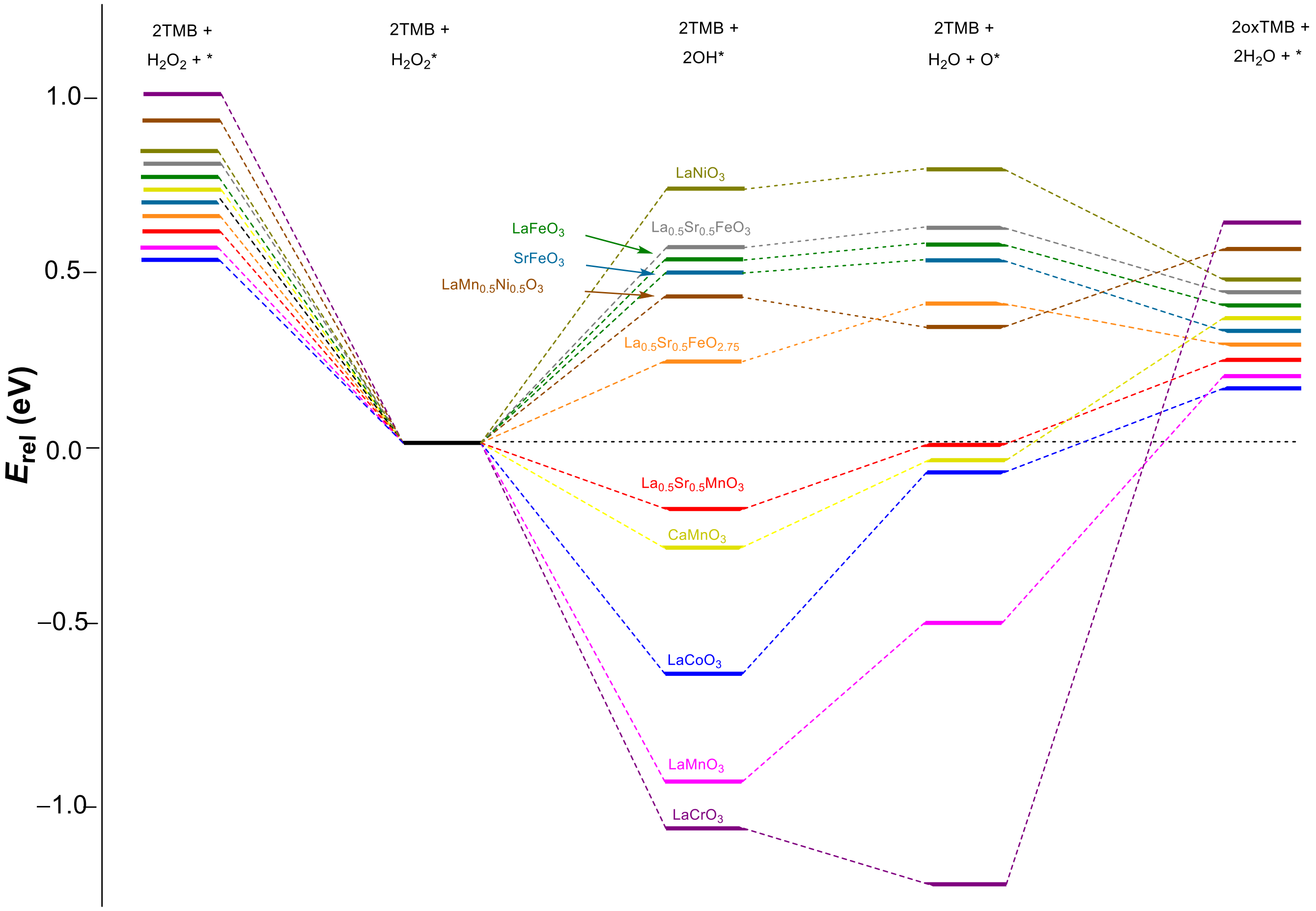

Supplementary Figure 25. Relative energies for intermediates involved in the catalytic reactions proposed in Figure 3a. Source data are provided as a Source Data file.

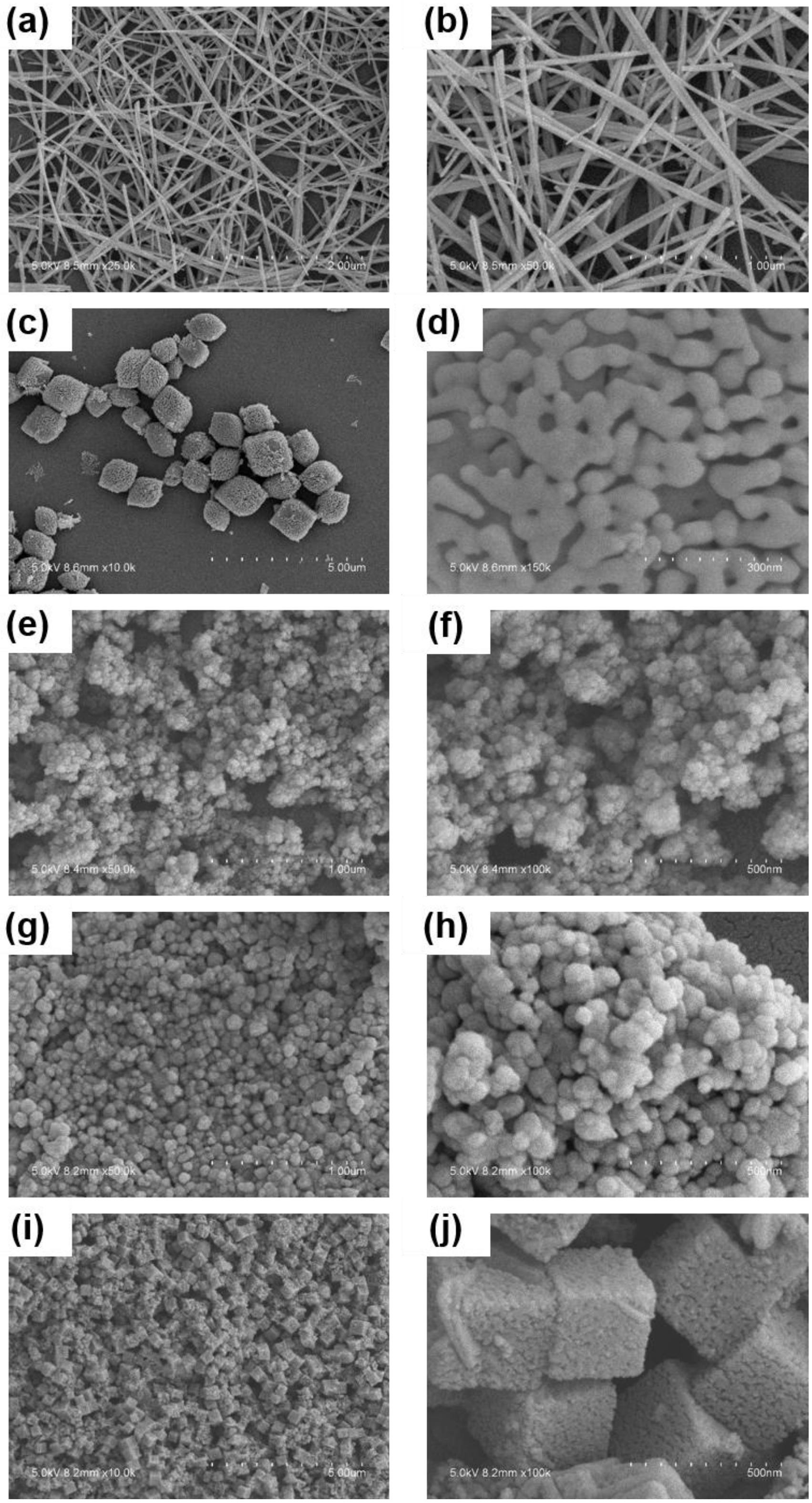

Supplementary Figure 26. Representative SEM images of binary TMOs. (**a**, **b**) MnO_2, (**c**, **d**) $Mn_2O_{3-\delta}$, (**e**, **f**) CoO, (**g**, **h**) NiO, and (**i**, **j**) Fe_2O_3. Source data are provided as a Source Data file.

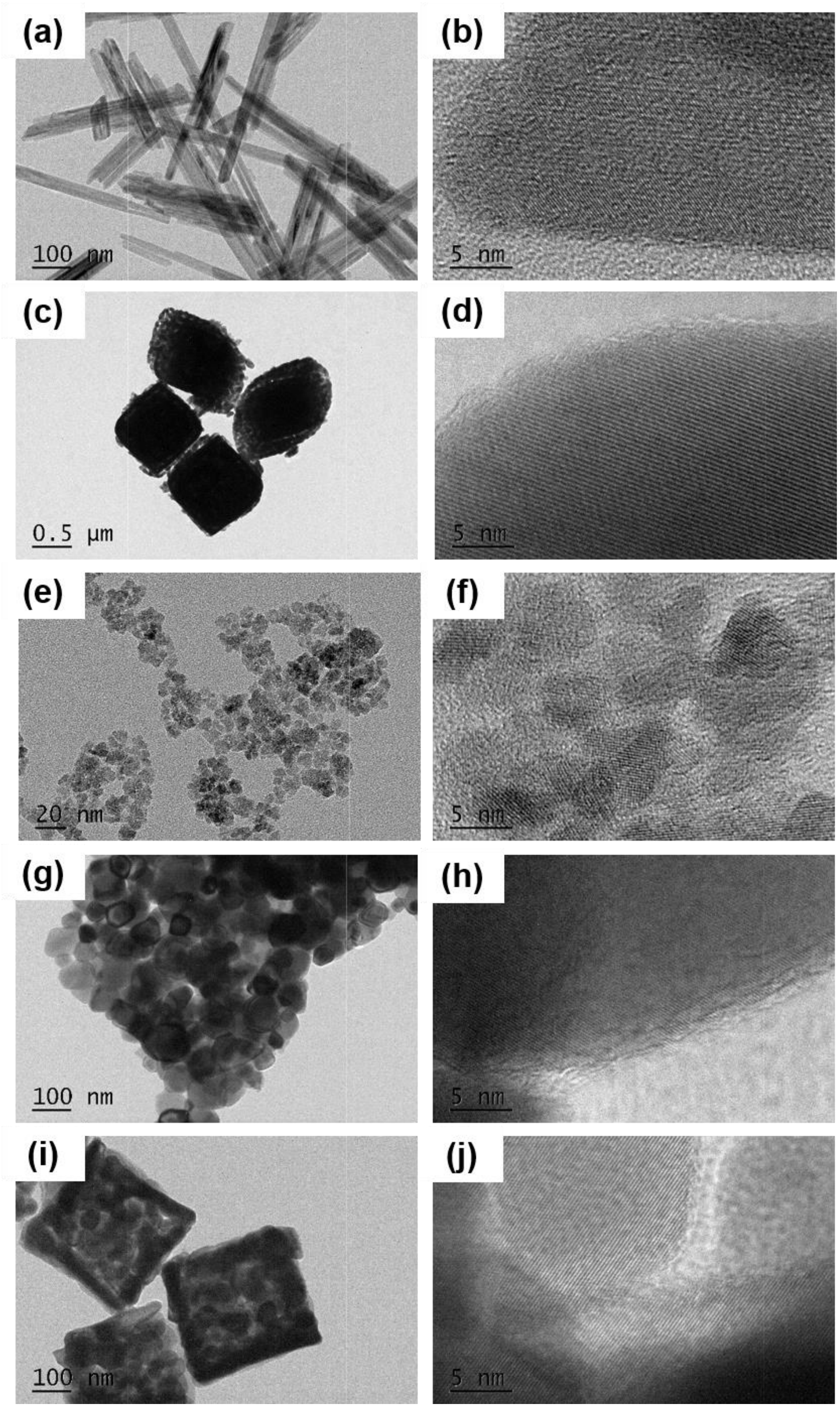

Supplementary Figure 27. Representative TEM images of binary TMOs. (**a**, **b**) MnO_2, (**c**, **d**) $Mn_2O_{3-\delta}$, (**e**, **f**) CoO, (**g**, **h**) NiO, and (**i**, **j**) Fe_2O_3. Source data are provided as a Source Data file.

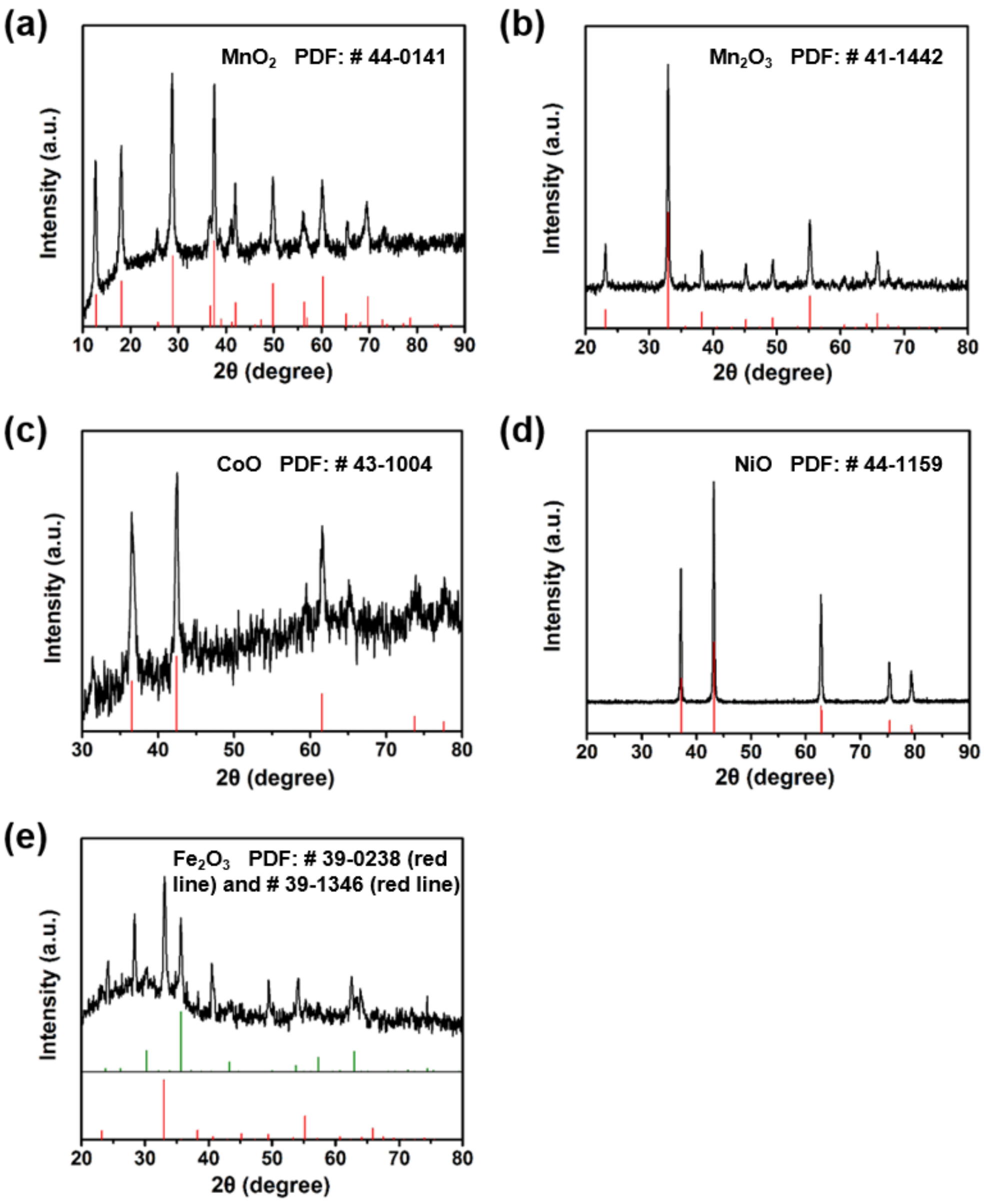

Supplementary Figure 28. PXRD patterns of (**a**) MnO_2, (**b**) $Mn_2O_{3-\delta}$, (**c**) CoO, (**d**) NiO, and (**e**) Fe_2O_3. Black curve: measured patterns; red and green curves: corresponding reference patterns from the JCPDS database (red and green curves in panel e for β-Fe_2O_3 and α-Fe_2O_3, respectively). Source data are provided as a Source Data file.

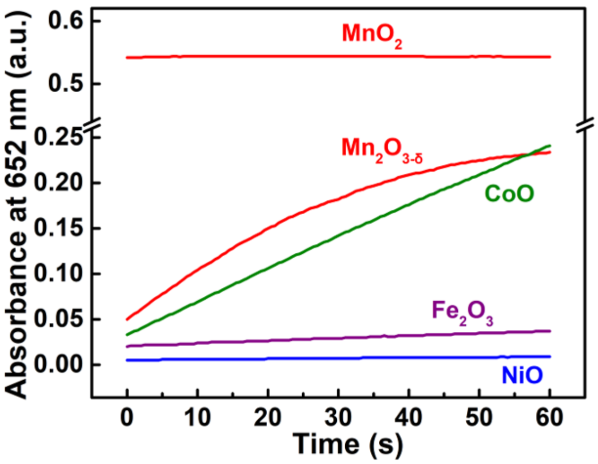

Supplementary Figure 29. Kinetic curves of A_{652} for monitoring the catalytic oxidation of 1 mM TMB with 100 mM H_2O_2 in the presence of 10 μg·mL^{-1} MnO_2, CoO, $Mn_2O_{3-\delta}$, NiO, and Fe_2O_3, respectively. Source data are provided as a Source Data file.

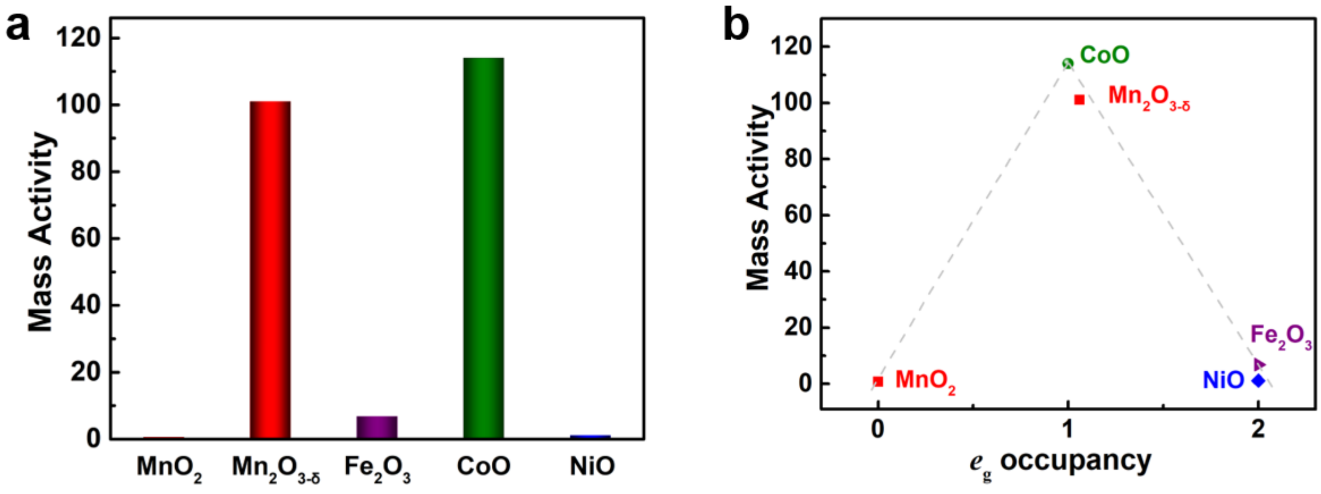

Supplementary Figure 30. (**a**) Mass normalized peroxidase-like activities of binary TMOs. (**b**) Mass normalized binary metal oxides' peroxidase-like activities as a function of e_g occupancy. Source data are provided as a Source Data file.

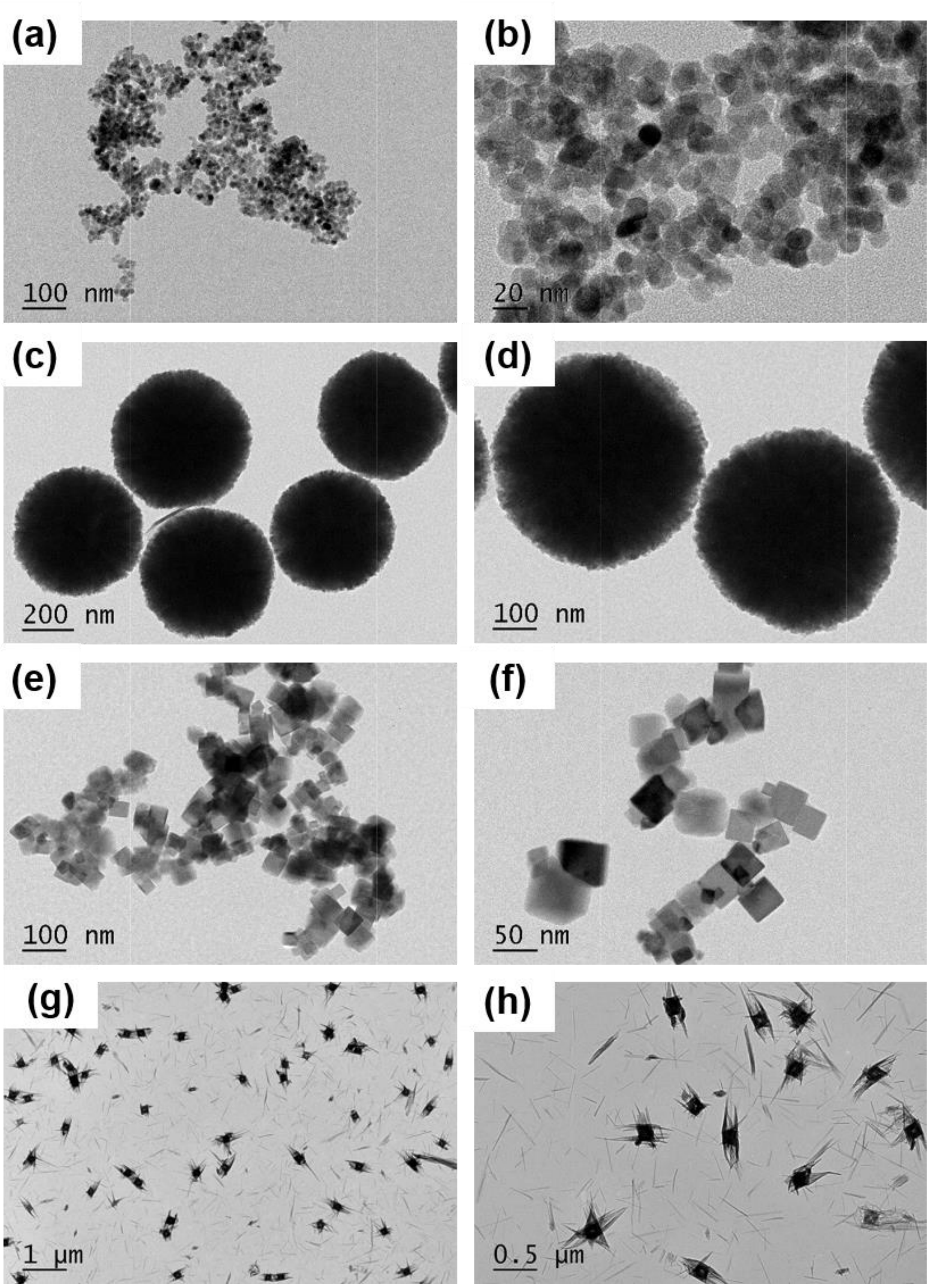

Supplementary Figure 31. Representative TEM images of other peroxidase mimics reported by references. (**a**, **b**) Mn_3O_4, (**c**, **d**) Fe_3O_4, (**e**, **f**) Co_3O_4, and (**g**, **h**) $Cu(OH)_2$. Source data are provided as a Source Data file.

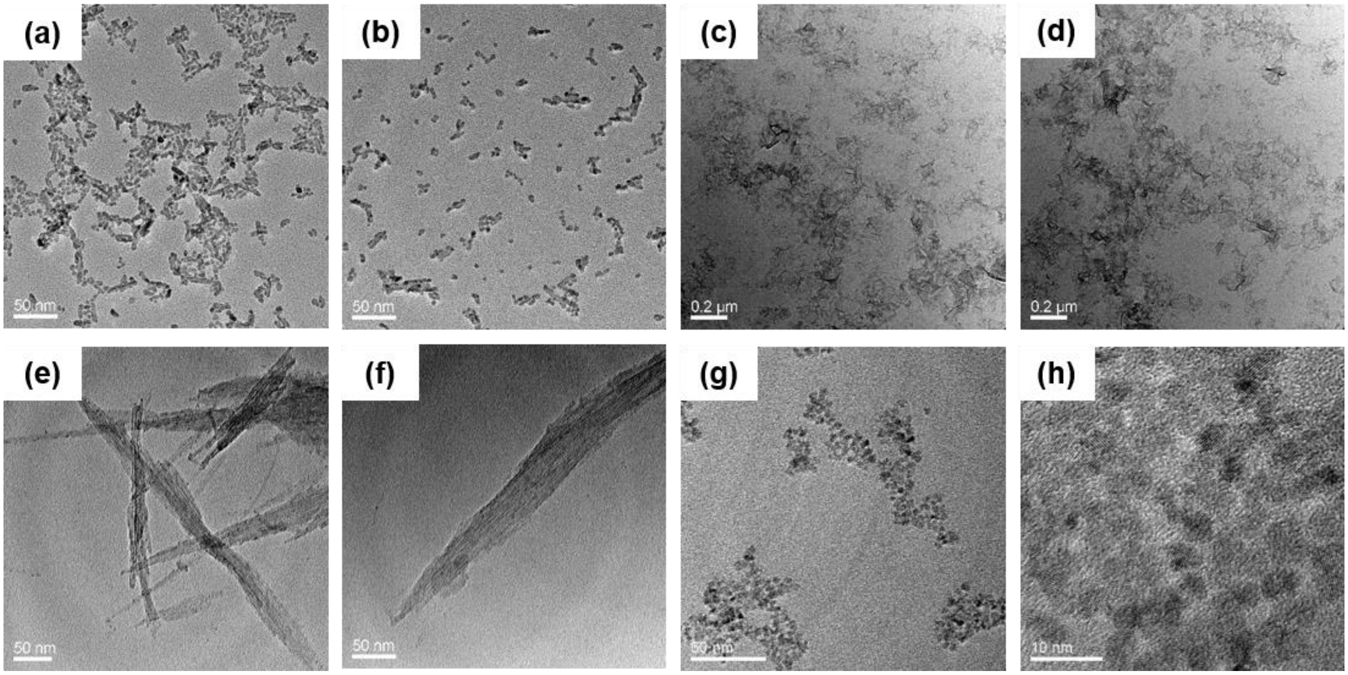

Supplementary Figure 32. Representative TEM images of other peroxidase mimics reported by references. (**a**, **b**) CuO, (**c**, **d**) GO-COOH, (**e**, **f**) SWNT, and (**g**, **h**) CeO_2. Source data are provided as a Source Data file.

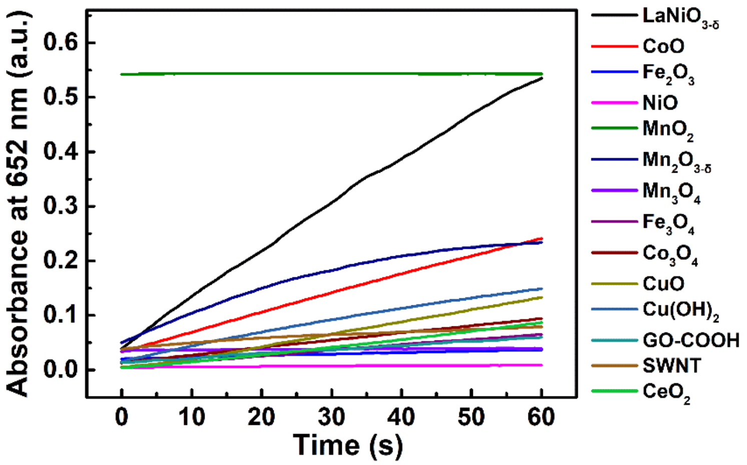

Supplementary Figure 33. Kinetic curves of A_{652} for monitoring the catalytic oxidation of 1 mM TMB with 100 mM H_2O_2 in the presence of 10 μg·mL^{-1} $LaNiO_3$ and other typical nanozymes reported by references. Source data are provided as a Source Data file.

Supplementary Table 1. BET surface area of the TMOs (including perovskite TMOs) studied in this work.

Oxide	BET surface area ($m^2 \cdot g^{-1}$)	Normalized BET surface area
**$LaNiO_3$**	17.06	1
**$LaNiO_3$-H_2**	12.11	0.71
**$LaMn_{0.5}Ni_{0.5}O_3$**	12.48	0.73
**$LaCoO_3$**	6.38	0.37
**$LaCrO_3$**	11.76	0.69
**$LaMnO_3$**	17.95	1.05
**$La_{0.5}Sr_{0.5}MnO_3$**	19.98	1.17
**$CaMnO_3$**	14.07	0.83
**$LaFeO_3$**	10.85	0.64
**$La_{0.5}Sr_{0.5}FeO_3$**	17.29	1.01
**$SrFeO_3$**	13.48	0.79
**MnO_2**	71.98	4.02
**Mn_2O_3**	18.16	1.06
**CoO**	28.658	1.68
**NiO**	8.73	0.51
**Fe_2O_3**	59.88	3.51

Supplementary Table 2. ICP-OES analysis of $LaMn_{0.5}Ni_{0.5}O_3$, $La_{0.5}Sr_{0.5}MnO_3$, and $La_{0.5}Sr_{0.5}FeO_3$.

Oxide	La	Sr	Cr	Ni	Mn	Cu	Fe
**$LaMn_{0.5}Ni_{0.5}O_3$**	46.5%			26.5%	27.0%		
**$La_{0.5}Sr_{0.5}MnO_3$**	24.4%	22.3%			53.3%		
**$La_{0.5}Sr_{0.5}FeO_3$**	24.8%	20.2%					55.0%

The ICP-OES analysis confirmed the successful synthesis of the doped perovskite TMOs with desired compositions.

Supplementary Table 3. Oxygen vacancy (δ), oxidation state of B site, and nominal e_g occupancy (standard deviations were obtained by three independent measurements).

Oxides	δ	Oxidation State	Nominal e_g
$LaNiO_{3-\delta}$	0.11 ± 0.01	2.77 ± 0.02	1.22 ± 0.02
$LaMn_{0.5}Ni_{0.5}O_{3-\delta}$	-0.004 ± 0.01	Ni: +2.3<br>Mn: +3.7	Ni: 1.7<br>Mn: 0.3
$LaCoO_{3-\delta}$	-0.03 ± 0.01	3.065±0.02	1.06 ± 0.02
$LaMnO_{3-\delta}$	-0.16 ± 0.006	3.32 ± 0.01	0.68 ± 0.01
$La_{0.5}Sr_{0.5}MnO_{3-\delta}$	0.015 ± 0.006	3.47 ± 0.01	0.53±0.01
$CaMnO_{3-\delta}$	0.038 ± 0.005	3.92 ± 0.01	0.08 ± 0.01
$La_{0.5}Sr_{0.5}FeO_{3-\delta}$	0.21 ± 0.006	3.08 ± 0.1	1.92 ± 0.02
$SrFeO_{3-\delta}$	0.34 ± 0.005	3.31 ± 0.01	1.69 ± 0.01
$Mn_2O_{3-\delta}$	0.05 ± 0.01	2.94 ± 0.01	1.06 ± 0.02

Note: the nominal e_g of $LaCoO_{3-\delta}$ in Figure 2b and Supplementary Figure 15 was obtained from magnetic measurements.

Supplementary Table 4. Transition metal's valence, spin state, and assignment of e_g occupancy for the TMOs (including perovskite TMOs).

Oxides	Valence	Spin State	Assignment	Reference
**$LaNiO_3$**	Ni^{3+}	L.S.	$t_{2g}^6e_g^1$	[1]
**$LaMn_{0.5}Ni_{0.5}O_3$**	$Ni^{2.3+}$	L.S.	$t_{2g}^6e_g^{1.7}$	[2]
	$Mn^{3.7+}$	H.S.	$t_{2g}^6e_g^{0.3}$	
**$LaCoO_3$**	Co^{3+}	I.S.	$t_{2g}^5e_g^1$	[3,4]
**$LaCrO_3$**	Cr^{3+}	N/A	$t_{2g}^3e_g^0$	[1]
**$LaMnO_3$**	Mn^{3+}	H.S.	$t_{2g}^3e_g^1$	[5]
**$La_{0.5}Sr_{0.5}MnO_3$**	$Mn^{3.5+}$	H.S.	$t_{2g}^3e_g^{0.5}$	[6]
**$CaMnO_3$**	Mn^{4+}	L.S.	$t_{2g}^3e_g^0$	[7,8]
**$LaFeO_3$**	Fe^{3+}	H.S.	$t_{2g}^3e_g^2$	[9]
**$La_{0.5}Sr_{0.5}FeO_3$**	$Fe^{3.5+}$	H.S.	$t_{2g}^3e_g^{1.5}$	[2,9]
**$SrFeO_3$**	Fe^{4+}	H.S.	$t_{2g}^6e_g^1$	[10]
**MnO_2**	Mn^{4+}	L.S.	$t_{2g}^3e_g^0$	[11]
**Mn_2O_3**	Mn^{3+}	H.S.	$t_{2g}^3e_g^1$	[12]
**CoO**	Co^{2+}	L.S.	$t_{2g}^6e_g^1$	[13]
**NiO**	Ni^{2+}	H.S.	$t_{2g}^6e_g^2$	[14]
**Fe_2O_3**	Fe^{3+}	H.S.	$t_{2g}^3e_g^2$	[15]

Note: H.S., high spin state; L.S., low spin state; I.S., intermediate spin state; N/A, not applicable.

Supplementary Table 5. BET surface area of other nanomaterials studied in this work.

Oxide	BET surface area ($m^2 \cdot g^{-1}$)	Normalized BET surface area
Mn_3O_4	88.43	5.18
Fe_3O_4	53.99	3.16
Co_3O_4	14.45	0.85
SWNT	703.95	41.26
CuO	101.23	5.93
CeO_2	714.51	41.88
$Cu(OH)_2$	61.456	3.60
GO-COOH	40.33	2.36

Supplementary Table 6. Effective magnetic moment (μ_{eff}) and e_g occupancy for different spin states of cobalt ion in $LaCoO_{3-\delta}$.

**Samples**				**$LaCoO_{3-\delta}$**
Spin state	LS	HS	50% HS + 50% LS	49.56% HS + 50.44% LS
μ_{eff} (μ_B)	0	4.90	3.46	3.45
e_g occupancy	e_g^0	e_g^2	e_g^1	$e_g^{0.99}$

Supplementary Table 7. Comparison of literature results and ours on the peroxidase-like activities of several binary TMOs.

Oxide	Diameter	Catalyst	H_2O_2	Substrate	Comments	Ref.
**NiO**	32 nm	H_2TCPP-NiO (40 μg·mL^{-1})	250 mM	TMB (0.5 mM)	The peroxidase-like activity could be mainly originated from the H_2TCPP on the NiO surface.	[16]
**NiO**	3-5 μm	NiO nanoflowers (190.5 μg·mL^{-1})	3.8 mM	TMB (95.2 μM)	In Figure 5, as high as 0.003 mol·mL^{-1} (225 mg·mL^{-1}) NiO was used.	[17]
**NiO**	100 nm	NiO nanoparticles (10 μg·mL^{-1})	50 mM	TMB (0.8 mM)		This work
**MnO_2**	4.5 nm	BSA-MnO_2 (3.7 μg·mL^{-1})	N/A	OPD (N/A)		[18]
**MnO_2**	30 nm in diameter	MnO_2 nanorod (10 μg·mL^{-1})	50 mM	TMB (0.8 mM)		This work
**Fe_2O_3**	9 nm	γ-Fe_2O_3 (20 μg·mL^{-1})	130 mM	TMB (0.25 mg·mL^{-1})	γ-Fe_2O_3 was coated by DMSA.	[19]
**Fe_2O_3**	20-50 nm	TemFe A (44.4 μg·mL^{-1})	4.44 M	TMB (1.11 mM)	The catalysis of TMB oxidation was attributed to Fe^{2+} on the surface of Fe_2O_3 which can react with H_2O_2 by Fenton reaction.	[20]
**Fe_2O_3**	350 nm	Fe_2O_3 cubes (10 μg·mL^{-1})	50 mM	TMB (0.8 mM)		This work

Note: Ref., Reference; N/A, not applicable; H_2TCPP, 5,10,15,20-tetrakis(4-carboxylpheyl)-porphyrin; DMSA, dimercaptosuccinic acid; TemFe, Template A prepared Fe_2O_3 nanoparticles; TMB, 3,3',5,5'-tetramethylbenzidine; OPD, *o*-phenylenediamine.

Supplementary Table 8. Kinetics parameters of TMOs as well as other representative nanozymes.

Catalyst	Substrate	K_m (mM)	υ_{max} (Ms^{-1})
**$SrFeO_{3-\delta}$**	H_2O_2	6.05	0.18×10^{-6}
**$La_{0.5}Sr_{0.5}MnO_{3-\delta}$**	H_2O_2	40.08	0.54×10^{-6}
**$La_{0.5}Sr_{0.5}FeO_{3-\delta}$**	H_2O_2	8.14	0.13×10^{-6}
**$LaNiO_{3-\delta}$**	H_2O_2	359.92	4.63×10^{-6}
**$LaNiO_{3-\delta}$-H_2**	H_2O_2	125.96	1.45×10^{-6}
**$LaMnO_{3-\delta}$**	H_2O_2	31.43	0.68×10^{-6}
**$LaMn_{0.5}Ni_{0.5}O_3$**	H_2O_2	112.84	0.44×10^{-6}
**CoO**	H_2O_2	92.10	1.14×10^{-6}
**$LaCoO_{3-\delta}$**	H_2O_2	205.45	0.93×10^{-6}
**SWNT**	H_2O_2	2.41	0.10×10^{-6}
**GO-COOH**	H_2O_2	23.66	0.11×10^{-6}
**CeO_2**	H_2O_2	4.41	0.18×10^{-6}
**Fe_2O_3**	H_2O_2	75.97	0.068×10^{-6}
**Fe_3O_4**	H_2O_2	41.66	0.16×10^{-6}
**CuO**	H_2O_2	31.18	0.28×10^{-6}
**Co_3O_4**	H_2O_2	41.75	0.26×10^{-6}
**Mn_2O_3**	H_2O_2	12.53	1.01×10^{-6}
**$Cu(OH)_2$**	H_2O_2	28.91	0.34×10^{-6}
**$LaCrO_3$**	H_2O_2	/	0.017×10^{-6}
**$LaFeO_3$**	H_2O_2	/	0.019×10^{-6}
**$CaMnO_{3-\delta}$**	H_2O_2	/	0.015×10^{-6}
**NiO**	H_2O_2	/	0.011×10^{-6}
**MnO_2**	H_2O_2	/	0.0064×10^{-6}
**Mn_3O_4**	H_2O_2	/	0.013×10^{-6}

Supplementary Table 9. The ground state magnetic orderings, space groups, and lattice parameters for ABO_3 perovskites obtained by calculations with the corresponding references.

Perovskite	Magnetic ordering	Space group	Lattice parameters	References
**$CaMnO_3$**	G-AFM	Pnma (62)	a = 5.412 Å; b =7.570 Å; c = 5.334 Å; α = β =γ = 90.0°	21,22
**$LaCrO_3$**	G-AFM	O-Pnma	a = 5.568 Å; b = 5.613 Å; c = 7.890 Å; α = β =γ = 90.0°	23-25
**$La_{0.5}Sr_{0.5}MnO_3$**	A-AFM	I-42M (121)	a = b = c = 5.556 Å; α = β = 120.7°; γ =88.7°	26,27
**$LaNiO_3$**	PM	R-R3c	a = b = c = 5.433 Å; α = β =γ = 61.4°	28-31
**$LaCoO_3$**	PM	R-R3c	a = b = c = 5.466 Å; α = β =γ = 60.9°	32,33
**$LaMnO_3$**	(HS : LS = 1 : 1)	O-Pnma	a = 5.816 Å; b = 7.820 Å; c = 5.583 Å; α = β =γ = 90.0°	25,34-37
**$SrFeO_3$**	A-AFM	Pm-3m (221)	a = b = c = 3.911 Å; α = β =γ = 90.0 °	38
**$SrFeO_{2.5}$**	PM	C1	a = 7.859 Å; b = 7.910 Å; c = 7.720 Å; α =γ= 90.0°; β = 90.1°	38
**$LaMn_{0.5}Ni_{0.5}O_3$**	(HS : LS = 1 : 1)	C2/M (12)	a = 9.561 Å; b = 5.551 Å; c = 7.816 Å; α =γ = 90.0°; β = 144.6°	39
**$La_{0.5}Sr_{0.5}FeO_3$**	PM	I-42M (121)	a = b = c = 5.557 Å; α = β = 120.5°; γ = 89.1°	40
**$La_{0.5}Sr_{0.5}FeO_{2.75}$**	(HS : LS = 1 : 1)	CM (8)	a = b = 5.513 Å; c = 11.114 Å; α = β = 119.7°; γ = 90.0°	40
**$LaFeO_3$**	A-AFM	O-Pnma	a = 5.667 Å; b = 7.945 Å; c = 5.601 Å; α = β =γ = 90.0°	23,25,40

Supplementary Table 10. Collection of the U_{eff} values for *d* orbitals of transition metals.

Element	Cr	Mn	Fe	Co	Ni
U_{eff}	3.7	3.9	5.3	3.32	6.2

Supplementary Notes

Supplementary Note 1. To further study the correlation between the e_g occupancy and their peroxidase-like activity, Ni-based perovskites were investigated. As shown in Supplementary Figure 10, $LaNiO_{3-\delta}$, with e_g occupancy of 1.22, showed a high catalytic activity. The $LaNiO_3$-H_2 composition was obtained by annealing $LaNiO_3$ nanoparticles at 350 ℃ for 2 h under a forming gas atmosphere of 5% H_2 in argon. The Ni^{3+} ions in the $LaNiO_{3-\delta}$ nanoparticles were partially reduced to Ni^{2+} upon annealing. As shown in Supplementary Figure 10b, the main PXRD peak of the $LaNiO_{3-\delta}$-H_2 nanoparticles shifted to slightly lower angles compared to that of the untreated $LaNiO_3$ nanoparticles, reflecting the lattice expansion due to the formation of larger Ni^{2+} cations. The activity of $LaNiO_{3-\delta}$-H_2, with e_g occupancy > 1.22, was approximately 44.1% of that of $LaNiO_{3-\delta}$. Moreover, $LaMn_{0.5}Ni_{0.5}O_3$ was formed by partial substitution of Ni by Mn in $LaNiO_3$. The introduction of Ni^{2+} in the $LaNiO_3$ lattice resulted in an effective e_g occupancy of 1.7 for Ni (Supplementary Figure 10f), and in a corresponding 7.71-fold decrease in activity compared to $LaNiO_{3-\delta}$. These results demonstrate that an e_g occupancy of 1.22 enhances the peroxidase-like activity of perovskites.

Supplementary Note 2.

***e_g* occupancy of Co^{3+} in $LaCoO_3$.** The exact of e_g of Co^{3+} in $LaCoO_3$ has been an ongoing debate.[41] For example, several previous studies showed that the cobalt ion in $LaCoO_3$ has intermediate spin with an e_g filling near unity.[1,2] Therefore, we measured the e_g of Co in our $LaCoO_{3-\delta}$ from the magnetization experiments with a MPMS SQUID magnetometer.[4] As shown in Supplementary Figure 14a, the temperature-dependent magnetization of $LaCoO_{3-\delta}$ was measured on a MPMS SQUID magnetometer (MPMS-3, Quantum Design) with a magnetic field of H = 1 kOe under field-cooling procedures to calculate the e_g occupancy of cobalt ions. The temperature-dependent susceptibilities can be obtained from magnetization data ($\chi = M/H$) (Supplementary Figure 14b). More, susceptibilities obey a paramagnetic Curie-Weiss law above 150 K, *i.e.*, $\chi = C/(T-\Theta)$, where C is Curie constant and can be obtained according to the fitting result in Supplementary Figure 14b. The effective magnetic moments (μ_{eff}) for $LaCoO_{3-\delta}$ was obtained by $\mu_{eff} = \sqrt{8C}\mu_B$. The spin state of cobalt ion in $LaCoO_{3-\delta}$ was controversial in past decades. Recent theoretical and experimental studies suggested that the mixture of low spin (LS) state and high spin (HS) state is more favorable. Here, we assumed the spin state of Co^{3+} in $LaCoO_{3-\delta}$ consists of LS state ($t_{2g}^6 e_g^0$, 0 μ_B) and HS state ($t_{2g}^4 e_g^2$, 4.9 μ_B). The fractions of Co ions in different spin states can be obtained from the relationship: $\mu_{eff} = g\mu_B\sqrt{S_{HS}(S_{HS}+1)\,V_{HS} + S_{LS}(S_{LS}+1)\,V_{LS}}$, where S_{HS} and S_{LS} are the S values, and V_{HS} and V_{LS} are the volume fraction for

Co ions in HS and LS states. The e_g electron occupancy (x) can be obtained by $x = S_{HS} * V_{HS} + S_{LS} * V_{LS}$. As shown in Supplementary Figure 14 and Supplementary Table 6, an e_g of 0.99 was obtained for our $LaCoO_{3-\delta}$. Therefore, the claim that the high peroxidase-like activity should be observed for e_g around 1 also holds true for $LaCoO_{3-\delta}$.

Supplementary Note 3.

Effect of B-O covalency on peroxidase-like activity. For $LaMnO_{3-\delta}$, $LaCoO_{3-\delta}$, and $LaNiO_{3-\delta}$ with the same A site and unit e_g occupancy shown in Supplementary Figure 19, their peroxidase-like activity follows the trend: $LaMnO_{3-\delta} < LaCoO_{3-\delta} < LaNiO_{3-\delta}$. To understand this trend, the dependence of the specific activity on the B-O covalency of $LaMnO_{3-\delta}$, $LaCoO_{3-\delta}$, and $LaNiO_{3-\delta}$ is plotted, showing a good correlation. Taken together, our results show that the peroxidase-like activity of perovskites is governed primarily by e_g occupancy and secondarily by B-O covalency. (Note, the B-O covalency values, quantified by the normalized O 1s → B 3d – O 2p absorbance from O K-edge X-ray absorption spectra.[2]) Moreover, the highest peroxidase-like activity observed for $LaNiO_{3-\delta}$ nanozyme could be understood by its unit e_g occupancy and strong B-O covalency.

Supplementary Note 4. Several studies reported the peroxidase-like activities of MnO_2, Fe_2O_3, and NiO. According our proposed design principles, however, neither of them should be active due to their e_g occupancy (i.e., MnO_2 (e_g=0), Fe_2O_3 (e_g=2), and NiO (e_g=2)). Indeed, our experimental results showed that all of the three nanozymes exhibited negligible peroxidase-like activities. This discrepancy can be attributed to several factors (see Supplementary Table 7). First, for most of the previous studies, the amounts of nanozymes, TMB, and H_2O_2 used were much higher than the current study. It would be reasonable to observe the nanomaterials mediated TMB oxidation under those conditions. Second, some of the previous studies used nanozyme with much smaller sizes, which would in turn enhance their peroxidase-like activities. Third, some of the peroxidase-like were from the other components rather than the nanomaterials themselves (for instance, the peroxidase-like activity of H_2TCPP-NiO was mainly from H_2TCPP rather than NiO). Moreover, the surface chemistry may also play an important role in the metal oxides' peroxidase-like activities (for example, Fe^{2+} on the surface of Fe_2O_3 may be responsible for the observed peroxidase-like activity of the Fe_2O_3 in the previous study).

Supplementary Note 5. In Supplementary Figures 15 and 30, the mass normalized peroxidase-like activities of TMOs were plotted versus e_g occupancy. While the BET surface area based specific activities reflected the nanozymes' intrinsic activities, the mass-based activities were also critical for practical applications since the nanozymes with high mass activities would

reduce the catalysts' cost and weight. As shown in Supplementary Figures 15 and 30, the overall mass activity trends of the TMOs based nanozymes were also governed by e_g electron occupancy. More, the e_g dependent mass normalized activities of TMOs exhibited a volcano-shaped relationship. As shown in Figures 2, 4 and Supplementary Figures 15 and 30, $LaNiO_{3-\delta}$ based nanozyme exhibited both the highest specific and mass activities, therefore it was the best nanozyme with peroxidase-like activity.

Supplementary Note 6. The lowest-energy structures and electronic configurations for perovskites have been extensively studied previously. Their ground state magnetic orderings vary from G-AFM, A-AFM, and PM (Supplementary Table 9). To consider all these possible spin alignments, we used a sufficiently large unit cell, $A_8B_8O_{24}$, for each defect-free perovskite bulk structure (Supplementary Figure 20). For $SrFeO_{2.5}$ and $La_{0.5}Sr_{0.5}FeO_{2.75}$ with oxygen vacancies, we used $A_8B_8O_{20}$ and $A_8B_8O_{22}$ unit cells, respectively (Supplementary Figure 20). The oxygen vacancies in each of these defect structures were kept to be separated from each other as far as possible. For geometry optimizations of bulks, their space group symmetries (Supplementary Table 9) were used to constrain the geometries. For geometry optimizations using slab models, lattice parameters and atoms in the bottom two layers (i.e., one AO and one BO_2 layer) were frozen, and the above atoms were allowed to move. For perovskites, all B sites in BO_2 terminated (001) facets are five coordinated in the form of BO_5, each of which has one open coordination site, analogous to metals in metalloporphyrins, i.e., active centers of many natural enzymes. These B sites are thus the most probable active centers for perovskites in catalysis. For this reason, we focused our attention on BO_2-terminated (001) facets to study the role of e_g occupancy in their peroxidase mimicking catalysis. All the (001) slab models were cut from the correspondingly relaxed perovskite bulk structures. Test calculations using the $LaNiO_3$ (001) slab model with an atomic layer 8 and a vacuum height 15 Å predicted an oxygen adsorption energy very close to that by the 6 atomic layer and 15 Å vacuum height model (–2.27 vs. –2.22 eV). Similarly, both models also predicted very close H_2O_2 adsorption energies (–0.95 vs. –0.91 eV). Therefore, for all slab models, their atomic layers and vacuum heights were set to 6 and 15 Å, respectively (Supplementary Figure 21).

Adsorption energies were calculated with the following equation,

$$E_{ads} = E_{slab+mol} - (E_{slab} + E_{mol})$$

where $E_{slab+mol}$ denotes the total energy of the interacting ABO_3 slab and adsorbate system, and E_{slab} and E_{mol} are the energies of the isolated slab and adsorbate, respectively. Because the energies of perovskite slabs were dependent on their magnetic orderings, attention was paid to tune the magnetic orderings when calculating the adsorption energies. Briefly, the initial magnetic moments and orderings of the perovskite (001) slabs were kept the same as those of the corresponding bulks. For slabs with adsorbents, only those for the adsorption sites were allowed to change. We found that the adsorption of a H_2O_2 molecule on the surfaces did not change the magnetic moments of the pristine surfaces significantly. The adsorption of an OH group on each B site reduced the magnetic moment of the site by about one unit, because of the antiferromagnetic coupling of the spins. The adsorption of an O atom on each B site either reduced or minimally changed the magnetic moment of the site.

Adsorption energies calculated using the above method did not include the contribution of zero-point energies (ZPEs) and entropies of the systems. However, most of these items would be cancelled out when relative E_{ads}'s were considered. For example, we performed frequency analyses for $LaNiO_3$ and $LaFeO_3$ (011) slabs and their O adsorption species, O@$LaNiO_3$ and O@$LaFeO_3$, to obtain their ZPEs and entropies, using the Phonopy code. No atoms were frozen in these calculations. The obtained ZPEs for these four species were 2.9, 3.1, 3.1, and 3.3 eV, respectively, and the entropies were 1.4, 1.3, 1.0, and 1.1 kJ $\cdot mol^{-1} \cdot K^{-1}$, respectively. The $E_{ads,O}$'s on $LaNiO_3$ and $LaFeO_3$ (001) surfaces before ZPE and entropy corrections were −1.7 and −1.8 eV, respectively. After the corrections, they became −0.8 and −0.8 eV, respectively. The differences of $LaNiO_3$ and $LaFeO_3$ in E_{ads}'s before and after the correction were both within 0.1 eV. These calculations verified that ZPE and entropy corrections little influenced the relative order of the adsorption energies.

The O p-band centers of perovskite bulks were calculated as the centroid of the densities of states projected onto the $2p$ orbitals of the O atoms using the following equation

$$\overline{\mathrm{O}}_{2p} = \frac{\int_{-\infty}^{\infty} E \cdot D_{\mathrm{O}_{2p}}(E)\ dE}{\int_{-\infty}^{\infty} D_{\mathrm{O}_{2p}}(E)\ dE} - E_{\mathrm{Fermi}}$$

where $\overline{\mathrm{O}}_{2p}$ is the O p-band center, E is the electron energy, $D_{\mathrm{O}_{2p}}(E)$ is the DOS projected onto the $2p$ orbitals of O, and the E_{Fermi} is the Fermi energy. The integrals were taken over all states, not just filled states.

Supplementary Methods

Chemicals. All metal nitrate salts, potassium permanganate, cobalt sulfate heptahydrate, manganese sulfate monohydrate, citric acid, hydrogen peroxide, and glucose were obtained from Nanjing Chemical Reagent Co., Ltd. Ethylene glycol and hydrochloric acid were purchased from Sinopharm Chemical Reagent Co., Ltd. TMB (3,3',5,5'-tetramethylbenzidine) and other chemicals were purchased from Aladdin Chemical Reagent Co., Ltd. All chemical reagents were used as received without further purification. Deionized water produced by Millipore system was used in all experiments.

Instrumentation. Inductively coupled plasma-optical emission spectroscopy (ICP-OES) measurements were performed on an Optima 5300DV analyzer.

Synthesis of $LaNiO_3$-H_2. The $LaNiO_3$-H_2 was obtained by controlled reduction of as-prepared $LaNiO_3$ with H_2. Specifically, the as-prepared $LaNiO_3$ was heated to 350 ℃ at a ramp rate of 5 ℃·min^{-1} and maintained at 350 ℃ for 2 h under a forming gas of 5% H_2 in Argon.

Synthesis of MnO_2. $KMnO_4$ (1.94 g in 20 mL H_2O) and $MnSO_4 \cdot H_2O$ (0.845 g in 5 mL H_2O) were added into a Teflon-lined autoclave and heated at 160 ℃ for 12 h. The resulting product was centrifuged and washed with H_2O for several times. The obtained MnO_2 was dried at 60 ℃ for overnight.[42]

Synthesis of $Mn_2O_{3-\delta}$. 0.474 g $KMnO_4$ and 0.595 g glucose were dissolved in 10 mL H_2O, respectively. The glucose solution was added dropwise to the $KMnO_4$ solution under stirring and the mixed solution was further stirred for half an h. The resulting solution was then transferred to a Teflon-lined autoclave and heated at 150 ℃ for 10 h. The resulting product was centrifuged and washed with H_2O for several times, which was then dried at 60 ℃ for overnight. The solid powder was finally annealed at 550 ℃ for 4 h with a ramp rate of 5 ℃·min^{-1} to obtain final $Mn_2O_{3-\delta}$.[42]

Synthesis of CoO. 0.14 g $CoSO_4 \cdot 7H_2O$ and 0.3 g urea were dissolved in 6.25 mL ethanol and 41.25 mL H_2O mixed solution under stirring, followed by adding 4 mL ammonia solution (28%) dropwise. After stirring for 30 min, the resulting solution was transferred into a Teflon-lined autoclave and heated at 120 ℃ for 12 h. The resulting product was centrifuged and washed with H_2O for several times, which was then dried at 60 ℃ for overnight. The solid powder was finally annealed at 450 ℃ in Argon for 3 h to obtain CoO nanoparticles.[43]

Synthesis of Fe_2O_3. PVP (polyvinylpyrrolidone, K30, 3.8 g) and $K_4Fe(CN)_6 \cdot 3H_2O$ (0.11 g) were added to a HCl solution (0.1 M, 50 mL) under stirring. After stirring for 30 min, the solution was heated to 80 ℃ and maintained at 80 ℃ for 20 h to form Prussian blue product. The resulting product was washed for several times with H_2O and absolute ethanol, which was then dried at 25 ℃ in a vacuum for overnight. The as-prepared Prussian blue was heated at 550 ℃ for 6 h with a ramp rate of 2 ℃·min^{-1} to obtain the final Fe_2O_3 particles.[44]

Synthesis of NiO. 0.5 g NaOH and 1.66 g PVP (K30) were dissolved in 25 mL H_2O and 1.45 g $Ni(NO_3)_2 \cdot 6H_2O$ was dissolved in 10 mL H_2O, respectively. The nickel nitrate aqueous solution was then added to the NaOH/PVP solution dropwise under stirring. The resulting solution was stirred for 3 h to obtain the NiO precursor, followed by washing with H_2O and ethanol for several times and drying at 60 ℃ for overnight. Then, the precursor was calcinated at 650 ℃ for 2 h in air with a temperature ramp rate of 5 ℃·min^{-1} to form the final NiO nanoparticles.[45]

Synthesis of Mn_3O_4. An 80% hydrazine solution (5 mL) was added to an aqueous solution of 0.2 M $KMnO_4$ (20 mL) under stirring and the mixed solution was further stirred for 12 h. The resulting product was centrifuged and washed with H_2O for several times, which was then dried at 110 ℃ for overnight.[42]

Synthesis of Fe_3O_4. 1.35 g $FeCl_3$ was dissolved in 40 mL ethylene glycol, followed by the addition of 3.6 g NaOAc under stirring. The resulting solution was then transferred to a Teflon-lined autoclave and heated at 200 ℃ for 6 h. The resulting product was centrifuged and washed with ethanol for several times, which was then dried at 60 ℃ for overnight.[46]

Synthesis of Co_3O_4. 2 g $Co(CH_3COO)_2{\cdot}4H_2O$ was dissolved in 25 mL H_2O, followed by adding 2.5 mL ammonia solution (28%) dropwise under stirring and the mixed solution was further stirred for half an h. The resulting solution was then transferred to a Teflon-lined autoclave and heated at 150 ℃ for 3 h. The resulting product was centrifuged and washed with H_2O for several times, which was then dried at 60 ℃ for overnight.[47]

Synthesis of CuO. 0.02 M $Cu(CH_3COO)_2{\cdot}H_2O$ (75 mL) was mixed with 0.25 mL CH_3COOH in a flask equipped with a refluxing device. The resulting solution was heated to boiling, followed by the addition of 0.04 $g{\cdot}mL^{-1}$ NaOH (5 mL) under stirring. Then, the mixed solution was further stirred for 5 min. The resulting product was centrifuged and washed with H_2O for several times, which was then dried at 60 ℃ for overnight.[48]

Synthesis of $Cu(OH)_2$. 17 mg $CuCl_2{\cdot}2H_2O$ and 130.5 mg PVP (molecular weight = 55000) were dissolved in 40 mL ethanol in a round-bottomed flask. The mixed solution was ultrasonicated for 10 min and stirred for 30 min. Then, 7.7 mg $NaBH_4$ was dissolved in 10 mL of ethanol and quickly added to the above solution under stirring. After stirring for 72 h, the resulting product was centrifuged and washed with ethanol for several times to obtain $Cu(OH)_2$ nanoparticles. To synthesis $Cu(OH)_2$ supercages, the $Cu(OH)_2$ nanoparticles and 20 mg PVP (molecular weight = 1300000) were dispersed in 20 mL of deionized water by ultrasonication for 10 min in a round-bottomed flask. A copper-ammonia complex solution was prepared by mixing 24.16 mg $Cu(NO_3)_2{\cdot}3H_2O$ and 0.4 mL $NH_3{\cdot}H_2O$ (28%) in 19.6 mL H_2O. Subsequently, the copper-ammonia complex solution was added to the above $Cu(OH)_2$ nanoparticles solution under stirring and the mixed solution was further stirred for 10 min. The resulting product was centrifuged and washed with methanol for several times, which was then dried at 60 ℃ for overnight.[49]

Synthesis of GO-COOH. 40 mg GO (graphene oxide) was added into 20 mL H_2O, and then the GO aqueous solution was sonicated for 2 h to obtain a clear solution. Subsequently, 4.8 g NaOH and 4 g chloroacetic acid were added into the above GO solution and sonicated for 3 h. The resulting solution was centrifuged and washed with H_2O for several times, which was then dried at 60 ℃ for overnight.[50]

Synthesis of SWNT. 50 mg SWNT (single-walled carbon nanotubes) was added into the mixture of 98% sulfuric acid and 70% nitric acid (3:1), and then the resulting solution was sonicated for 24 h. After sonication, the resulting product was centrifuged and washed with H_2O for several times, which was then dried at 60 ℃ for overnight.[51]

Synthesis of CeO_2. 2.52 g cerium nitrate was dissolved in 100 mL of water-ethylene glycol mixture (1:1). After the temperature of the solution reached 60 ℃, 16 mL of $NH_3{\cdot}H_2O$ (28%) was rapidly added and the resulting mixture was vigorously stirred at 60 ℃ for 3 h until a yellow-colored dispersion was obtained. The reaction mixture was cooled and centrifuged to obtain yellow CeO_2 precipitate, which was washed multiple times with ethanol and water till the pH was neutral. The obtained CeO_2

precipitate was further dispersed in 100 mL of distilled water containing 30 mg·mL^{-1} sodium citrate. After sonication for 30 min, a transparent solution was obtained. Then, ethanol was added into above solution to assist the precipitation of citrate-stabilized nanoceria. The precipitate was washed with ethanol for several times to remove residual citrate. Finally, the nanoceria were redispersed into distilled water to obtain a highly transparent solution.[52]

Oxygen vacancies of TMOs. To quantify the oxidation state of TMOs, iodometric titrations were performed according to the referenced procedure.[53] The amount of oxygen vacancies δ in metal oxides were deduced by the oxidation state of TMOs. Briefly, 3 mL of 2 M KI solution and given qualities of TMOs were dissolved in a N_2-saturated HCl aqueous solution (1 M) under N_2 atmosphere and allowed to disperse and dissolve for five minutes. Then, the solution was titrated to a faint golden color with 0.1 M of $Na_2S_2O_3$ aqueous solution. Towards the end point, 0.5 mL starch indicator is added and the solution was titrated until clear. The titration was repeated three times for each oxide, from which an average oxidation state, oxygen vacancy and standard error were obtained.

Magnetic measurement of $LaCoO_3$. The temperature-dependent magnetization of $LaCoO_3$ was measured on a MPMS SQUID magnetometer (MPMS-3, Quantum Design) with a magnetic field of H = 1 kOe under field-cooling procedures to calculate the e_g occupancy of cobalt ions.

Supplementary References

1 Suntivich, J., May, K. J., Gasteiger, H. A., Goodenough, J. B. & Shao-Horn, Y. A perovskite oxide optimized for oxygen evolution catalysis from molecular orbital principles. *Science* **334**, 1383-1385 (2011).

2 Suntivich, J. *et al.* Design principles for oxygen-reduction activity on perovskite oxide catalysts for fuel cells and metal-air batteries. *Nat. Chem.* **3**, 546-550 (2011).

3 Yan, J. Q., Zhou, J. S. & Goodenough, J. B. Bond-length fluctuations and the spin-state transition in $LCoO_3$ (L=La, Pr, and Nd). *Phys. Rev. B* **69** (2004).

4 Zhou, S. *et al.* Engineering electrocatalytic activity in nanosized perovskite cobaltite through surface spin-state transition. *Nat. Commun.* **7**, 11510 (2016).

5 Ritter, C. *et al.* Influence of oxygen content on the structural, magnetotransport, and magnetic properties of $LaMnO_{3+\delta}$. *Phys. Rev. B* **56**, 8902-8911 (1997).

6 Medvedeva, J. E., Anisimov, V. I., Mryasov, O. N. & Freeman, A. J. The role of Coulomb correlation in magnetic and transport properties of doped manganites: $La_{0.5}Sr_{0.5}MnO_3$ and $LaSr_2Mn_2O_7$. *J. Phys.: Condens. Matter* **14**, 4533-4542 (2002).

7 Kim, J., Yin, X., Tsao, K.-C., Fang, S. & Yang, H. $Ca_2Mn_2O_5$ as Oxygen-Deficient Perovskite Electrocatalyst for Oxygen Evolution Reaction. *J. Am. Chem. Soc.* **136**, 14646-14649 (2014).

8 Guo, Y. *et al.* Engineering the electronic state of a perovskite electrocatalyst for synergistically enhanced oxygen evolution reaction. *Adv. Mater.* **27**, 5989-5994 (2015).

9 Russo, U., Nodari, L., Faticanti, M., Kuncser, V. & Filoti, G. Local interactions and electronic phenomena in substituted $LaFeO_3$ perovskites. *Solid State Ionics* **176**, 97-102 (2005).

10 Kawasaki, S., Takano, M. & Takeda, Y. Ferromagnetic properties of $SrFe_{1-x}Co_xO_3$ synthesized under high pressure. *J. Solid State Chem.* **121**, 174-180 (1996).

11 Lee, S. *et al.* Enhanced intrinsic catalytic activity of λ-MnO_2 by electrochemical tuning and oxygen vacancy generation. *Angew. Chem.-Int. Edit.* **55**, 8599-8604 (2016).

12 Maitra, U., Naidu, B. S., Govindaraj, A. & Rao, C. N. R. Importance of trivalency and the e_g^1 configuration in the photocatalytic oxidation of water by Mn and Co oxides. *Proc. Natl. Acad. Sci. USA* **110**, 11704-11707 (2013).

13 Liu, Y. W. *et al.* Low overpotential in vacancy-rich ultrathin $CoSe_2$ nanosheets for water oxidation. *J. Am. Chem. Soc.* **136**, 15670-15675 (2014).

14 Zhao, Y. *et al.* Ultrafine NiO nanosheets stabilized by TiO_2 from monolayer NiTi-LDH precursors: An active water oxidation electrocatalyst. *J. Am. Chem. Soc.* **138**, 6517-6524 (2016).

15 Pozun, Z. D. & Henkelman, G. Hybrid density functional theory band structure engineering in hematite. *J. Chem. Phys.* **134**, 224706 (2011).

16 Liu, Q. *et al.* NiO nanoparticles modified with 5,10,15,20-tetrakis(4-carboxyl pheyl)-porphyrin: Promising peroxidase mimetics for H_2O_2 and glucose detection. *Biosens. Bioelectron.* **64**, 147-153 (2015).

17 Ray, C. *et al.* Intrinsic peroxidase-like activity of mesoporous nickel oxide for selective cysteine sensing. *J. Mater. Chem. B* **2**, 6097-6105 (2014).

18 Liu, X. *et al.* BSA-templated MnO_2 nanoparticles as both peroxidase and oxidase mimics. *Analyst* **137**, 4552-4558 (2012).

19 Chen, Z. *et al.* Dual enzyme-like activities of iron oxide nanoparticles and their implication for diminishing cytotoxicity. *ACS Nano* **6**, 4001-4012 (2012).

20 Roy, A., Sahoo, R., Ray, C., Dutta, S. & Pal, T. Soft template induced phase selective synthesis of Fe_2O_3 nanomagnets: one step towards peroxidase-mimic activity allowing colorimetric sensing of thioglycolic acid. *RSC Adv.* **6**, 32308-32318 (2016).

21 Bhattacharjee, S., Bousquet, E. & Ghosez, P. First-principles study of the dielectric and dynamical properties of orthorhombic $CaMnO_3$. *J. Phys.: Condens. Matter* **20**, 255229 (2008).

22 Wang, K., Jiang, W., Chen, J.-N. & Huang, J.-Q. Study of the electronic structure and half-metallicity of $CaMnO_3/BaTiO_3$ superlattice. *Superlattice Microst* **97**, 116-124 (2016).

23 Zhang, Q., Yunoki, S. & Iop. A first-principles study for electronic and magnetic properties of $LaFeO_3/LaCrO_3$ superlattices. *J. Phys.: Conf. Ser.* **400**, 032126 (2012).

24 Dabaghmanesh, S., Sarmadian, N., Neyts, E. C. & Partoens, B. A first principles study of p-type defects in $LaCrO_3$. *Phys. Chem. Chem. Phys.* **19**, 22870-22876 (2017).

25 Mahadevan, P., Shanthi, N. & Sarma, D. D. Electronic structure of $LaCrO_3$, $LaMnO_3$ and $LaFeO_3$ from ab initio spin-polarized calculations. *J. Phys.: Condens. Matter* **9**, 3129-3138 (1997).

26 Nguyen, T. T. & Bach, T. C. Strain effects on multiferroic heterointerface $La_{0.5}Sr_{0.5}MnO_3/BaTiO_3$ by DFT calculations. *Adv. Nat. Sci: Nanosci. Nanotechnol.* **9**, 015012 (2018).

27 Radaelli, P. G., Cox, D. E., Marezio, M. & Cheong, S. W. Charge, orbital, and magnetic ordering in $La_{0.5}Ca_{0.5}MnO_3$. *Phys. Rev. B* **55**, 3015-3023 (1997).

28 Guan, L. *et al.* Electronic structure and optical properties of $LaNiO_3$: First-principles calculations. *Solid State Commun.* **150**, 2011-2014 (2010).

29 Pan, C.-C. *et al.* First-principle study of O vacancy on $LaNiO_3$ (001) surface. *Int. J. Hydrogen Energ.* **41**, 15756-15763 (2016).

30 Malashevich, A. & Ismail-Beigi, S. First-principles study of oxygen-deficient $LaNiO_3$ structures. *Phys. Rev. B* **92**, 144102 (2015).

31 Pan, C. *et al.* A First Principles Study of H_2 Adsorption on $LaNiO_3$(001) Surfaces. *Materials* **10**, 36 (2017).

32 Laref, A. & Sekkal, W. First-principle calculations of anomalous spin-state excitation in $LaCoO_3$. *Mater. Chem. Phys.* **123**, 125-131 (2010).

33 Hsu, H., Umemoto, K., Cococcioni, M. & Wentzcovitch, R. First-principles study for low-spin $LaCoO_3$ with a structurally consistent Hubbard *U. Phys. Rev. B* **79**, 125124 (2009).

34 Rivero, P., Meunier, V. & Shelton, W. Electronic, structural, and magnetic properties of $LaMnO_3$ phase transition at high temperature. *Phys. Rev. B* **93**, 024111 (2016).

35 Abuova, A. U. *et al.* First principles modeling of Ag adsorption on the $LaMnO_3$ (001) surfaces. *Solid State Ionics* **273**, 46-50 (2015).

36 Pilania, G. & Ramprasad, R. Adsorption of atomic oxygen on cubic $PbTiO_3$ and $LaMnO_3$ (001) surfaces: A density functional theory study. *Surf. Sci.* **604**, 1889-1893 (2010).

37 Satpathy, S., Popovic, Z. S. & Vukajlovic, F. R. Electronic structure of the perovskite oxides: $La_{1-x}Ca_xMnO_3$. *Phys. Rev. Lett.* **76**, 960-963 (1996).

38 Berger, R. F., Broberg, D. P. & Neaton, J. B. Tuning the electronic structure of $SrTiO_3/SrFeO_{3-x}$ superlattices via composition and vacancy control. *APL Mater.* **2**, 046101 (2014).

39 Joly, V. L. J., Joy, P. A., Date, S. K. & Gopinath, C. S. Two ferromagnetic phases with different spin states of Mn and Ni in $LaMn_{0.5}Ni_{0.5}O_3$. *Phys. Rev. B* **65**, 184416 (2002).

40 Ritzmann, A. M., Munoz-Garcia, A. B., Pavone, M., Keith, J. A. & Carter, E. A. Ab Initio DFT + U Analysis of Oxygen Vacancy Formation and Migration in $La_{1-x}Sr_xFeO_{3-\delta}$ (x=0, 0.25, 0.50). *Chem. Mater.* **25**, 3011-3019 (2013).

41 Hwang, J. *et al.* Perovskites in catalysis and electrocatalysis. *Science* **358**, 751-756 (2017).

42 Pokhrel, R., Goetz, M. K., Shaner, S. E., Wu, X. & Stahl, S. S. The "Best Catalyst" for water oxidation depends on the oxidation method employed: A case study of manganese oxides. *J. Am. Chem. Soc.* **137**, 8384-8387 (2015).

43 Jiang, Z.-J. & Jiang, Z. Interaction induced high catalytic activities of CoO nanoparticles grown on nitrogen-doped hollow graphene microspheres for oxygen reduction and evolution reactions. *Sci. Rep.* **6**, 27081 (2016).

44 Zhang, L., Wu, H. B., Madhavi, S., Hng, H. H. & Lou, X. W. Formation of Fe_2O_3 Microboxes with Hierarchical Shell Structures from Metal-Organic Frameworks and Their Lithium Storage Properties. *J. Am. Chem. Soc.* **134**, 17388-17391 (2012).

45 Mahaleh, Y. B. M., Sadrnezhaad, S. K. & Hosseini, D. NiO nanoparticles synthesis by chemical precipitation and effect of applied surfactant on distribution of particle size. *J. Nanomater.* **2008**, 470595 (2008).

46 Deng, H. *et al.* Monodisperse magnetic single-crystal ferrite microspheres. *Angew. Chem.-Int. Edit.* **44**, 2782-2785 (2005).

47 Dong, Y., He, K., Yin, L. & Zhang, A. A facile route to controlled synthesis of Co_3O_4 nanoparticles and their environmental catalytic properties. *Nanotechnology* **18**, 435602 (2007).

48 Chen, W. *et al.* Peroxidase-like activity of water-soluble cupric oxide nanoparticles and its analytical application for detection of hydrogen peroxide and glucose. *Analyst* **137**, 1706-1712 (2012).

49 Cai, R. *et al.* Single nanoparticle to 3D supercage: Framing for an artificial enzyme system. *J. Am. Chem. Soc.* **137**, 13957-13963 (2015).

50 Sun, X. *et al.* Nano-graphene oxide for cellular imaging and drug delivery. *Nano Res.* **1**, 203-212 (2008).

51 Song, Y. *et al.* Label-free colorimetric detection of single nucleotide polymorphism by using single-walled carbon nanotube intrinsic peroxidase-like activity. *Chem. Euro. J.* **16**, 3617-3621 (2010).

52 Muhammad, F., Wang, A., Qi, W., Zhang, S. & Zhu, G. Intracellular antioxidants dissolve man-made antioxidant nanoparticles: Using redox vulnerability of nanoceria to develop a responsive drug delivery system. *ACS Appl. Mater. Interfaces* **6**, 19424-19433 (2014).

53 Grimaud, A. *et al.* Double perovskites as a family of highly active catalysts for oxygen evolution in alkaline solution. *Nat. Commun.* **4**, 2439 (2013).
