# Supplementary material for: eg occupancy as an effective descriptor for the catalytic activity of perovskite oxide-based peroxidase mimics
Source: Nat Commun. 2019 Feb 11;10:704. doi: 10.1038/s41467-019-08657-5 (PMC6370761; doi:10.1038/s41467-019-08657-5)
Supplement: Supplementary file 2 — Peer Review File [file 41467_2019_8657_MOESM2_ESM.pdf]

Editorial Note: This manuscript has been previously reviewed at another journal that is not operating a transparent peer review scheme. This document only contains reviewer comments and rebuttal letters for versions considered at *Nature Communications.*

Reviewers' comments:

Reviewer #1 (Remarks to the Author):

The revised version of the paper "eg occupancy as an effective descriptor for the catalytic activity of perovskite oxide-based peroxidase mimics" has in general satisfyingly addressed my concerns. The authors now provide more detailed investigation and explanation of the relationship between the eg occupancy and superior POD-like nanozyme of perovskite oxide. They convincingly disclose the catalytic mechanism, by succeeded in providing more solid experimental evidence and theoretical discussions. The paper is well organized including writing. I thus recommend to publish the paper in the present form without alternation in Nature Communication.

Reviewer #2 (Remarks to the Author):
[Redacted]
The idea of providing a valid descriptor to predict the reactivity of these catalysts is sound and a plot as that of Figure 2b represents an excellent starting point for introducing this concept. The comparison with other descriptors that fail to correlate with the extensive set of data collected by the authors is also a good indication that eg occupancy is indeed a good parameter to take into consideration.

There are a number of things that have not been addressed yet in spite of the work the authors have done to increase the quality of their contribution in response to the first round of reviews.

1) As a physical organic chemist, I was struck by the poor (or missing) analysis of the possible information provided by a graph like 3a. Typically, when a kinetic profile goes through a maximum as a function of a given parameter it means that there is a change of the rate determining step governing the reaction mechanism. The mechanistic aspect is addressed in the paragraph related to the DFT calculations. Figure 3a illustrates two possible pathways for the catalytic mechanism: for both the rate determining is suggested to be the oxidation of the substrate (i.e. dehydrogenation of TMB, IIIb and IV, respectively). Thus, the authors appear to suggest there is not a change in the rate-determining step contrary to what is indicated by the graph of Figure 2b. Volcano-like plots observed in redox processes are associated with changes of the step which is controlling the reaction as discussed in a nice recent review (Chem. Rev., 2018, 118 (5), pp 2302–2312) to which the authors are referred to. To address this point is a must in order make their observation meaningful from a physic-chemical point of view.

2) Related to the previous point. The statement (p. 11, 3rd line) "…by altering their affinity to the reactive intermediate " is not clear and might imply that by changing the substrate the picture could change substantially.

3) According to what is reported in the "Methods" section, peroxidase-like activity is performed by dividing the slope of the change of absorbance at 652 nm in the first 60 seconds by the normalized BET area. Apart the normalization, the efficiency of the different nanozymes is hence determined from the initial rate in a time frame where Absorbance depends linearly on time (this, however, does not appear to be true in Fig. 1f). This is acceptable if the reaction is carried out under saturation conditions. If the catalyst is not saturated the affinity for the substrate affects the initial rate

introducing a variable that alters the meaning of the results. Are the authors sure they are operating under saturation conditions for all catalysts they have examined? Importantly, should eventually turn out that the activity the authors are determining is compounded with a binding contribution this will totally change the rationale behind their data.

In conclusion, in spite of the great efforts done by the authors in characterizing their system I do believe the manuscript, as presented, fails to explain the reason of the observed dependence of the activity from eg occupancy. As I said above this is a required information to warrant acceptance in Nature Communications.

Reviewers' Comments:

Reviewer #1 (Remarks to the Author):

The revised version of the paper "*e_g* occupancy as an effective descriptor for the catalytic activity of perovskite oxide-based peroxidase mimics" has in general satisfyingly addressed my concerns. The authors now provide more detailed investigation and explanation of the relationship between the e_g occupancy and superior POD-like nanozyme of perovskite oxide. They convincingly disclose the catalytic mechanism, by succeeded in providing more solid experimental evidence and theoretical discussions. The paper is well organized including writing. I thus recommend to publish the paper in the present form without alternation in Nature Communications.

Reply: We are grateful to the reviewer for the positive recognition of our work.

Reviewer #2 (Remarks to the Author):

[Redacted]

The idea of providing a valid descriptor to predict the reactivity of these catalysts is sound and a plot as that of Figure 2b represents an excellent starting point for introducing this concept. The comparison with other descriptors that fail to correlate with the extensive set of data collected by the authors is also a good indication that e_g occupancy is indeed a good parameter to take into consideration.

There are a number of things that have not been addressed yet in spite of the work the authors have done to increase the quality of their contribution in response to the first round of reviews.

Reply: We appreciate these insightful comments, which have helped to improve our work significantly. We have fully addressed the reviewer's concerns as follows.

1) As a physical organic chemist, I was struck by the poor (or missing) analysis of the possible information provided by a graph like 3a. Typically, when a kinetic profile goes through a maximum as a function of a given parameter it means that there is a change of the rate determining step governing the reaction mechanism. The mechanistic aspect is addressed in the paragraph related to the DFT calculations. Figure 3a illustrates two possible pathways for the catalytic mechanism: for both the rate determining is suggested to be the oxidation of the substrate (*i.e.*, dehydrogenation of TMB, IIIb and IV, respectively). Thus, the authors appear to suggest there is not a change in the rate-determining step contrary to what is indicated by the graph of Figure 2b. Volcano-like plots observed in redox processes are associated with changes of the step which is controlling the reaction as discussed in a nice recent review (Chem. Rev., 2018, 118 (5), pp 2302–2312) to which the authors are referred to. To address this point is a must in order make their observation meaningful from a physic-chemical point of view.

Reply: We agree with the reviewer's criticism on the rate-determining step and appreciate the insightful comments. According to the comments, we re-analyzed the calculated energetics and did find evidence for the change of rate-determining step. We re-plotted the original Supplementary Figure 25 as the new Supplementary Figure 24 (see also Figure R1), which showed the energies of species involved in the proposed reaction pathways. It suggested that for the five perovskites with e_g occupancy < 1.2 (*i.e.*, $LaCrO_3$, $CaMnO_3$, $La_{0.5}Sr_{0.5}MnO_3$, $LaMnO_3$, and $LaCoO_3$), which are all located on the left side of Figure 2b's volcano-like plots, the rate-determining step should be the oxidation of the substrate (*i.e.*, IIIb and IV of Figure 3a); for the other five with e_g occupancy > 1.2 (*i.e.*, $LaMn_{0.5}Ni_{0.5}O_3$, $SrFeO_3$, $La_{0.5}Sr_{0.5}FeO_{2.5}$, $La_{0.5}Sr_{0.5}FeO_3$, and $LaFeO_3$), which are all located on the right side of the volcano-like plots, the rate-determining step should be the O-O bond splitting of the adsorbed H_2O_2* (II of Figure 3a); for $LaNiO_3$ with e_g occupancy of 1.2, which is at the maximum point, the rate-determining step is also the O-O bond splitting of the adsorbed H_2O_2*.

In the revised manuscript, we have re-plotted the original Figure 2b by adding the equations (in grey color), which suggest the rate-determining steps for the corresponding perovskites. For your convenience, Figure 2b is presented below as Figure R2. We also added the related discussion in the revised manuscript.

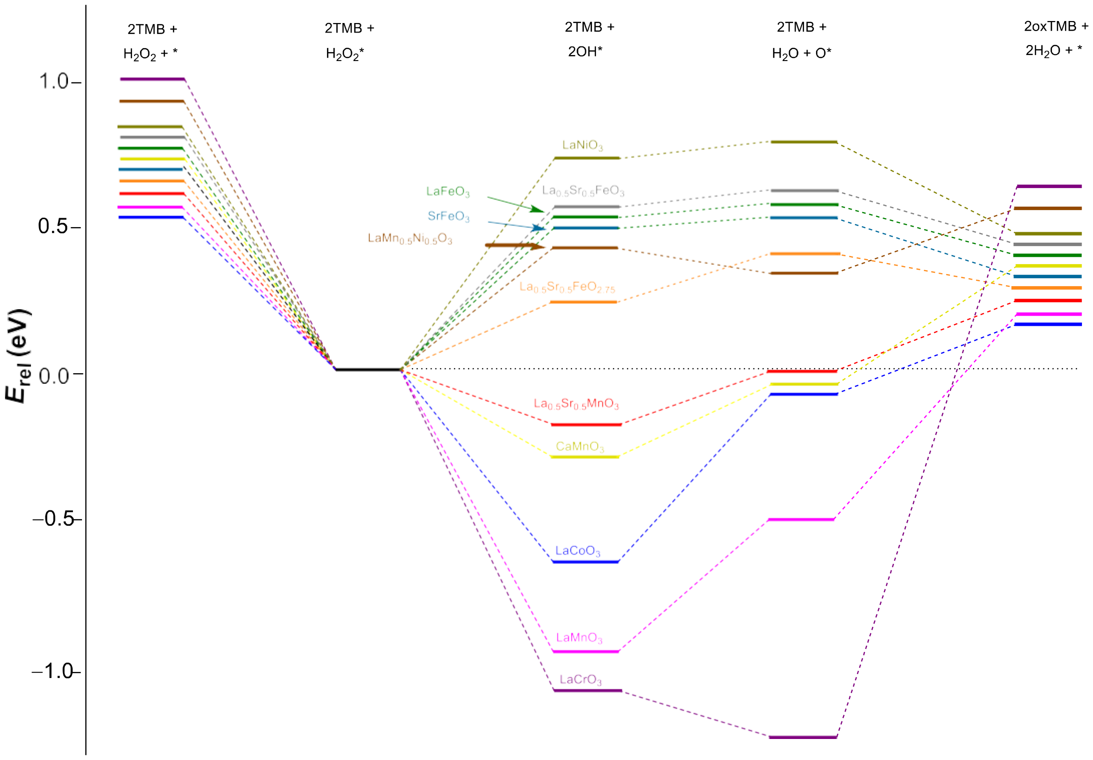

Figure R1. Relative energies for intermediates involved in the catalytic reactions proposed in Figure 3a.

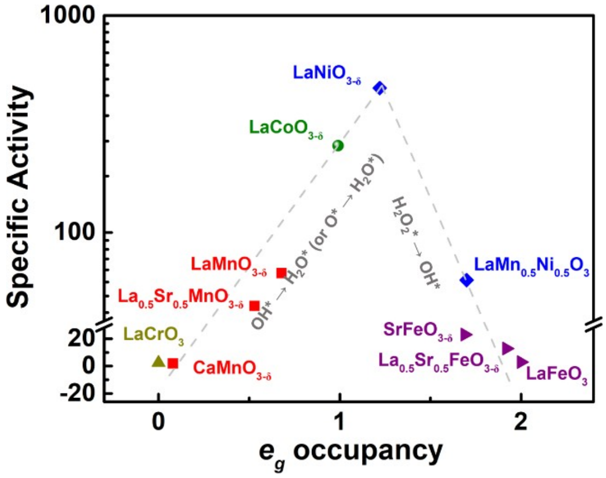

Figure R2. Specific peroxidase-like activities of perovskite TMOs plotted as a function of e_g occupancy, in which equations shown in grey are the rate-limiting reaction steps.

2) Related to the previous point. The statement (p. 11, 3rd line) "…by altering their affinity to the reactive intermediate "is not clear and might imply that by changing the substrate the picture could change substantially.

Reply: We agree with this criticism. The above-mentioned statement has been changed as follows: "*Taking these results together, e_g occupancy influences the peroxidase mimicking activities of perovskites by altering the E_{ads} of reaction intermediates and the rate-determining step governing the catalytic reactions.*"

3) According to what is reported in the "Methods" section, peroxidase-like activity is performed by dividing the slope of the change of absorbance at 652 nm in the first 60 seconds by the normalized BET area. Apart the normalization, the efficiency of the different nanozymes is hence determined from the initial rate in a time frame where Absorbance depends linearly on time (this, however, does not appear to be true in Fig. 1f). This is acceptable if the reaction is carried out under saturation conditions. If the catalyst is not saturated the affinity for the substrate affects the initial rate introducing a variable that alters the meaning of the results. Are the authors sure they are operating under saturation conditions for all catalysts they have examined? Importantly, should eventually turn out that the activity the authors are determining is compounded with a binding contribution this will totally change the rationale behind their data.

Reply: As the reviewer indicated, the peroxidase-like activity of nanozyme should be more properly measured when the catalytic reaction was carried out under saturating substrate conditions. To check whether our original conclusions are still valid under saturating substrate conditions, v_{max} (the maximal reaction velocity obtained under saturating substrate conditions) were measured (see **Notes** below for more details of the measurements). The mass activities of the nanozymes were defined as follows: $Mass\ Activity\ =\ v_{max}$ (1)

The specific activities of the nanozymes were defined as follows:

$$Specific\ Activity = \frac{Mass\ Activity}{Normolized\ BET\ Area} \quad (2)$$

$$Normalized\ BET\ Area = \frac{BET\ Area\ of\ Nanozyme}{BET\ Area\ of\ LaNiO_{3-\delta}} \quad (3)$$

Then, the newly measured catalytic activities (mass activity and specific activity) of nanozymes were re-plotted as a function of e_g occupancy. As shown in Figure R3, both mass activity and specific activity of perovskite TMOs still exhibited the same volcano dependence on the corresponding e_g occupancies as the original Figure 2b and the original Supplementary Figure 15b. These results further validated the conclusion that the catalytic activity of the perovskite TMO-based peroxidase mimics is primarily governed by the e_g occupancy of the B cations.

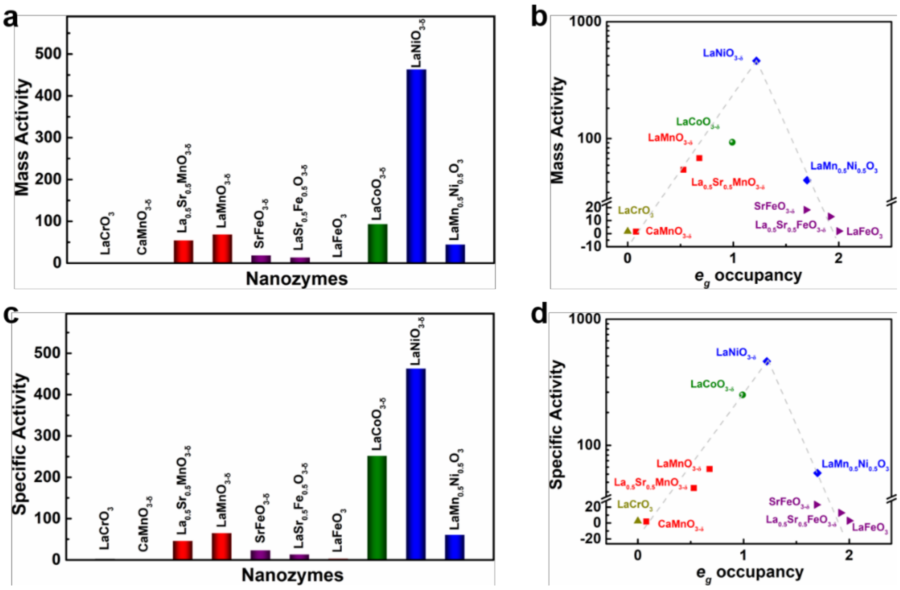

Figure R3. (**a**) Mass-based peroxidase-like activities of perovskite TMOs. (**b**) Mass-based peroxidase-like activities of perovskite TMOs plotted as a function of e_g occupancy. (**c**) Specific peroxidase-like activities of perovskite TMOs. (**d**) Specific peroxidase-like activities of perovskite TMOs plotted as a function of e_g occupancy. The two lines are shown for eye-guiding only.

The re-calculated catalytic activity of $LaNiO_{3-\delta}$ was compared with other typical nanozymes. As shown in Figure R4, the $LaNiO_{3-\delta}$ exhibited superior performance than other nanozymes in terms of both mass and specific activity, which was also consistent with the original Figure 5c and 5d.

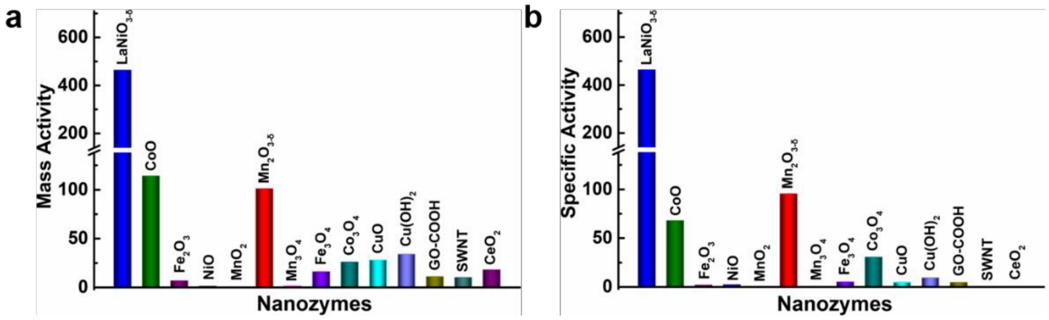

Figure R4. (**a**) Mass-based peroxidase-like activities of $LaNiO_{3-\delta}$ and other nanozymes. (**b**) Specific peroxidase-like activities of $LaNiO_{3-\delta}$ and other nanozymes.

All the figures involved in catalytic activity were re-plotted using the newly calculated catalytic activity in the revised manuscript.

Notes on the measurements of v_{max}:

To evaluate the catalytic activity of nanozyme more properly, steady-state kinetics assays were carried out to obtain the kinetics parameters (*i.e.*, v_{max} and K_m). Steady-state kinetics assays were conducted at 37 °C in 1.0 mL cuvettes with a path length of 0.2 cm. A 0.2 M NaOAc buffer solution (pH 4.5) was used as the reaction buffer and 10 µg/mL of nanozymes were used for their kinetics assays. The kinetics data were obtained by varying the concentration of H_2O_2 while keeping the TMB's concentration constant. The kinetics constants (*i.e.*, v_{max} and K_m) were calculated by fitting the reaction velocity values and the substrate concentrations to the "Michaelis-Menten" equation as follows:

$$v = \frac{v_{max} \times [S]}{K_m + [S]} \quad (4)$$

the "Michaelis-Menten" equation could be further converted to "Lineweaver–Burk" equation as follows:

$$\frac{1}{v} = \frac{K_m}{v_{max}} \cdot \frac{1}{[S]} + \frac{1}{v_{max}} \quad (5)$$

where υ is the initial reaction velocity and v_{max} is maximal reaction velocity that is obtained under saturating substrate conditions. [*S*] is the substrate concentration. K_m, the Michaelis constant, equals to the concentration of substrate when the initial reaction velocity reaches half of its maximal reaction rate.

As the reviewer indicated, the initial reaction velocity should be determined only when the

absorbance at 652 nm depends linearly on the reaction time. However, this does not appear to be true for $SrFeO_{3-\delta}$ (curve 2). We checked the kinetics curve of $SrFeO_{3-\delta}$ and TMB in the absence of H_2O_2. As shown in Figure R5a (curve 1), the reaction rate was fast within initial 10 s because TMB could be oxidized by $SrFeO_{3-\delta}$ directly. The curve 3 was obtained when curve 2 was used to subtract curve 1. The curve 3 showed a linear dependence on time and we used curve 3 to calculate the initial reaction velocity. For some materials, such as $LaNiO_{3-\delta}$, whose absorbance exhibited a good linear relationship versus time, the kinetic curves of A_{652} could be used to calculate the initial reaction velocity directly (Figure R5b).

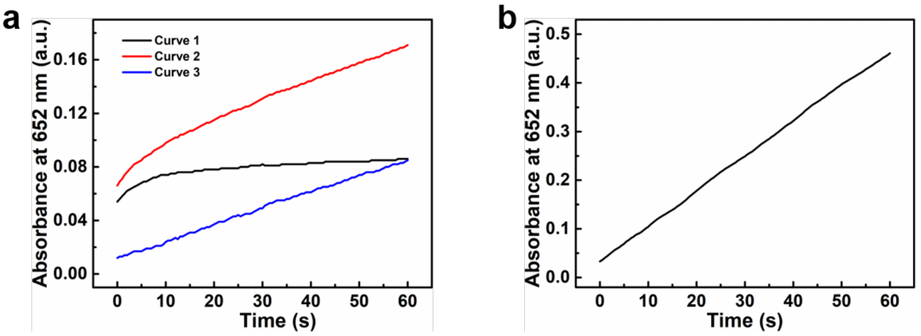

Figure R5. (**a**) Kinetic curves of A_{652} for monitoring the different reaction systems in the presence of $SrFeO_{3-\delta}$ nanozyme. Curve 1: 10 μg/mL $SrFeO_{3-\delta}$ + 1 mM TMB; Curve 2: 10 μg/mL $SrFeO_{3-\delta}$ + 100 mM H_2O_2 + 1 mM TMB; Curve 3: curve 2 minus curve 1. (**b**) Kinetic curves of A_{652} for monitoring the different reaction systems in the presence of 1 mM TMB and 100 mM H_2O_2 with 10 μg/mL $LaNiO_{3-\delta}$.

After the initial reaction velocity υ versus various concentrations of H_2O_2 was obtained, typical profile for "Michaelis-Menten" kinetics equation was obtained (Figure R6a and R6b). When double reciprocal plots of the velocity were plotted versus different concentration of H_2O_2, typical profile for "Lineweaver–Burk" equation was obtained (Figure R6c and R6d). The kinetics parameters of v_{max} and K_m could be obtained from the fitting curves in Figure R6c and R6d.

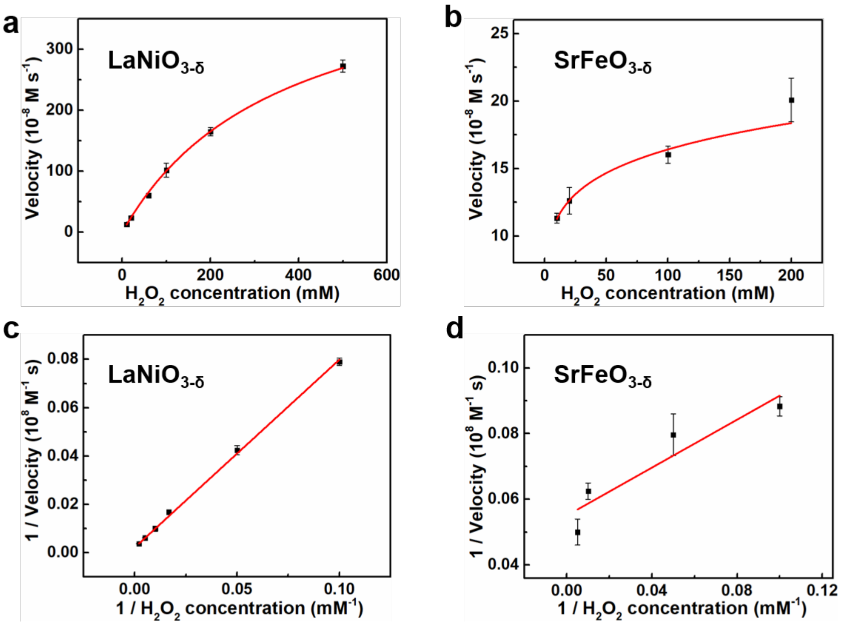

Figure R6. The steady-state kinetic assays of $LaNiO_{3-\delta}$ and $SrFeO_{3-\delta}$ nanozymes. (**a**, **b**) Plots of the velocity of the reaction versus different concentrations of H_2O_2 for $LaNiO_{3-\delta}$ and $SrFeO_{3-\delta}$ nanozymes. (**c**, **d**) Double reciprocal plots of the velocity versus varying concentration of H_2O_2 for $LaNiO_{3-\delta}$ and $SrFeO_{3-\delta}$ nanozymes.

The kinetics parameters of other nanozymes were determined using the same method as $LaNiO_{3-\delta}$ and $SrFeO_{3-\delta}$. The detailed kinetics parameters obtained by steady-state kinetics assays were listed in Table R1. As for TMOs with negligible activity (*i.e.*, $LaCrO_3$, $LaFeO_3$, $CaMnO_{3-\delta}$, NiO, MnO_2, and Mn_3O_4), we assumed the initial reaction velocity in the presence of 10 μg/mL nanozymes, 1 mM TMB and 100 mM H_2O_2 as the v_{max} because the kinetics measurements for them were difficult and not reliable.

Table R1. Kinetics parameters of TMOs.

Catalyst	Substrate	K_m (mM)	v_{max} (Ms^{-1})
**$SrFeO_{3-\delta}$**	H_2O_2	6.05	0.18×10^{-6}
**$La_{0.5}Sr_{0.5}MnO_{3-\delta}$**	H_2O_2	40.08	0.54×10^{-6}
**$La_{0.5}Sr_{0.5}FeO_{3-\delta}$**	H_2O_2	8.14	0.13×10^{-6}
**$LaNiO_{3-\delta}$**	H_2O_2	359.92	4.63×10^{-6}
**$LaNiO_{3-\delta}$-H_2**	H_2O_2	125.96	1.45×10^{-6}
**$LaMnO_{3-\delta}$**	H_2O_2	31.43	0.68×10^{-6}
**$LaMn_{0.5}Ni_{0.5}O_3$**	H_2O_2	112.84	0.44×10^{-6}
**CoO**	H_2O_2	92.10	1.14×10^{-6}
**$LaCoO_{3-\delta}$**	H_2O_2	205.45	0.93×10^{-6}
**SWNT**	H_2O_2	2.41	0.102×10^{-6}
**GO-COOH**	H_2O_2	23.66	0.11×10^{-6}
**CeO_2**	H_2O_2	4.41	0.18×10^{-6}
**Fe_2O_3**	H_2O_2	75.97	0.068×10^{-6}
**Fe_3O_4**	H_2O_2	41.66	0.16×10^{-6}
**CuO**	H_2O_2	31.18	0.28×10^{-6}
**Co_3O_4**	H_2O_2	41.75	0.26×10^{-6}
**Mn_2O_3**	H_2O_2	12.53	1.01×10^{-6}
**$Cu(OH)_2$**	H_2O_2	28.91	0.34×10^{-6}
**$LaCrO_3$**	H_2O_2	/	0.017×10^{-6}
**$LaFeO_3$**	H_2O_2	/	0.019×10^{-6}
**$CaMnO_{3-\delta}$**	H_2O_2	/	0.015×10^{-6}
**NiO**	H_2O_2	/	0.011×10^{-6}
**MnO_2**	H_2O_2	/	0.0064×10^{-6}
**Mn_3O_4**	H_2O_2	/	0.013×10^{-6}

Reviewer #2 (Remarks to the Author):

I am glad to see my points properly addressed in this re-revised version of the manuscript. In particular, the change of the rate-determining step in the mechanism is now in line with what has been reported by other laboratories studying similar reactions.
Furthermore, the fact that the binding does not affect the analysis of the data hints to the fact that, likely, affinity constants are not much different (the authors have been lucky!).
In view of the changes introduced I clear the paper for publication in its present form.
